# Alu Elements in *ANRIL* Non-Coding RNA at Chromosome 9p21 Modulate Atherogenic Cell Functions through *Trans*-Regulation of Gene Networks

**DOI:** 10.1371/journal.pgen.1003588

**Published:** 2013-07-04

**Authors:** Lesca M. Holdt, Steve Hoffmann, Kristina Sass, David Langenberger, Markus Scholz, Knut Krohn, Knut Finstermeier, Anika Stahringer, Wolfgang Wilfert, Frank Beutner, Stephan Gielen, Gerhard Schuler, Gabor Gäbel, Hendrik Bergert, Ingo Bechmann, Peter F. Stadler, Joachim Thiery, Daniel Teupser

**Affiliations:** 1 LIFE – Leipzig Research Center for Civilization Diseases, Universität Leipzig, Leipzig, Germany; 2 Institute of Laboratory Medicine, Clinical Chemistry and Molecular Diagnostics, University Hospital Leipzig, Leipzig, Germany; 3 Institute of Laboratory Medicine, Ludwig-Maximilians-University Munich, Munich, Germany; 4 Transcriptome Bioinformatics Group and Interdisciplinary Centre for Bioinformatics, University Leipzig, Leipzig, Germany; 5 Institute for Medical Informatics, Statistics and Epidemiology, University Leipzig, Leipzig, Germany; 6 Interdisciplinary Center for Clinical Research, University Leipzig, Leipzig, Germany; 7 Department of Internal Medicine/Cardiology, Heart Center, University Leipzig, Leipzig, Germany; 8 Department of General, Thoracic, and Vascular Surgery, University Dresden, Dresden, Germany; 9 Institute of Anatomy, University Leipzig, Leipzig, Germany; 10 Max Planck Institute for Mathematics in the Sciences, Leipzig, Germany; 11 Santa Fe Institute, Santa Fe, New Mexico, United States of America; University of Oxford, United Kingdom

## Abstract

The chromosome 9p21 (Chr9p21) locus of coronary artery disease has been identified in the first surge of genome-wide association and is the strongest genetic factor of atherosclerosis known today. Chr9p21 encodes the long non-coding RNA (ncRNA) *antisense non-coding RNA in the INK4 locus* (*ANRIL*). *ANRIL* expression is associated with the Chr9p21 genotype and correlated with atherosclerosis severity. Here, we report on the molecular mechanisms through which *ANRIL* regulates target-genes *in trans*, leading to increased cell proliferation, increased cell adhesion and decreased apoptosis, which are all essential mechanisms of atherogenesis. Importantly, *trans*-regulation was dependent on Alu motifs, which marked the promoters of *ANRIL* target genes and were mirrored in *ANRIL* RNA transcripts. *ANRIL* bound Polycomb group proteins that were highly enriched in the proximity of Alu motifs across the genome and were recruited to promoters of target genes upon *ANRIL* over-expression. The functional relevance of Alu motifs in *ANRIL* was confirmed by deletion and mutagenesis, reversing *trans*-regulation and atherogenic cell functions. *ANRIL*-regulated networks were confirmed in 2280 individuals with and without coronary artery disease and functionally validated in primary cells from patients carrying the Chr9p21 risk allele. Our study provides a molecular mechanism for pro-atherogenic effects of *ANRIL* at Chr9p21 and suggests a novel role for Alu elements in epigenetic gene regulation by long ncRNAs.

## Author Summary

Chromosome 9p21 is the strongest genetic factor for coronary artery disease and encodes the long non-coding RNA (ncRNA) *ANRIL*. Here, we show that increased *ANRIL* expression mediates atherosclerosis risk through *trans*-regulation of gene networks leading to pro-atherogenic cellular properties, such as increased proliferation and adhesion. *ANRIL* may act as a scaffold, guiding effector-proteins to chromatin. These functions depend on an Alu motif present in *ANRIL* RNA and mirrored several thousand-fold in the genome. Alu elements are a family of primate-specific short interspersed repeat elements (SINEs) and have been linked with genetic disease. Current models propose that either exonisation of Alu elements or changes of *cis*-regulation of adjacent genes are the underlying disease mechanisms. Our work extends the function of Alu transposons to regulatory components of long ncRNAs with a central role in epigenetic *trans*-regulation. Furthermore, it implies a pivotal role for Alu elements in genetically determined vascular disease and describes a plausible molecular mechanism for a pro-atherogenic function of *ANRIL* at chromosome 9p21.

## Introduction

**Figure 1:**
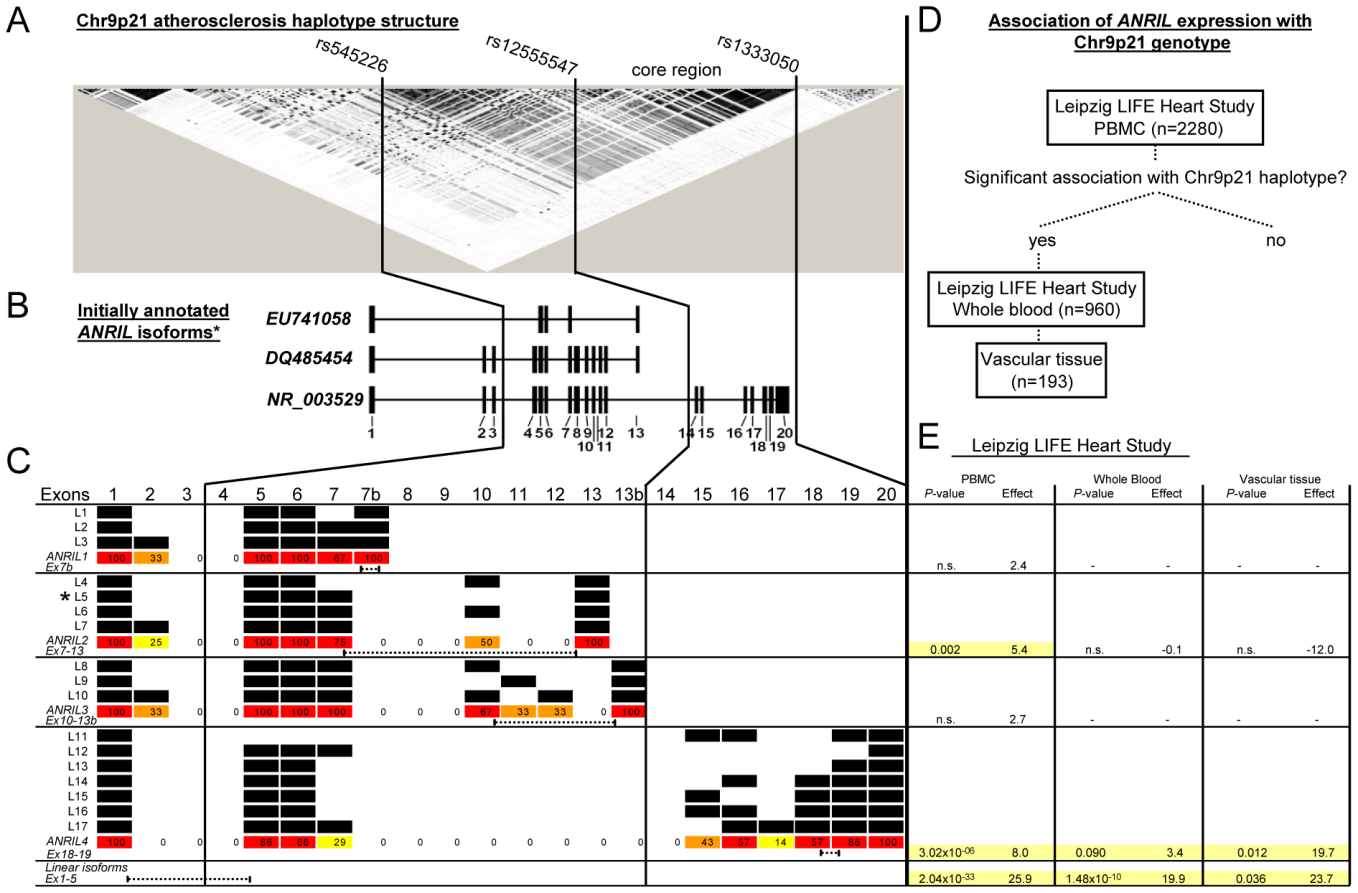
Annotated *ANRIL* transcripts in the Chr9p21 region, transcript structure and association of *ANRIL* isoforms with Chr9p21 genotype. (A) Chr9p21 haplotype structure (HapMap CEU, r^2^) and core atherosclerosis region (between rs12555547 and rs1333050). (B) Exons of initially discovered *ANRIL* transcripts and their relative position in the Chr9p21 region. *A full list of currently annotated *ANRIL* isoforms is given in Figure S1B. (C) *ANRIL* transcripts identified by RACE and PCR amplification (L1-L17). 4 major isoform groups with 4 distinct transcriptions ends were identified. Frequency of exon occurrence/isoform group is color-coded and given in %. *ANRIL1-4* denote highly expressed consensus transcripts harbouring exons found in >50% transcripts of the respective isoform group (red). Dotted lines indicate positions of transcript-specific qRT-PCR assays. (D) Study design of *ANRIL* expression and association with Chr9p21. (E) Association of *ANRIL* isoforms with Chr9p21 in human PBMC, whole blood and vascular tissue (effect, % change/risk allele defined by rs10757274, rs2383206, rs2383297, and rs10757278).

The chromosome 9p21 (Chr9p21) locus is the strongest genetic risk factor of atherosclerosis known today, yet, the responsible mechanisms still remain unclear. Chr9p21 lacks associations with common cardiovascular risk factors, such as lipids and hypertension, indicating that the locus exerts its effect through an alternative mechanism [1]–[4]. The risk region spans ∼50 kb of DNA sequence and does not encode protein-coding genes but the long non-coding RNA (ncRNA) *antisense non-coding RNA in the INK4 locus* (*ANRIL*; Figure 1A) [5], [6]. *CDKN2BAS* or *CDKN2B-AS1* are used as synonyms for *ANRIL*. The closest neighbouring genes are the *cyclin-dependent kinase inhibitors CDKN2A* and *CDKN2B*, which are located ∼100 kb proximal of the Chr9p21 atherosclerosis risk region. While these genes are expressed in atherosclerotic lesions [7], the majority of studies in humans speak against a *cis*-regulation of *CDKN2A* and *CDKN2B* by Chr9p21 (reviewed by [8]). Studies in mice revealed no effect on atherosclerosis development [9], [10]. In contrast, a clear association of *ANRIL* with the Chr9p21 genotype has been established in several studies, even though the direction of effects is still a matter of dispute [5], [6], [8], [11]–[13]. Moreover, a correlation of *ANRIL* expression with atherosclerosis severity has been described [2], [8]. Based on these clinical and experimental data, *ANRIL* must be considered as a prime functional candidate for modifying atherosclerosis susceptibility at the Chr9p21 locus.

*ANRIL* belongs to the family of long ncRNAs, which are arbitrarily defined and distinguished from short ncRNA, such as microRNA, by their length of >200 bp [14]–[16]. Long ncRNAs have been implicated in diverse functions in gene regulation, such as chromosome dosage-compensation, imprinting, epigenetic regulation, cell cycle control, nuclear and cytoplasmic trafficking, transcription, translation, splicing and cell differentiation [15], [17]–[19]. These effects are mediated by RNA-RNA, RNA-DNA or RNA-protein interactions [17]–[19]. Previous mechanistic work on *ANRIL* in prostate tissue and cell lines has focused on its role in *cis*-suppression of *CDKN2A* and *CDKN2B* [3], [20]. Using RNAi against *ANRIL*, these studies showed impaired recruitment of chromobox homolog 7 (CBX7), a member of Polycomb repressive complex 1 (PRC1) [3], and of suppressor of zeste 12 (SUZ12), a member of PRC2 [20], to the Chr9p21 region. PRCs are multiprotein complexes, responsible for initiating and maintaining epigenetic chromatin modifications and thereby controlling gene expression [21]. Yap et al found that knock-down of *ANRIL* decreased trimethylation of lysine 27 residues in histone 3 (H3K27me3) and was associated with increased *CDKN2A* expression, while *CDKN2B* remained unchanged [3]. In contrast, Kotake et al showed that shRNA-mediated *ANRIL* knock-down disrupted SUZ12 binding to the Chr9p21 locus and led to increased *CDKN2B* expression whereas *CDKN2A* remained unaffected [20]. While results of *ANRIL* knock-down are conflicting with regard to expression of *CDKN2A* and *CDKN2B*, both studies demonstrated a significant reduction of cell proliferation [3], [20], a key mechanism in atherogenesis [22]. In these studies, however, potential effects of *ANRIL* knock-down on *trans*-regulation were not investigated.

Sato et al transiently over-expressed one specific *ANRIL* transcript in HeLa cells and found effects on expression levels of various genes *in trans* [23]. Even though the molecular mechanisms were not investigated in that work, this finding was of interest because *trans*-regulation of target genes has been proposed as a key mechanism for biological effects of other long non-coding RNA such as *HOTAIR* [24]–[26]. It is believed that these long ncRNAs mediate their effects through targeting epigenetic modifier proteins to specific sites in the genome [17], [19], [27]. Taken together, the previously available data suggested that *ANRIL* might influence gene expression by modulating chromatin modification and thereby affect cardiovascular risk.

The aim of the present study was to investigate the role of *ANRIL* in gene regulation and cellular functions related to atherogenesis on a mechanistic level. To this end, we performed genome-wide expression analyses in cell lines over-expressing distinct *ANRIL* transcripts that were associated with Chr9p21. We studied the molecular mechanisms of *ANRIL*-mediated gene regulation by investigating *ANRIL* binding to epigenetic effector proteins and their distribution across the genome. Using bioinformatics studies, we identified a regulatory motif characteristic for *ANRIL*-regulated genes. Finally, the functional relevance of the motif was confirmed by deletion and mutagenesis and results were validated in primary human cells from patients with and without the Chr9p21 atherosclerosis risk allele.

## Results

### *ANRIL* isoforms *trans*-regulate target genes and modulate mechanisms of atherogenesis

**Figure 2:**
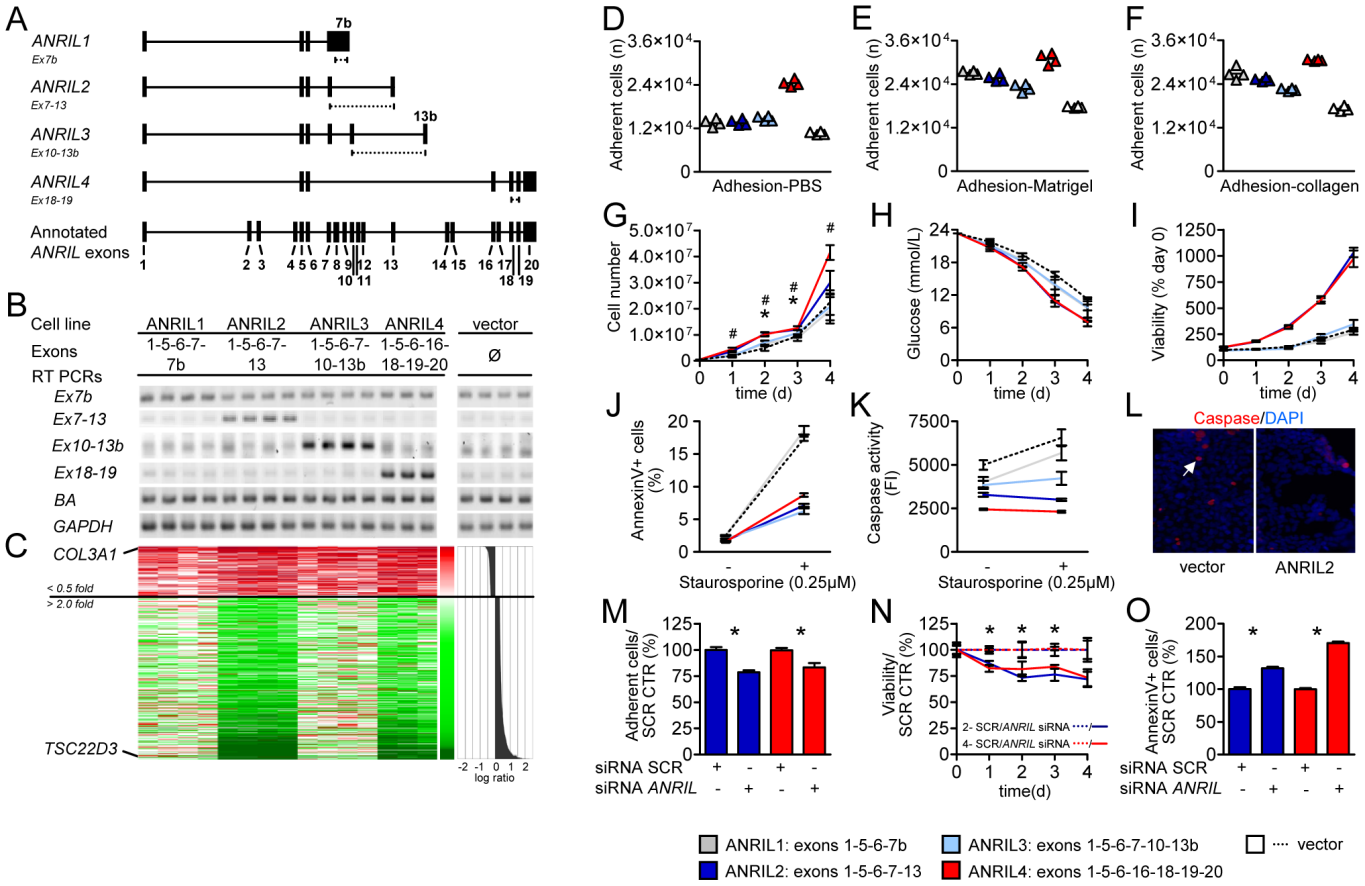
*ANRIL* regulates gene expression in t*rans* and affects cell adhesion, metabolic activity, proliferation, and apoptosis. (A) Consensus transcripts of 4 *ANRIL* isoform groups identified by RACE and PCR (Figure S1). Dotted lines indicate positions of transcript-specific qRT-PCR assays. (B) RT-PCR confirmation of *ANRIL* over-expression in stable ANRIL1-4 cell lines. 3–4 cell lines per *ANRIL* isoform were established, no effect on house-keeping gene expression was found (*beta-actin* (*BA*); *glyceraldehyde-3-phosphate dehydrogenase* (*GAPDH*)). (C) Heatmap of *ANRIL trans*-regulated transcripts corresponding to panel B. Transcripts with average down- (<0.5-fold, red) and up- (>2-fold, green) regulation relative to vector control are shown. (D–F) Adhesion of *ANRIL* over-expressing cell lines to PBS-, Matrigel-, and collagen-coated wells (*P*<0.01 for comparison of ANRIL1, 2, 3, 4 vs. vector control). (G–I) Cell proliferation and metabolic activity determined by (G) absolute cell numbers, (*/^#^ *P*<0.05 for ANRIL2 and ANRIL4 vs. vector control), (H) glucose utilisation, and (I) viability assay. (J–L) Apoptosis determined by (J) AnnexinV-positive cells, (K) caspase activity, and (L) caspase-3 staining. (H–K) *P*<0.05 for ANRIL2 and ANRIL4 vs. vector control. (M–O) Reversal of effects by RNAi against *ANRIL* (*P<0.05; SCR- scrambled control). (D–K) At least triplicate measurements per pool of 3–4 biological replicates were performed. For details on experimental setup and *P*-values please see Table S3. (M–O) n = 3/group. Validation of siRNA knock-down is shown in Figure S6. Error bars indicate s.e.m.

Using rapid amplification of cDNA ends (RACE) and subsequent PCR experiments, we identified four major groups of *ANRIL* transcripts in human peripheral blood mononuclear cells (PBMC) and the monocytic cell line MonoMac (Figure S1, Figure 1C). Consensus transcripts designated *ANRIL1-4*, comprising the most frequently occurring exon-combinations and most strongly expressed in MonoMac cells (Figure S1E), are shown in Figure 2A. Association of these transcripts with Chr9p21 was confirmed in PBMC (n = 2280) and whole blood (n = 960) of patients with and without coronary artery disease (CAD) in the Leipzig LIFE Heart Study [28] and in endarterectomy specimens (n = 193) (Figures 1D and 1E). The Chr9p21 risk allele was associated with increased *ANRIL* expression (Figure 1E) and different isoforms were positively correlated with each other (Figure S2). Using an assay detecting a common exon-exon boundary present in the majority of *ANRIL* isoforms (*Ex1-5*), we found a 26% overall increase of *ANRIL* expression per CAD-risk allele (*P* = 2.04×10^−33^) in PBMC of the Leipzig LIFE Heart Study. Strongest isoform specific effects were found for *ANRIL2* (5% increase per risk allele; *P* = 0.002) and *ANRIL4* (8% increase per risk allele; *P* = 3.02×10^−6^) (Figure 1E). To investigate the functional role of distinct *ANRIL* transcripts, we generated stably over-expressing cells lines (Figure 2B, Figure S3). *ANRIL* over-expression led to significant changes of gene expression *in trans* as determined by genome-wide mRNA expression analysis (Figure 2C). 219 transcripts were down- and 708 transcripts were up-regulated in ANRIL1-4 cell lines with average fold-changes smaller than 0.5 and greater than 2 compared to vector control, respectively (Table S1 and Table S2). These genes were distributed across the genome and there was no evidence for regulation of *CDKN2A/B* in the Chr9p21 region (Figure S4). Gene set enrichment analysis predicted an effect of *ANRIL* over-expression on movement/adhesion, growth/proliferation and cell death/apoptosis (Table 1), which are central mechanisms of atherogenesis [22]. We therefore aimed to experimentally validate these predictions in *ANRIL* over-expressing cell lines. Studies confirmed that *ANRIL* led to increased cell adhesion with strongest effects observed for cell lines over-expressing *ANRIL4* (Figures 2D–2F). Over-expression of *ANRIL* further promoted cell growth and metabolic activity (Figures 2G–2I). Apoptosis, as determined by flow cytometric analysis of AnnexinV-positive cells (Figure 2J), caspase-3 activity (Figure 2K) and caspase-3 immunohistochemical staining (Figure 2L) was attenuated. Greatest biological effects were consistently found in cell lines over-expressing isoforms *ANRIL2* and *4*, the same isoforms, which also revealed the strongest associations with the Chr9p21 risk genotype (Figure 1E). Moreover, we demonstrated a dose-dependent effect on these mechanisms in independently established cell lines over-expressing these isoforms (Figure S5). Effects on cell adhesion, proliferation and apoptosis could be reversed by RNAi-mediated knock-down of *ANRIL* as shown in ANRIL2 and ANRIL4 cell lines (Figures 2M–2O, Figure S6), further supporting a pivotal role of *ANRIL* in these pro-atherogenic cellular functions.

**Table 1:** Gene set enrichment analysis of *ANRIL trans*-regulated genes in cell lines ANRIL1-4.

Function	ANRIL1	ANRIL2	ANRIL3	ANRIL4
Cellular Development	1.43×10^−04^	7.02×10^−12^	3.43×10^−04^	2.52×10^−09^
Cellular Movement/Adhesion	4.53×10^−03^	4.21×10^−06^	2.18×10^−04^	1.67×10^−07^
Cellular Growth and Proliferation	1.71×10^−05^	5.24×10^−09^	4.90× 10^−06^	9.91×10^−07^
Cell Death/Apoptosis	6.27×10^−04^	8.29×10^−08^	2.38×10^−05^	4.54×10^−06^
Cell-To-Cell Signaling and Interaction	3.49×10^−03^	3.00×10^−04^	2.14×10^−04^	1.52×10^−05^
Cellular Assembly and Organization	1.21×10^−03^	5.77×10^−04^	9.71×10^−04^	3.63×10^−05^
Cellular Function and Maintenance	1.21×10^−03^	4.78×10^−04^	7.29×10^−04^	7.21×10^−05^
Gene Expression	1.21×10^−03^	1.83×10^−03^	2.95×10^−04^	1.32×10^−04^
Cell Morphology	5.01×10^−03^	6.65×10^−04^	5.99×10^−04^	7.46×10^−04^

Genes with expression changes of <0.5 and >2 in *ANRIL* over-expressing cell lines 1–4 compared to vector control were included in the analysis: ANRIL1- n = 893 (<0.5 n = 439/>2 n = 454 compared to control), ANRIL2- n = 2658 (<0.5 n = 1116/>2 n = 1542 compared to control), ANRIL 3- n = 1830 (<0.5 n = 1054/>2 n = 776 compared to control), and ANRIL4- n = 2982 (<0.5 n = 1514/>2 n = 1468 compared to control). *P*-values for enrichment of *trans*-regulated genes (www.ingenuity.com) are given.

### PRC but not CoREST/REST proteins bind to *ANRIL* and are recruited to target gene promoters upon *ANRIL* over-expression

**Figure 3:**
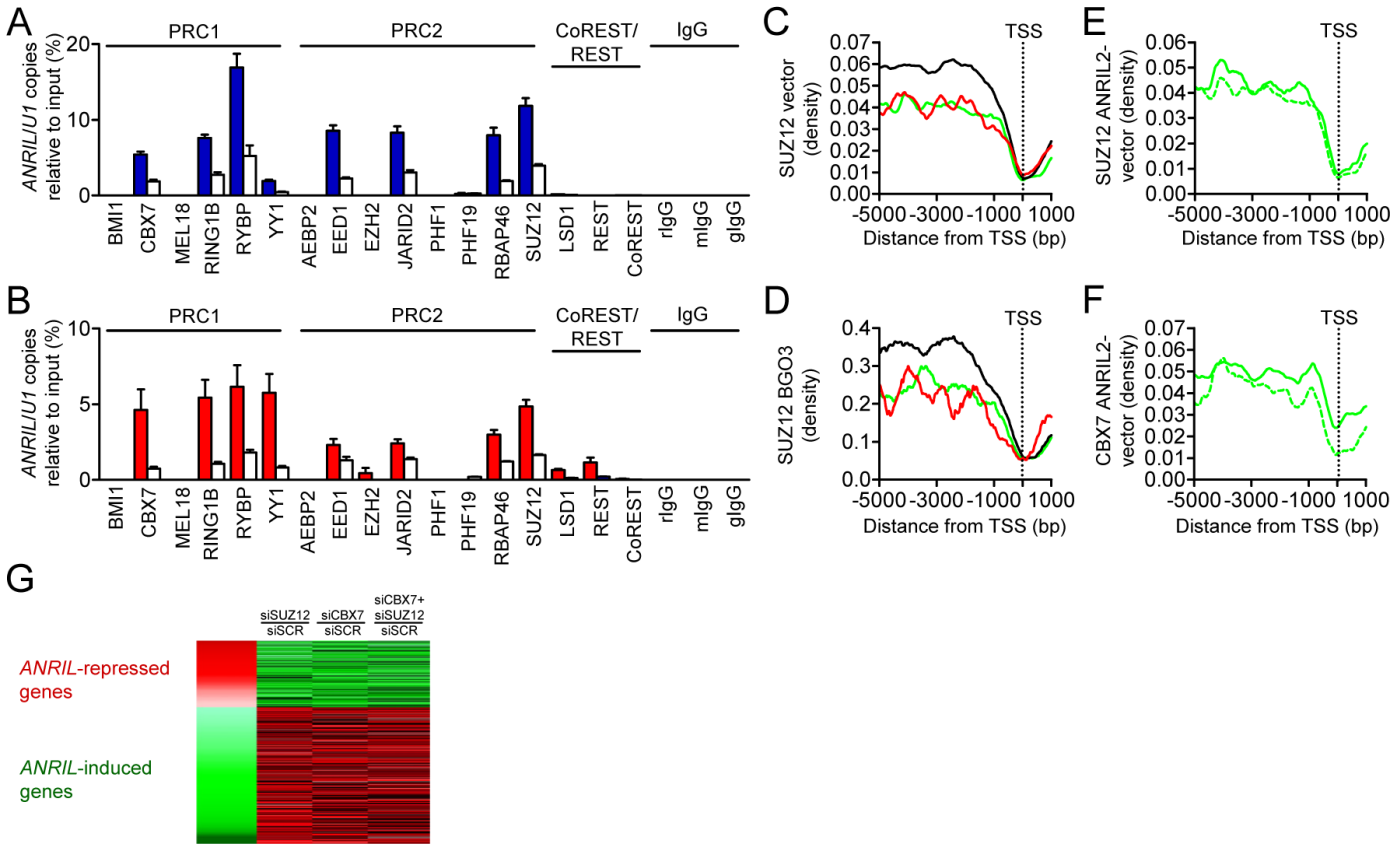
*ANRIL* binds to PRC1 and 2 proteins and recruits CBX7 and SUZ12 to promoters of target genes. (A, B) RNA immunoprecipitation (RIP) followed by qRT-PCR demonstrating *ANRIL* binding to PRC but not to CoREST/REST proteins in (A) ANRIL2 and (B) ANRIL4 cells. Copies of ANRIL relative to input control are given in (A) blue and (B) red, nuclear ncRNA *U1* (white) was used as negative control. rIgG/mIgG/gIgG- rabbit/mouse/goat IgG controls. Error bars indicate s.e.m. (C,D) SUZ12 binding in promoters of *ANRIL* up-(green), down-(red), and not (black) regulated genes in (C) vector control cell line and (D) in BGO3 cells (GSM602674). TSS- transcription start site. (E, F) Effect of *ANRIL* over-expression on (E) SUZ12 and (F) CBX7 binding in promoters of up-regulated genes (vector control- dotted line vs. ANRIL2- straight line). (G) Reversal of *ANRIL trans*-regulation by RNAi against SUZ12 and CBX7 in ANRIL2 cells. SCR- scrambled siRNA control.

To systematically identify *ANRIL*-associated epigenetic effector proteins, we next screened *ANRIL* binding to Polycomb group (PcG) proteins (AEBP2, BMI1, CBX7, EED, EZH2, JARID2, MEL18, PHF1, PHF19, RBAP46, RING1B, RYBP, SUZ12, YY1) and CoREST/REST (CoREST, REST, LSD1) using RNA immunoprecipitation (RIP) in nuclear extracts from ANRIL2 and 4 cells (Figures 3A–3B). *ANRIL* did not bind to CoREST/REST repressor proteins but bound to PRC1 proteins CBX7and RING1B and to PRC2 proteins EED, JARID2, RBAP46, and SUZ12. *ANRIL* also bound to PRC-associated proteins RYBP and YY1 which have been shown to induce gene expression [29], [30]. To investigate genome-wide distribution of Polycomb complexes, we chose CBX7 [3] and SUZ12 [20] as representative PcG proteins and performed chromatin immunoprecipitation followed by high-throughput sequencing (ChIP-seq) in ANRIL2 cells. Analysis of PcG distribution patterns in *ANRIL*-target gene promoters revealed reduced SUZ12 and CBX7 occupancy compared to not-regulated genes, following a wave-shaped binding pattern (Figure 3C and Figure S7). Reduced SUZ12 and CBX7 binding, as well as identical occupancy pattern, was replicated in publicly available data from BGO3 cells (Figure 3D). Over-expression of *ANRIL* increased SUZ12 and CBX7 binding to promoters of up-regulated genes (Figures 3E–3F), concordant with a recently described role of PRCs in regulation of active genes [31]. In further support, RNAi against CBX7 and SUZ12 largely reversed expression patterns not only of *ANRIL*-repressed, but also induced genes in ANRIL2 cells (Figure 3G). Whereas *ANRIL* binding to CBX7 and SUZ12 has previously been demonstrated in the context of *cis*-repression [3], [20], our experiments now extent the role of these proteins to *ANRIL*-mediated *trans*-regulation of gene networks pivotal in atherogenesis.

### Genome-wide PRC binding is dependent on an Alu motif marking promoters of *ANRIL* target-genes and contained within *ANRIL* RNA transcripts

**Figure 4:**
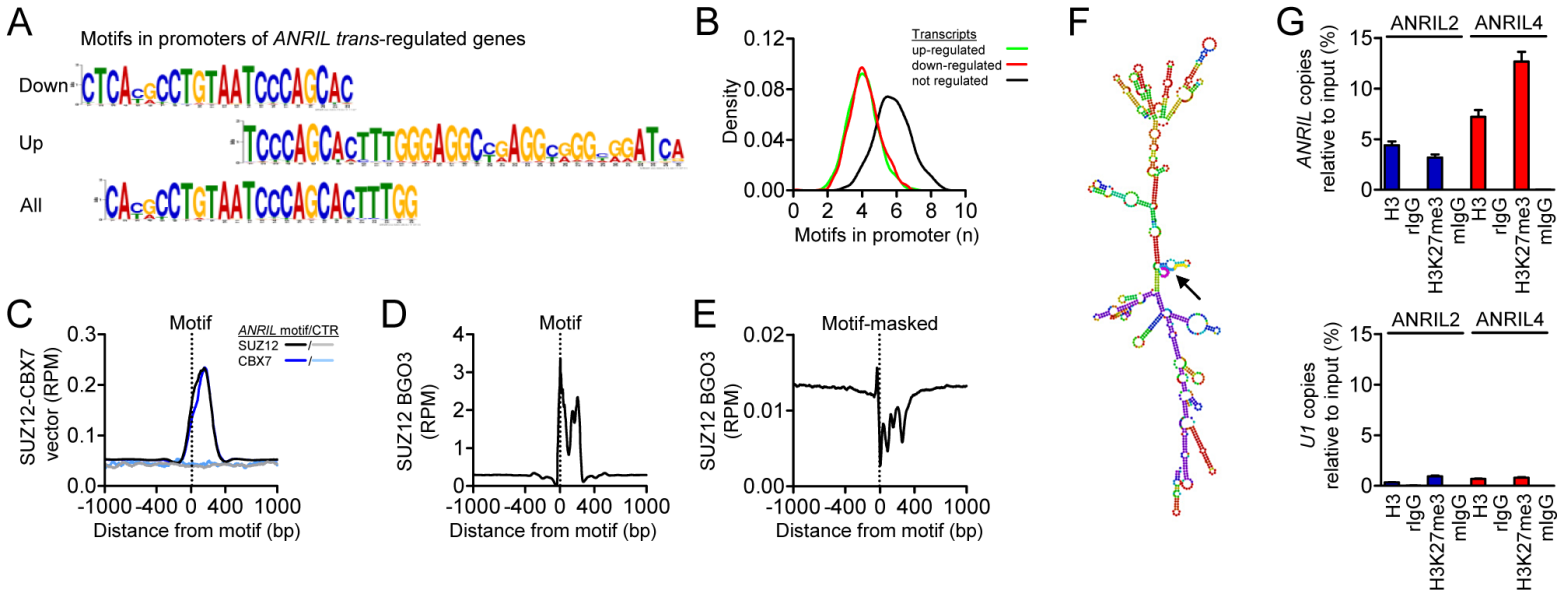
Identification of Alu motifs in promoters of *ANRIL trans*-regulated genes and *ANRIL* RNA and their spatial relation to PcG protein binding. (A) DNA motif in promoters (5 kb) of *trans*-regulated genes representing an Alu-DEIN repeat [33]. (B) Number of Alu motifs in promoters of *trans*- and not regulated transcripts (n per 5 kb). (C) ChIP-seq enrichment of SUZ12 and CBX7 binding distal to Alu motif, demonstrating a specific spatial relation of motif occurrence to PcG protein binding. RPM- reads per million mapped reads, CTR- random DNA control sequence. (D) Motif-associated SUZ12 signal peaks in an independent data set from BGO3 cells. The actual signal peak only becomes apparent, if a multiple matching policy is adopted. (E) Using a strict unique-matches only policy, a substantial signal reduction is seen downstream of the motif. Please note differences in y-axis scaling in (D, E). (F) Secondary RNA structure prediction for ANRIL2 using the Vienna RNA package [58]. Within the minimum free energy structure, the Alu-DEIN motif is located in a stem-loop structure (arrow). (G) RIP demonstrating *ANRIL* binding to histone H3 (H3) and trimethylated lysine 27 of histone 3 (H3K27me3) in ANRIL2 (blue) and ANRIL4 (red) cells. *U1* was used as negative control. mIgG/rIgG- mouse/rabbit IgG control. Error bars indicate s.e.m.

To address, which additional factors might be relevant for *ANRIL*-mediated *trans*-regulation, we performed bioinformatic analyses of promoter regions of *ANRIL* up- and down-regulated target genes. To this end, we used the MEME algorithm searching for differences in motif abundance and identified two partially overlapping DNA motifs (Figure 4A). Combined analysis of all *trans*-regulated genes validated the core motif CACGCCTGTAATCCCAGCACTTTGG (Figure 4A). The identified motif is an Alu-DEIN element [32], [33] with approximately 60.000 copies per human genome [34]. Since MEME does not provide information whether a motif is enriched or depleted, we quantitatively tested motif occurrence and found a significant depletion in up- and down-regulated genes compared to genes not regulated by *ANRIL* over-expression (4 vs. 6 occurrences per 5 kb promoter, respectively, *P*<10^−15^; Figure 4B). These data were consistent with decreased PcG occupancy in target-gene promoters (Figures 3C–3D). Notably, strong enrichment of PcG binding was found ∼150 bp downstream of the Alu motif compared to random DNA control (Figure 4C). This finding was replicated in the independent dataset for SUZ12 in BGO3 cells (Figure 4D) suggesting Alu element-dependent binding of PcG proteins as a general mechanism. Due to the repetitive nature of the investigated Alu motif, the significant spatial coherence between the motif and PcG occupancy was not detected when analyses were masked for repetitive elements (Figure 4E). Importantly, the same Alu motif which was found in the DNA promoter sequence of *ANRIL*-regulated genes was also present in *ANRIL* transcripts (Figure S8). Here, it was predicted to form a stem-loop structure in *ANRIL* RNA (Figure 4F) suggesting RNA-chromatin interactions as a potential effector mechanism [16]. Using RIP, we show that *ANRIL* co-immunoprecipitates with H3 and trimethylated lysine 27 of histone 3 (H3K27me3) (Figure 4G). These results speak further in favor of *ANRIL*-chromatin interaction at genomic sites where PRC-mediated epigenetic histone methylation takes place.

### Alu motif in *ANRIL* ncRNA is essential for *trans*-regulation and pro-atherogenic functions

**Figure 5:**
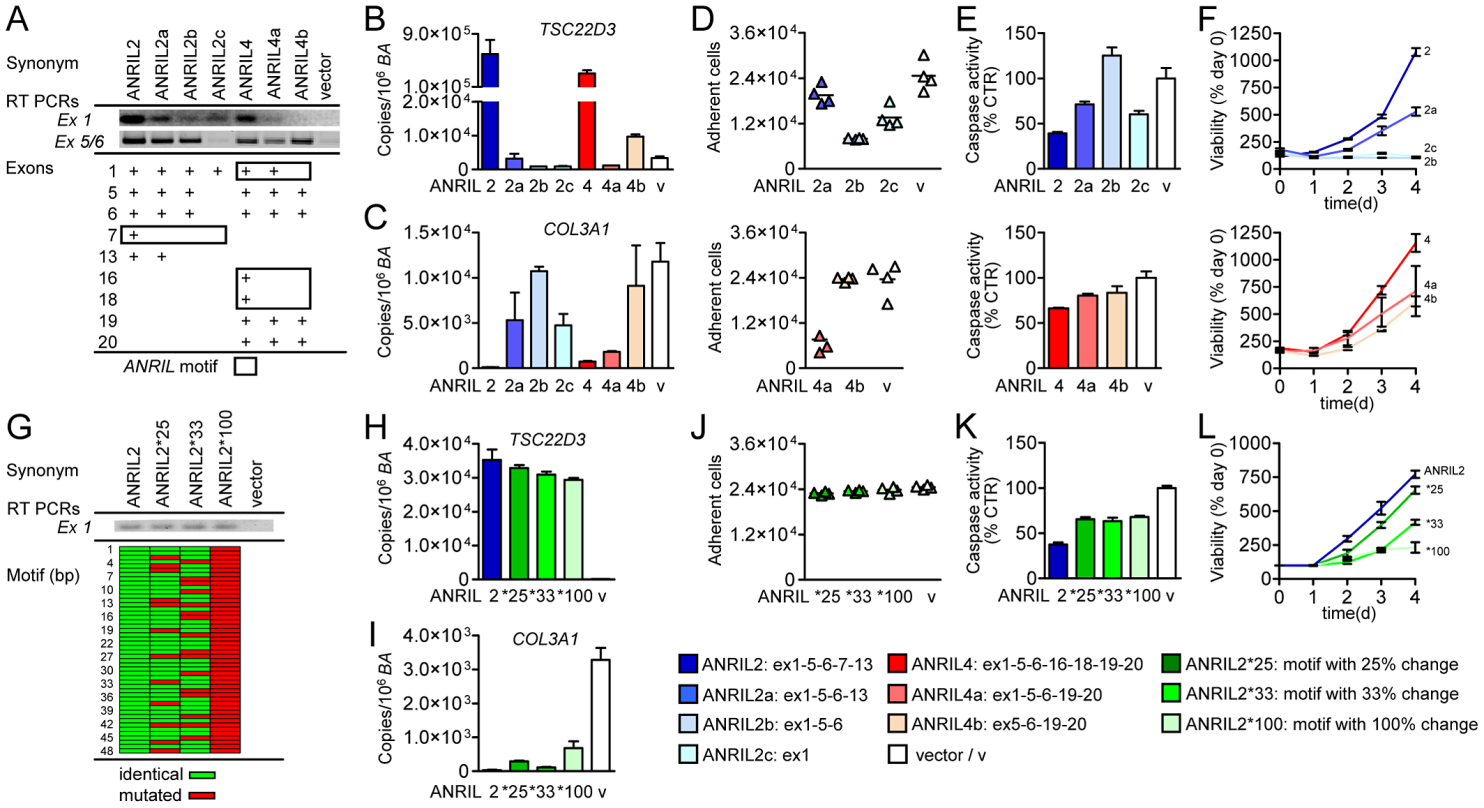
Pivotal role for Alu motif in *ANRIL* RNA function. (A) Cell lines over-expressing variants of *ANRIL2* (ANRIL2a, 2b, 2c) and *ANRIL4* (ANRIL4a, 4b) devoid of Alu motif sequences highlighted by boxes. Validation of over-expression using qRT-PCR assays. (B) Reversal of up-regulation (*TSC22D3*) and (C) down-regulation (*COL3A1*) of *ANRIL* target-gene mRNA expression in ANRIL2a-2c and ANRIL4a,4b compared to ANRIL2 and ANRIL4. Reversal of (D) cell adhesion, (E) apoptosis, and (F) proliferation in cell lines over-expressing mutant *ANRIL* isoforms. (G) Stable cell lines containing mutated forms of the Alu motif in *ANRIL2*. Positions of nucleotide exchanges (0, 25%, 33%, and 100%) in the 48 base-pair Alu motif are indicated in red. (H,I) Reversal of *trans*-regulation in mutant cell lines compared to ANRIL2. (J) Cell adhesion, (K) apoptosis, and (L) proliferation in mutant cell lines. (B,C,H,I,F,L) 3–4 biological replicates/isoform.(D,E,J,K) quadruplicate measurements per pool of 2–3 biological replicates. Error bars indicate s.e.m.

To validate the functional relevance of Alu sequences implemented in *ANRIL* transcripts, we generated stably over-expressing cell lines devoid of exons containing these sequences (Figure 5A, Figure S9). Over-expression of mutant isoforms ANRIL2a-c and ANRIL4a,b reversed expression changes of representative transcripts that were otherwise induced (*TSC22D3*; Figure 2C and Figure 5B) or suppressed (*COL3A1*; Figure 2C and Figure 5C) in ANRIL2 and 4 cells. Increased cell adhesion in ANRIL2 and 4 was abolished in cell lines lacking the Alu motif (Figure 5D). Consistent with this finding, ANRIL2a-c and ANRIL4a,b cell lines showed increased apoptosis and proliferated more slowly compared to ANRIL2 and 4 (Figures 5E–5F). To exclude that depletion of whole exons led to significant changes in *ANRIL* secondary structure and thus impaired *ANRIL* function, we next generated cell lines stably over-expressing *ANRIL* isoforms with single-base mutations of 25%, 33%, and 100% of nucleotides in the identified Alu motif (Figure 5G, Figure S9). Over-expression of these *ANRIL* isoforms confirmed the important role of the Alu motif by reversing expression changes of *ANRIL trans*-regulated genes (*TSC22D3*, Figure 2C and Figure 5H; *COL3A1*, Figure 2C and Figure 5I) compared to ANRIL2 cells. Moreover, *ANRIL*-mediated effects on adhesion, apoptosis and proliferation were attenuated (Figures 5J–5L), further supporting the functional relevance of *ANRIL* Alu motifs in pro-atherogenic cellular functions.

### Validation of *ANRIL*-associated gene networks and pro-atherogenic cell functions in primary cells of patients of the Leipzig LIFE Heart Study

**Figure 6:**
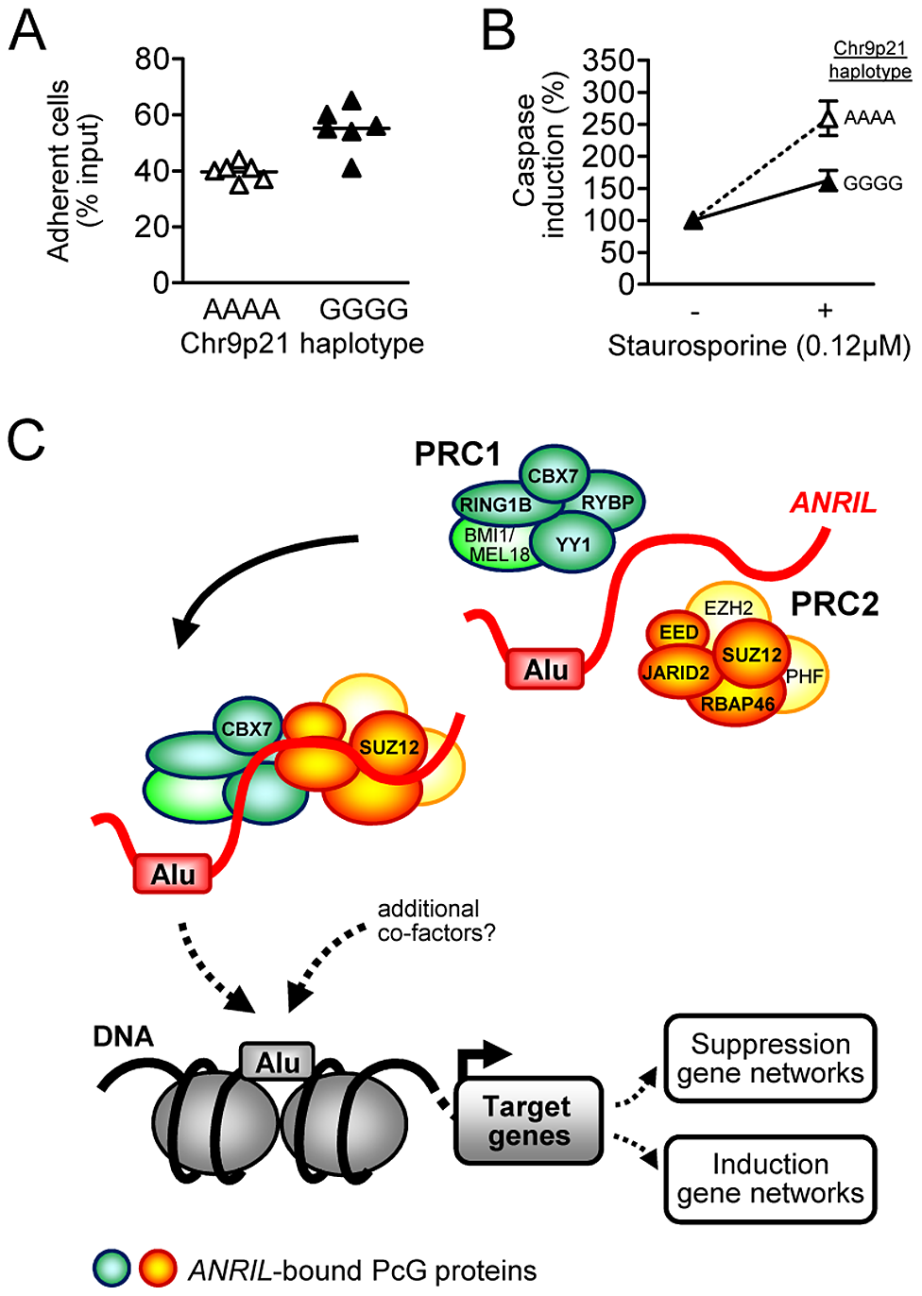
Validation of *ANRIL*-associated cellular effects in primary cells and schematic of molecular scaffolding by *ANRIL*. (A) PBMC from carriers of the Chr9p21 CAD-risk allele defined by rs10757274, rs2383206, rs2383297, and rs10757278 (n = 8) showed increased adhesion (*P* = 0.001) and (B) decreased apoptosis (*P* = 0.008) compared to cells of carriers of the protective allele (n = 8). (C) Schematic of molecular scaffolding by *ANRIL* mediated through potential chromatin-RNA interaction by Alu motifs.

To investigate whether findings from cell culture studies could be translated into the human situation, we investigated genome-wide transcript expression in PBMC from 2280 subjects of the Leipzig LIFE Heart Study and associated gene expression with the Chr9p21 haplotype and *ANRIL* expression. While no transcripts were significantly associated with Chr9p21 on a genome-wide level, gene set enrichment analyses of genes correlated with *ANRIL* expression (n = 5066; *P* value≤0.01) and associated at a nominal significance level with Chr9p21 (n = 1698; *P*-value≤0.05) revealed comparable pathways to those identified in *ANRIL* over-expressing cell lines (Table 2). We next investigated adhesion and apoptosis in PBMC from patients, which were either homozygous for the protective or the CAD-risk allele at Chr9p21 (Figures 6A–6B). Consistent with our earlier cell culture studies, the risk allele, which was associated with increased expression of linear *ANRIL* transcripts (Figure 1), led to increased adhesion (*P* = 0.001; Figure 6A) and decreased apoptosis (*P* = 0.001; Figure 6B) compared to PBMCs from carriers of the protective allele.

**Table 2:** Gene set enrichment analysis of *ANRIL*-correlated, Chr9p21-associated genes in 2280 probands of the Leipzig LIFE Heart Study.

Function	*ANRIL* correlated	Ch9p21 associated
Cellular Development	5.24×10^−14^	1.05×10^−03^
Cellular Growth and Proliferation	5.24×10^−14^	3.71×10^−02^
Cell Death/Apoptosis	8.27×10^−14^	4.73×10^−03^
Cell Cycle	4.74×10^−11^	2.21×10^−04^
Gene Expression	2.23×10^−10^	1.64×10^−04^
Cellular Function and Maintenance	9.89×10^−10^	2.21×10^−04^
Cell-To-Cell Signaling and Interaction	1.51×10^−07^	2.21×10^−04^
Post-Translational Modification	4.28×10^−07^	9.75×10^−03^
Cellular Movement/Adhesion	3.70×10^−04^	2.40×10^−04^

Genes correlated with *ANRIL* expression (assays *Ex1-5*, *Ex18-19*; *P*<0.01, n = 5066) and associated with the Chr9p21 genotype (*P*<0.05, n = 1698) in PBMC (n = 2280) of the Leipzig LIFE Heart Study were included in the analysis. *P*-values for enrichment of genes (www.ingenuity.com) are given.

## Discussion

Here we show that the same *ANRIL* isoforms, which were up-regulated in patients carrying the Chr9p21 atherosclerosis risk haplotype, modulate gene networks *in trans* leading to pro-atherogenic cellular properties. At the molecular level, we provide strong evidence for an Alu sequence (Figure 4A) as the key regulatory element responsible for *ANRIL*-mediated *trans*-regulation (Figure 6C). This Alu motif was not only expressed in *ANRIL* RNA transcripts but also marked promoters of target-genes and was associated with epigenetic effector protein binding. Depletion and mutagenesis of the motif reversed *trans*-regulation and normalized cellular functions. More generally, the proposed mechanism of *ANRIL* in atherogenesis highlights a novel role of Alu elements in epigenetic *trans*-regulation of gene networks, which might be relevant for other long ncRNAs as well.

To the best of our knowledge, this is the first study investigating the role of distinct *ANRIL* isoforms in several key mechanisms of atherogenesis using stable over-expression and knock-down approaches (Figure 1 and Figure 2). All investigated *ANRIL* isoforms had more or less pronounced effects on cellular functions (Figure 2, Table S3), but strongest effects were consistently found in cell lines over-expressing *ANRIL* isoforms which were up-regulated in patients carrying the Chr9p21 risk allele (Figure 1). These effects were also dose-dependent (Figure S5). Importantly, results from cell culture studies were validated in primary cells from patients with and without the Chr9p21 risk haplotype (Figure 6). So far, mechanistic work on *ANRIL* has focused on cell proliferation using RNAi approaches [3], [20], [35], [36]. In these studies down-regulation of *ANRIL* led to decreased proliferation in different cell culture models, which is well in line with our observation of increased proliferation in stable *ANRIL* over-expressing cell lines (Figure 2). Network analyses of *trans*-regulated genes in the present study indicated that in addition to proliferation, cell adhesion and apoptosis were also affected in *ANRIL* cell lines. These predictions were functionally validated revealing that *ANRIL* over-expression led to increased adhesion and decreased apoptosis. Again, effects were greatest for those isoforms showing the strongest associations with the Chr9p21 risk genotype and could be reversed by RNAi against *ANRIL* (Figure 2). Moreover, the direction of atherogenic cell functions (increased cell adhesion, increased proliferation and decreased apoptosis) nicely fits with evidence for a potential pro-atherogenic role of *ANRIL* from patient studies [6], [22]. Taken together, our data provide a plausible mechanism for pro-atherogenic functions of Chr9p21-associated *ANRIL* transcripts.

In previous studies, it has been shown that long ncRNA may affect gene expression of target genes [24]–[26], [37]–[39]. Among the best studied examples is the long ncRNA *HOTAIR*, which is transcribed from the *HOXC* cluster and was shown to repress genes in the *HOXD* cluster *in trans* [25], [26]. A potential role of *ANRIL* in *trans*-regulation has previously been postulated by Sato et al, investigating genome-wide mRNA expression in HeLa cells upon transient over-expression of a single *ANRIL* isoform [23]. In the current work, we used a model of stable over-expressing cell lines and found evidence for *trans*-regulation of distinct gene networks that overlapped between different *ANRIL* isoforms (Table 1). To translate these finding to patients with coronary artery disease, we performed pathway analysis of genes that were correlated with *ANRIL* expression and associated with the Chr9p21 risk genotype in 2280 participants of the Leipzig LIFE Heart Study. While no transcript was associated with Chr9p21 at a genome-wide level of significance, we found almost identical pathways in patients with high *ANRIL* expression and high genetic risk at Chr9p21 as in stable *ANRIL* over-expressing cell lines (Table 2). The lack of significant associations of gene expression with Chr9p21 has also been observed by Zeller et al [40] and might be explained by the rather subtle modulation of *ANRIL* by Chr9p21 with slighter effects on *trans*-regulation as opposed to stronger over-expression in the investigated cell culture models. Nevertheless, permanently elevated *ANRIL* levels might activate the observed gene networks leading to subtle changes of cellular functions (Figure 6) and thereby increasing cardiovascular risk over time.

On the mechanistic level, it has been postulated that long ncRNA may serve as a scaffold, guiding effector-proteins to chromatin [24]–[26], [37]–[39]. Indeed, previous work has demonstrated *ANRIL* binding to CBX7 [3] and SUZ12 [20], which are proteins contained in PRC1 and PRC2, respectively. Both previous papers focused on *ANRIL*-mediated *cis*-repression of *CDKN2A* and *CDKN2B* using RNAi but did not investigate potential *trans*-regulatory effects [3], [20]. In the current work, we found no effect of *ANRIL* over-expression on expression of *CDKN2A* and *CDKN2B* (Figure S4). Thus, the spatial coherence of *ANRIL* transcription with adjacent protein-coding genes might be relevant for *cis*-suppression.

This is the first study systematically investigating *ANRIL* binding to 17 different proteins contained in chromatin modifying complexes PRC1, PRC2 and CoREST/Rest (Figure 3). We demonstrated binding to predominantly inhibitory (CBX7, RING1B, EED, JARID2, RBAP46, SUZ12) [41] and potentially activating factors (RYBP, YY1) [29], [30]. CBX7 and SUZ12 were selected as representative proteins for PRC1 and 2, respectively, and were followed up in genome-wide ChIP-seq experiments (Figure 3 and Figure 4). Notably, *ANRIL* over-expression was accompanied with changes of CBX7 and SUZ12 distribution in *ANRIL* target-gene promoters (Figure 3). The role of PRCs in *ANRIL*-mediated gene regulation was further proven by RNAi against CBX7 and SUZ12 which reversed *trans*-regulation of down- and also of up-regulated target genes in *ANRIL* cells (Figure 3G). Induction of gene expression through PcG proteins might seem at odds with the current understanding of PRC-mediated gene silencing. However, binding of PcG proteins in active genes has been described in earlier studies [31]. Moreover, Morey et al demonstrated reduced gene repression in distinct PRC1 compexes containing RYBP, which was shown to associate with *ANRIL* (Figure 3) [42]. Alternatively, *ANRIL*-bound activating factors RYBP and YY1 may directly activate gene expression [29], [30] or *ANRIL* might bind other activating epigenetic effector proteins [43]. Taken together, we demonstrate a central role of PRC1 and PRC2 but not CoREST/Rest in *ANRIL*-mediated repression and induction of genes but additional work is clearly warranted to unravel the complex nature of these *trans*-regulatory mechanisms.

A key limitation in the current understanding of long ncRNA function, in particular with regard to their *trans*-regulatory effects, is the lack of knowledge about specific regulatory sequences or structural motifs. These sequences might be responsible for targeting long ncRNA to distinct regulatory sites in the genome. Here, we provide first evidence for an important role of Alu motifs in this process, which mark the promoters of *ANRIL trans*-regulated genes (Figure 4). Additionally, genome-wide ChIP-seq analysis revealed binding of *ANRIL*-associated PcG effector proteins in close proximity to the Alu motif. Importantly, the same Alu motif was included in *ANRIL* ncRNA transcripts and predicted to be located in a central stem-loop like structure (Figure 4F). Additional evidence for *ANRIL* binding to chromatin comes from its association with histone H3 and H3K27me3 (Figure 4G). In previous work, Alu-like, CG- and GA-rich sequences were proposed as potential RNA-DNA interaction sites associated with RNA:DNA:DNA triplex formation [44], [45]. Paradoxically, Alu occurrence was significantly depleted in *ANRIL*-regulated genes, suggesting that a certain spatial patterning or additional co-factors in promoter regions rather than motif abundance alone might be relevant (Figure 4, Figure 6C). Functional relevance of the motif was proven by depletion and mutagenesis in *ANRIL* RNA, which reversed *trans*-regulation and pro-atherogenic cellular properties (Figure 5). Thus, our data suggest that *ANRIL* may bind to chromatin through interaction via its Alu motif, thereby guiding PRC proteins to *ANRIL*-regulated genes (Figure 6C).

Despite long consideration as “genomic junk”, Alu elements are coming into the focus of intense research. Alu enrichment occurred over the course of evolution [46] and integration at genomic sites was associated with maturation and gain-of-function of ncRNAs [47]. Alu elements as well as distal *ANRIL* exons are not conserved in the orthologous chromosomal region on mouse chromosome 4, which might be an explanation for the lack of effect on atherosclerosis when deleting that region [48]. Recent work by Jeck et al has also demonstrated preferred inclusion of Alu motifs in non-coding RNA lariats which are commonly thought to represent inactive forms of ncRNAs [49]. Whether implementation of Alu motifs in ncRNA lariats leads to silencing of the effector sequences or not remains to be determined.

In summary, our work extends the function of Alu motifs to regulatory components of ncRNAs with a central role in ncRNA-mediated epigenetic *trans*-regulation. Furthermore, it implies a pivotal role for Alu elements in genetically determined vascular disease and describes a plausible molecular mechanism for pro-atherogenic *ANRIL* function at Chr9p21.

## Materials and Methods

### Ethics statement

The Leipzig LIFE Heart Study has been approved by the Ethics Committee of the Medical Faculty of the University Leipzig (Reg. No 276-2005) and was described previously [28]. RNA from peripheral blood mononuclear cells (PBMC; n = 2280) and whole blood (n = 960) from the same patients of this study was isolated as described [6]. Human endarteryectomy specimens (n = 193) were collected in an independent cohort of patients undergoing vascular surgery and the utilization of human vascular tissues was approved by the Ethics Committee of the Medical Faculty Carl Gustav Carus of the Technical University Dresden (EK316122008) [7].

### Genotyping

Genotyping of single nucleotide polymorphisms rs10757274, rs2383206, rs2383297, and rs10757278 in 2280 probands of the Leipzig LIFE Heart Study and in vascular tissue was performed as described [6], [7].

### Cell culture

Initial screening of human cell lines MonoMac, THP1, U937, HEK293, HepG2, CaCo2, and Hutu80 for Chr9p21 gene expression (*MTAP*, *CDKN2A*, *CDKN2B*, *ANRIL*, qRT-PCR assays were described in [6]) revealed that MonoMac and HEK293 cells expressed all Chr9p21 transcripts (data not shown). This suggested that these genes might be functionally relevant in these cells whereas all other investigated cell lines were lacking expression of at least one of the Chr9p21 transcripts. HEK293 cells (DMSZ, ACC305) were cultured in DMEM (Life Technologies) containing 10% fetal calf serum (FCS, Biochrom), 1% penicillin/streptomycin (P/S, Life Technologies). MonoMac cells (DSMZ, ACC124) were cultured in RPMI 1640 (Biochrom) containing 10% FCS, 1% P/S, 1% MEM (Life Technologies), and 1% OPI (Sigma). Cryopreserved PBMC (n = 32) were thawed, cultured in RPMI 1640 (Biochrom) containing 10% FCS, 1% P/S and functional assays were performed within 48 hours.

### Generation of stable cell lines

*ANRIL* isoforms were cloned in the bicistronic pBI-CMV2 (Clontech) or pTRACER-SV40 (Life Technologies) vectors allowing parallel expression of a green fluorescent protein (GFP) and *ANRIL* transcripts. Using Lipofectamine 2000 (Life Technologies), HEK293 cells were either co-transfected with *ANRIL*-pBI-CMV2 vectors/empty vector control and neomycin-encoding vector or transfected with *ANRIL*-pTRACER vectors/empty vector control encoding a GFP-Zeocin resistance gene. Transfected cells were selected with geneticindisulfate (G418, Roth) or Zeocin (Life Technologies). After 2 weeks, stably transfected cells were selected by flow cytometry and over-expression of *ANRIL* was validated by quantitative RT-PCR. On average, 2–4 cell lines were generated per *ANRIL* isoform and vector control, respectively.

### Cell culture functional assays

Cell adhesion assays were performed in 96-well plates coated with collagen (Roche), Matrigel (BD) or PBS. Cells were allowed to adhere for 40 min, quadruplicate measurements/cell line were performed. Numbers of adherent cells were determined using CellTiter-Blue/CellTiter-Glo (Promega) in relation to standard curves of the respective cell line. PBMC adhesion assays were performed accordingly. Quadruplicate measurements/subject were performed. Cellular proliferation was either determined by counting absolute cell numbers (trypan blue staining) or viability assays (CellTiter-Blue/CellTiter-Glo, Promega). Glucose in the cell culture supernatants was determined using standard chemistry (Roche). Apoptosis was determined using flow-cytometric detection of AnnexinV positive cells (GFP-Certified Apoptosis/Necrosis Detection Kit, ENZO), caspase-3 activity (Caspase-3/CPP32 Fluorometric Assay Kit, BioVision; CaspaseGlo 3/7, Promega) and caspase-3 staining.

### RNAi

siRNA knock-down of *ANRIL* (n272158, Life Technologies), CBX7 (s23926, Life Technologies), SUZ12 (s23967, Life Technologies) was performed using Lipofectamine 2000 (Life Technologies). Knock-down efficiency was determined by quantitative RT-PCR and Western blotting.

### Rapid amplification of cDNA ends (RACE) and sequencing of *ANRIL*

10 µg RNA from human PBMC and from MonoMac cells were reverse transcribed using the ExactSTART Eukaryotic mRNA 5′- & 3′-RACE Kit (Epicentre Biotechnologies) according to the manufacturer's instructions. RACE PCR reactions were prepared using Advantage 2 Polymerase Mix (Clontech) and subcloned (pCR2.1-TOPO Vector, Life Technologies). Primers used for RACE experiments are listed in Table S4. Amplification of full-length isoforms was performed using primers listed in Table S5 (schematic in Figure S1). Sequencing of PCR products was performed with an automated DNA sequencer (Applied Biosystems).

### Quantitative RT-PCRs

Quantitative RT-PCRs and analysis of data were performed as described [6]. Primers and probes for *ANRIL* isoforms (n = 5), *TSC22D3*, *COL3A1*, and *U1* are given in Table S6.

### Expression profiling

RNA from cell lines ANRIL1-4 and vector control cell line, siRNA-treated ANRIL2 cells, and RNA from PBMC (n = 2280) was labeled and hybridized to Illumina HumanHT-12 v4 BeadChips. Arrays were scanned with an Illumina iScan microarray scanner. Bead level data preprocessing was done in Illumina GenomeStudio.

### Antibodies

Antibodies against AEBP2 (11232-2-AP, Proteintech), BMI1 (05-637, Millipore), CBX7 (ab21873, Abcam), EED (03-196, Millipore), EZH2 (ACC-3147, Cell Signaling), JARID2 (NB100-2214, Novus Biologicals), MEL18 (ab5267, Abcam), PHF1 (sc-101107, Santa Cruz), PHF19 (11895-1-AP, Proteintech), RBAP46 (MA1-23277, Thermo Scientific), RING1B (NBP1-49966, Novus Biologicals), RYBP (NBP1-97742, Novus Biologicals), SUZ12 (ab12073, Abcam), YY1 (NBP1-46218, Novus Biologicals), LSD1 (39186, Active Motif), REST (07-579, Millipore), CoREST (07-455, Millipore), H3 (ab1791, Abcam), H3K27me3 (ab6002, Abcam), rabbit control IgG (kch-504-250, Diagenode), mouse control IgG (kch-819-015, Diagenode), and goat control IgG (sc-2346, Santa Cruz) were used.

### RNA immunoprecipitation

The immunoprecipitation reaction followed with some modifications previously described protocols [50], [51]: 2×10^7^ ANRIL2, ANRIL4 and vector control cells were treated with 0.1% formaldehyde for 10 min at 23°C. Nuclear extracts were preincubated with non-immune IgG and tRNA (100 µg/ml) for 1 h and primary antibodies and control IgG reacted overnight at 4°C. The immunoprecipitates were washed 3× with low-salt, 3× with high-salt and 1× Li-salt buffers. The retrieved RNA was quantitated, reverse transcribed using random hexamer primers, and analyzed by qRT-PCR with *ANRIL*-specific primers (*Ex1-5* assay, Table S6). *U1*-specific primers were used as negative control (Table S6).

### Chromatin immunoprecipitation and next-generation sequencing

2–5×10^7^ ANRIL2 and vector control cells were fixed with 1% formaldehyde for 10 min at 23°C. Cross-linking reaction was stopped by adding glycine to a final concentration of 0.125 M for 5 min followed by three washes with ice-cold PBS. Cells were harvested, suspended in 50 mM Hepes-KOH, pH 7.5, 100 mM NaCl, 1 mM EDTA, pH 8.0, 0.5 mM EGTA, pH 8.0 and incubated for 10 min at 4°C. Cells were washed with 10 mM Tris-HCl, pH 8.0, 200 mM NaCl, 1 mM EDTA, pH 8.0, 0.5 mM EGTA, pH 8.0 for 10 min, pelleted by centrifugation and the nuclei lysed in 10 mM Tris-HCl, pH 8.0, 200 mM NaCl, 1 mM EDTA, pH 8.0, 0.5 mM EGTA pH 8.0, 0.1% Na-Deoxycholate, 0.5% N-lauroylsarcosine for 10 min at 4°C. Samples were sonicated with Diagenode Bioruptor UCD-300TO (High level, 8×5 cycles 30 sec on/30 sec off, 4°C), centrifuged at 15,000× g for 10 min at 4°C and the supernatant was shock-frozen in liquid nitrogen and stored at −80°C. Nuclear extracts were preadsorbed with non-immune IgG for 1 h and treated with SUZ12, CBX7, and control IgG antibodies overnight at 4°C. Precipitates were processed with the HighCell#ChIP kit (Diagenode) and Illumina DNA sequencing libraries were generated using the ChIP-seq Sample Preparation Kit (IP-102-1001, Illumina). Purity and quantity was measured on an Agilent Technologies 2100 Bioanalyzer. Sequencing was performed with the Illumina Genome Analyzer II platform.

### Bioinformatics and statistical analysis

#### Processing of genome-wide expression data

Illumina HumanHT-12 v4 BeadChips arrays were used for expression profiling. For data from cell lines, bead level data preprocessing was done in Illumina GenomeStudio followed by quantile normalization and background reduction according to standard procedures in the software. Preprocessing of genome-wide expression data in PBMC of the Leipzig LIFE Heart Study comprised selection of expressed features, outlier detection, normalization, and batch correction as described [52], [53].

#### Association analysis

Genotyping quality of SNPs rs10757274, rs2383206, rs2383207, and rs10757278 was determined as described [6] and haplotypes were inferred using fastphase 1.2.9. Associations of haplotypes with expression profiles, and correlation with *ANRIL* (*Ex1-5*, *Ex18-19*) were calculated using robust linear regression models as described (please refer to Supplemental Materials in [6]) with the R software for statistical computing [54].

#### Gene set enrichment analysis

Genome-wide expression profiling in *ANRIL* over-expressing cell lines 1–4 was used to identify transcripts with expression changes of <0.5 and >2 compared to vector control in each cell line. Please refer to legend of Table 1 for absolute numbers of transcripts per analysis. Association of genome-wide expression data with Chr9p21 haplotypes revealed 1698 transcripts with *P*<0.05, correlation of transcript expression with *ANRIL* assays *Ex1-5* and *Ex18-19* revealed 5066 transcripts with *P*<0.01. Gene set enrichment analysis was performed separately for each dataset using the Ingenuity Pathways Analysis (www.ingenuity.com) to identify most significantly enriched biological functions. Comparison of pathways between different analyses was performed according to standard procedures in the software (Table 1, Table 2). Levels of significance were determined using Fisher's exact tests implemented in the software.

#### Comparison of groups

Normality of distribution was tested using the Kolmogorov-Smirnov test implemented in the PRISM statistical software (GraphPad). Comparison of multiple groups was done using ANOVA, and Tukey was performed as post-test. Comparison of 2 groups was done using a t-test.

#### ChIP-seq analysis

ChIP-seq reads were aligned to the *H. sapiens* assembly version hg18 (NCBI36) genome using the *segemehl* aligner [55] with a minimum required accuracy of 85%. Best mapping reads were retained for downstream analysis of SUZ12, CBX7 samples, and input DNA (for read numbers see Table S7) to account for ChIP-seq signals in repetitive regions (Figure 3 and Figure 4). After mapping, the read counts were normalized to the total number of mapped reads in each library, converted to WIG-files and analyzed using sitepro from the CEAS package [56] to test for specific enrichments in vicinity to the previously detected motif. To find signals close to the predicted binding sites, a region of 10,000 nts (–span 10000), a base specific resolution of the resulting plot (–pf-res 1) and the usage of the direction of the motifs (–dir) were set.

#### Motif analysis

The MEME package [57] was used to search for differences in motifs abundance in promoters (5 kb) of differentially regulated genes. To analyze the promoter regions, the width of expected motif was set between 8 and 30 (-minw 8 and –maxw 30), the expected occurrence to ‚zero or one' (-mod zoops) and the maximum number of motifs to search for to 1 (-nmotifs 1). A bootstrapping approach was chosen to overcome computational limitations to test for motif occurrence within promoters of differentially and not-regulated genes. Promoter sequences of up-, down- and not-regulated genes were chopped into pieces of 100 nt. 250 chops of each group were repeatedly sampled and scanned for motif occurrences using the FIMO tool [57] of the MEME package. The process was repeated 1000 times for each of the three groups and a p-value of 1e^−4^ (–output-pthresh 1e-4) was used. Differences in motif occurrence in *ANRIL* up- and down- and not-regulated gene sets were evaluated using the Kolmogorov-Smirnov test provided by the R software for statistical computing [54].

### Accession numbers

ChIP-seq data are deposited in the Sequence Read Archive (SRA) under accession number SRA052089.1. BGO3 (GSM602674) data are publicly available at (http://www.ncbi.nlm.nih.gov/geo).

## Supporting Information

Figure S1 Rapid amplification of cDNA ends (RACE) and PCR experiments. (A) Initially and (B) currently annotated *ANRIL* transcripts and exon labeling. (C) Position of 5′- and 3′-RACE primers. RNA from human peripheral blood mononuclear cells (PBMC) and monocytic cell line MonoMac was used. Exon 7b (22.046.252-22.047.065 bp; NCBI36/hg18), exon 13b (22.077.273-22.077.650 bp;NCBI36/hg18). (D) Positions of PCR primers used for amplification of full-length *ANRIL* isoforms. (E) PCR products and summary of sequencing results of *ANRIL* isoforms (common forward primer in exon 1, reverse primers in exon 7b, 13, 13b, or 20). *ANRIL* isoforms 1–4 showed strongest expression and are highlighted in gel. LM- DNA Molecular Weight Marker X (Roche).
(TIF)

Figure S2 Pearson correlation coefficients for expression levels of different *ANRIL* isoforms in peripheral mononuclear cells of patients of the Leipzig LIFE Heart Study (n = 2280). The following qRT-PCR assays were used: *ANRIL Ex7b* (isoform 1), *Ex7-13* (isoform 2), *Ex10-13b* (isoform 3), *Ex 18-19* (isoform 4), and *Ex1-5* (all isoforms). Coefficients between 0.20 and 0.30 are highlighted in grey, coefficients greater than 0.30 are highlighted in black.
(TIF)

Figure S3 Absolute quantification of *ANRIL* expression in stably over-expressing cell lines ANRIL1-4, vector control, and HEK cells. (A) Graphical summary of exons included in ANRIL1-4 isoforms. *ANRIL* expression levels were determined by qRT-PCR assays (B) *Ex1* (ANRIL1-4), (C) *Ex7b* (ANRIL1), (D) *Ex10-13b* (ANRIL3), (E) *Ex18-19* (ANRIL4). Cell lines lacking over-expression of respective isoform are highlighted in red. 3–4 cell lines/isoform, quadruplicate measurements/cell line. Error bars indicate s.e.m.
(TIF)

Figure S4 mRNA expression of Chr9p21 genes *CDKN2A* and *CDKN2B* in ANRIL1-4 cell lines. mRNA expression of *CDKN2A* transcripts (A) *p16^INK4a^*, (B) *p14^ARF^*, and (C) *CDKN2B* (*p15^INK4b^*) was not significantly altered in cell lines ANRIL1-4 compared to control. Copies of each transcript were normalized to 10^6^ copies of the house-keeping gene beta-actin (*BA*). (A–C) 4 replicates/isoform. Error bars indicate s.e.m.
(TIF)

Figure S5 Dose-dependency of cellular phenotypes in independently established *ANRIL2* and *4* over-expressing cell lines. (A) *ANRIL* expression levels were determined by qRT-PCR assay *Ex1*. ANRIL2 and ANRIL4 cell lines with high (ANRIL2h, ANRIL4h) and low (ANRIL2l, ANRIL4l) expression and vector (v) control. (B) Adhesion, (C) proliferation, and (D) apoptosis in independently established *ANRIL* cell lines. Error bars indicate s.e.m.
(TIF)

Figure S6 siRNA-mediated knockdown of *ANRIL* expression in ANRIL2 and ANRIL4 cells. (A) Corresponds to Figure 2M and O, (B) corresponds to Figure 2N. *ANRIL* expression levels were determined by qRT-PCR assays spanning exon 7–13 (ANRIL2) and exon 6–16 (ANRIL4), respectively, and were normalized to 10^6^ copies of the house-keeping gene *beta-actin* (*BA*). *P*-values for differences of expression compared to SCR (scrambled) control are given. Error bars indicate s.e.m.
(TIF)

Figure S7 CBX7 binding in promoters of *ANRIL trans*-regulated genes in vector control cell line. Up-(green), down-(red), and not (black) regulated transcripts. TSS- transcription start site.
(TIF)

Figure S8 Alu-DEIN core motif in *ANRIL2* and *ANRIL4* RNA transcripts. (A) Sequence of core motif and reverse-complementary motif sequence found in *ANRIL4* and *ANRIL2* transcripts, respectively. One core motif per *ANRIL* isoform was identified. (B) *ANRIL2* isoform with highlighted motif sequence in exon 7. (C) *ANRIL4* isoform with highlighted motif sequence in exons 1/16/18.
(TIF)

Figure S9 Absolute quantification of *ANRIL* expression in stably over-expressing cell lines ANRIL2, 2a–c, ANRIL4, 4a, 4b, ANRIL2*25/*33/*100, vector control, and HEK cells. (A) Graphical summary of exons included in ANRIL2, 2a–c, ANRIL4, 4a, 4b isoforms. *ANRIL* expression levels were determined by qRT-PCR assays (B) *Ex1* and (C) *Ex5-6*. (D) Graphical summary of exons included in ANRIL2 and ANRIL 2 cell lines with 25% (*25), 33% (*33), and 100% (*100) nucleotide exchanges in the 48 base-pair Alu motif (Figure 5G). (E) *ANRIL* expression levels were determined by qRT-PCR assay *Ex1*. Cell lines lacking over-expression of respective isoform are highlighted in red. 2–4 cell lines/isoform, quadruplicate measurements/cell line. Error bars indicate s.e.m.
(TIF)

Table S1 *ANRIL* target-genes with average down-regulation <0.5 fold compared to vector control (n = 219).
(DOC)

Table S2 *ANRIL* target-genes with average up-regulation >2 fold compared to vector control (n = 708).
(DOC)

Table S3 Experimental setup and *P*-values of experiments shown in Figure 2.
(DOC)

Table S4 *ANRIL* RACE primers.
(DOC)

Table S5 Primers used for PCR amplification of *ANRIL* transcripts.
(DOC)

Table S6 Quantitative RT-PCR primers and probes.
(DOC)

Table S7 Mapping of ChIP-seq reads.
(DOC)
