# Supplementary figures and images for: Alu Elements in ANRIL Non-Coding RNA at Chromosome 9p21 Modulate Atherogenic Cell Functions through Trans-Regulation of Gene Networks
Source: PLoS Genet. 2013 Jul 4;9(7):e1003588. doi: 10.1371/journal.pgen.1003588 (PMC3701717; doi:10.1371/journal.pgen.1003588)

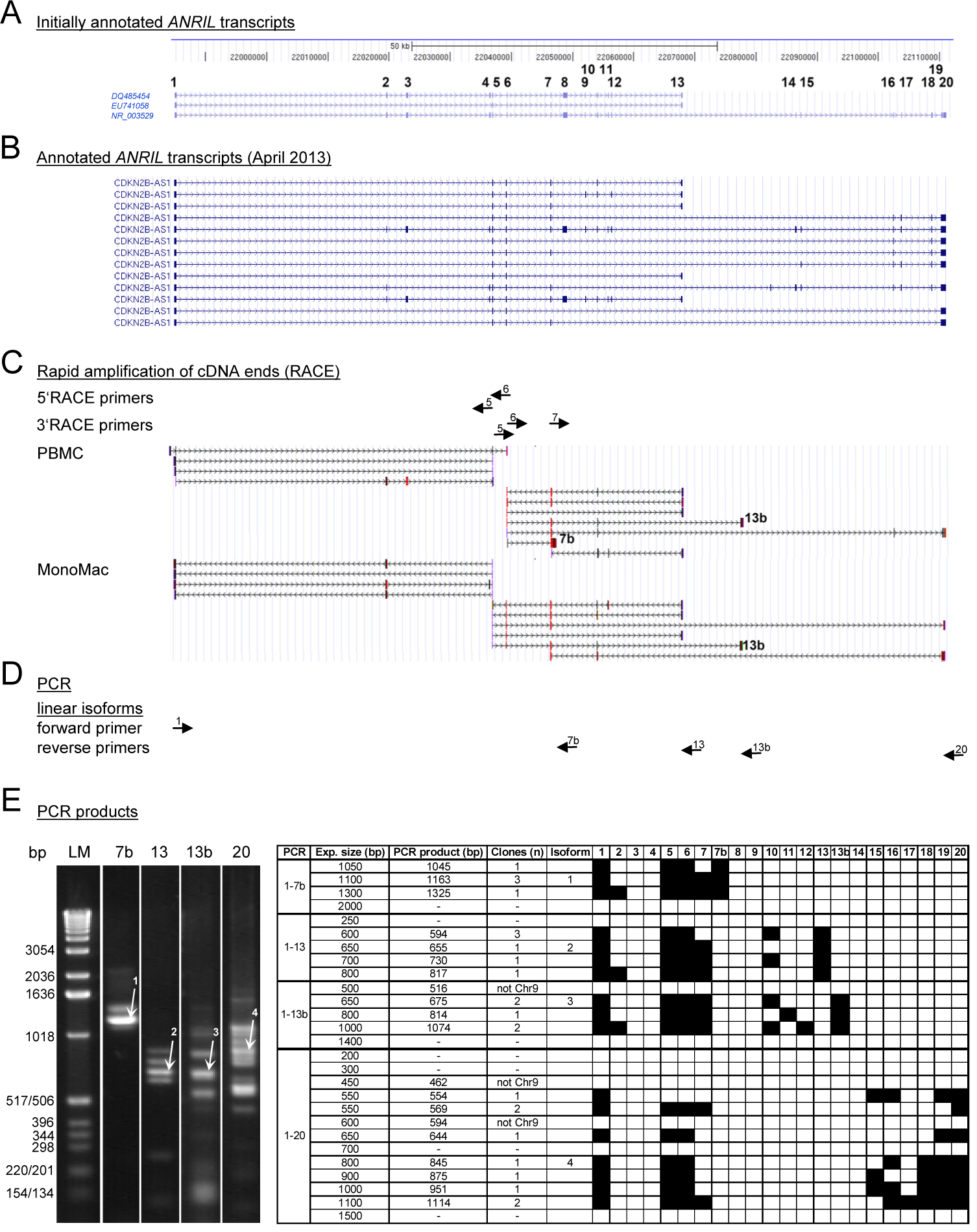

Supplement: Figure S1 — Rapid amplification of cDNA ends (RACE) and PCR experiments. (A) Initially and (B) currently annotated ANRIL transcripts and exon labeling. (C) Position of 5′- and 3′-RACE primers. RNA from human peripheral blood mononuclear cells (PBMC) and monocytic cell line MonoMac was used. Exon 7b (22.046.252-22.047.065 bp; NCBI36/hg18), exon 13b (22.077.273-22.077.650 bp;NCBI36/hg18). (D) Positions of PCR primers used for amplification of full-length ANRIL isoforms. (E) PCR products and summary of sequencing results of ANRIL isoforms (common forward primer in exon 1, reverse primers in exon 7b, 13, 13b, or 20). ANRIL isoforms 1–4 showed strongest expression and are highlighted in gel. LM- DNA Molecular Weight Marker X (Roche). (TIF) [file pgen.1003588.s001.tif]

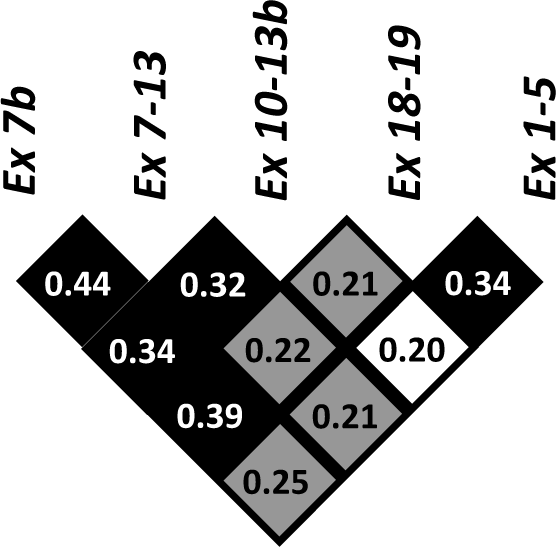

Supplement: Figure S2 — Pearson correlation coefficients for expression levels of different ANRIL isoforms in peripheral mononuclear cells of patients of the Leipzig LIFE Heart Study (n = 2280). The following qRT-PCR assays were used: ANRIL Ex7b (isoform 1), Ex7-13 (isoform 2), Ex10-13b (isoform 3), Ex 18-19 (isoform 4), and Ex1-5 (all isoforms). Coefficients between 0.20 and 0.30 are highlighted in grey, coefficients greater than 0.30 are highlighted in black. (TIF) [file pgen.1003588.s002.tif]

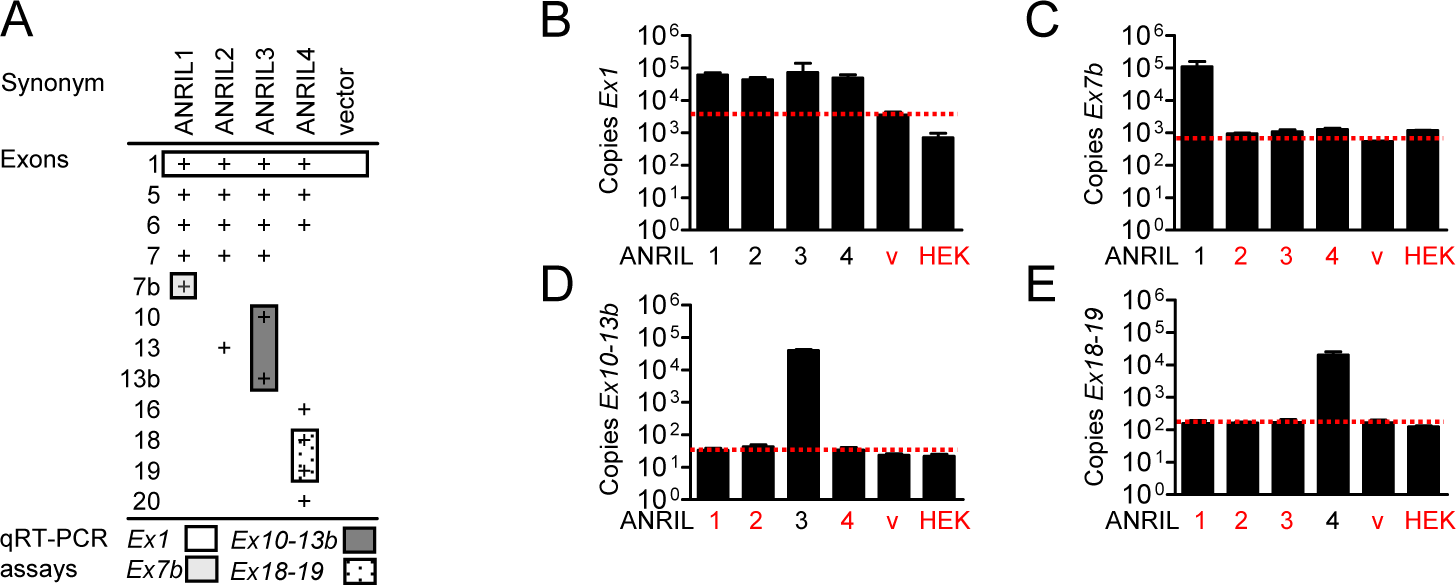

Supplement: Figure S3 — Absolute quantification of ANRIL expression in stably over-expressing cell lines ANRIL1-4, vector control, and HEK cells. (A) Graphical summary of exons included in ANRIL1-4 isoforms. ANRIL expression levels were determined by qRT-PCR assays (B) Ex1 (ANRIL1-4), (C) Ex7b (ANRIL1), (D) Ex10-13b (ANRIL3), (E) Ex18-19 (ANRIL4). Cell lines lacking over-expression of respective isoform are highlighted in red. 3–4 cell lines/isoform, quadruplicate measurements/cell line. Error bars indicate s.e.m. (TIF) [file pgen.1003588.s003.tif]

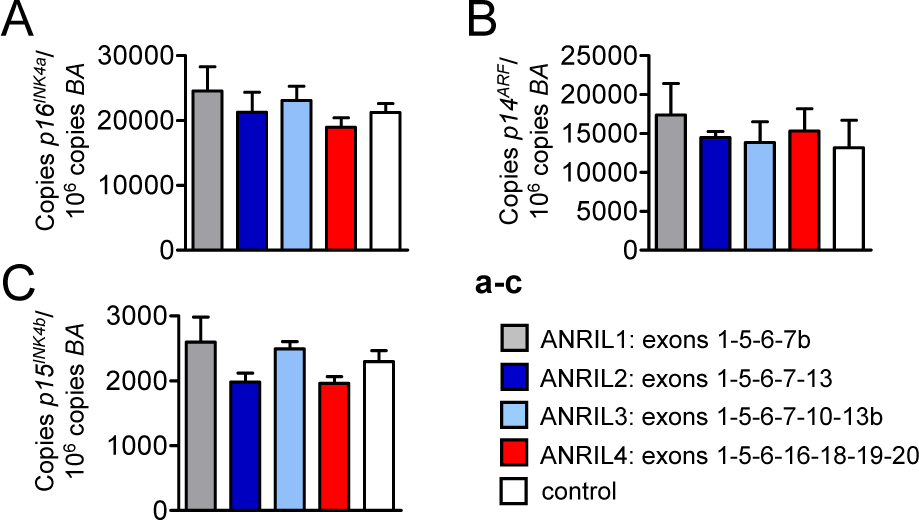

Supplement: Figure S4 — mRNA expression of Chr9p21 genes CDKN2A and CDKN2B in ANRIL1-4 cell lines. mRNA expression of CDKN2A transcripts (A) p16INK4a, (B) p14ARF, and (C) CDKN2B (p15INK4b) was not significantly altered in cell lines ANRIL1-4 compared to control. Copies of each transcript were normalized to 106 copies of the house-keeping gene beta-actin (BA). (A–C) 4 replicates/isoform. Error bars indicate s.e.m. (TIF) [file pgen.1003588.s004.tif]

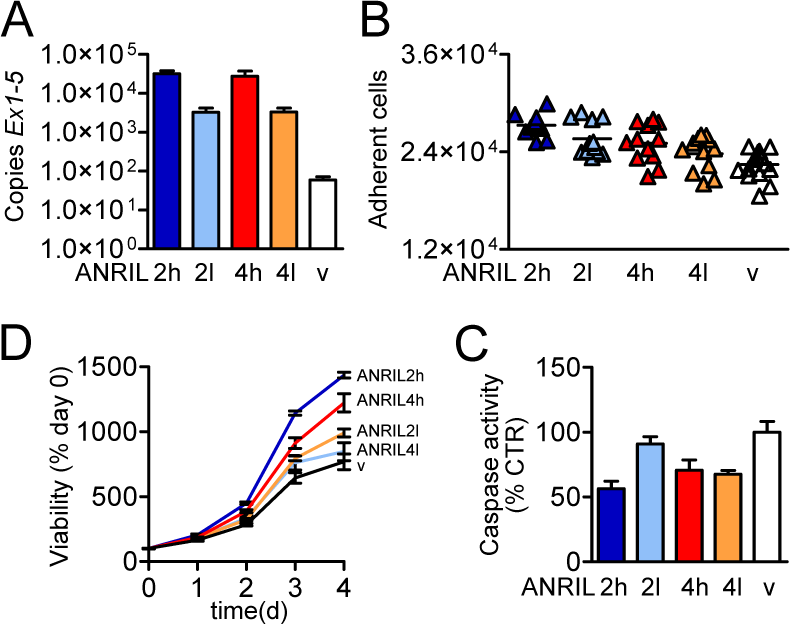

Supplement: Figure S5 — Dose-dependency of cellular phenotypes in independently established ANRIL2 and 4 over-expressing cell lines. (A) ANRIL expression levels were determined by qRT-PCR assay Ex1. ANRIL2 and ANRIL4 cell lines with high (ANRIL2h, ANRIL4h) and low (ANRIL2l, ANRIL4l) expression and vector (v) control. (B) Adhesion, (C) proliferation, and (D) apoptosis in independently established ANRIL cell lines. Error bars indicate s.e.m. (TIF) [file pgen.1003588.s005.tif]

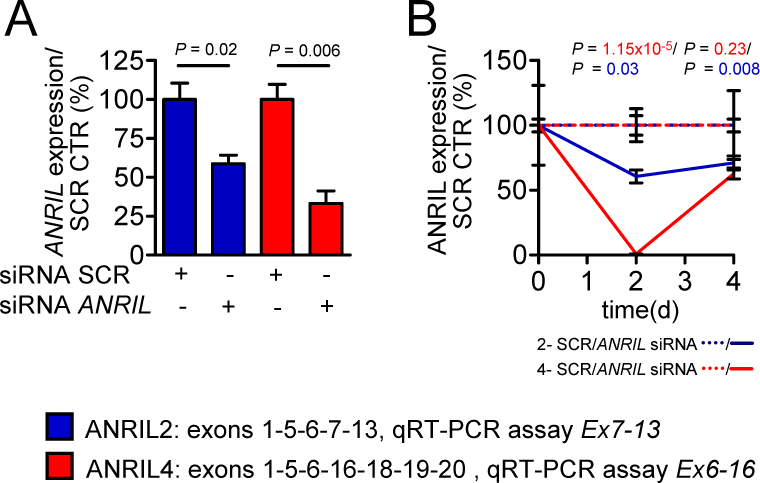

Supplement: Figure S6 — siRNA-mediated knockdown of ANRIL expression in ANRIL2 and ANRIL4 cells. (A) Corresponds to Figure 2M and O, (B) corresponds to Figure 2N. ANRIL expression levels were determined by qRT-PCR assays spanning exon 7–13 (ANRIL2) and exon 6–16 (ANRIL4), respectively, and were normalized to 106 copies of the house-keeping gene beta-actin (BA). P-values for differences of expression compared to SCR (scrambled) control are given. Error bars indicate s.e.m. (TIF) [file pgen.1003588.s006.tif]

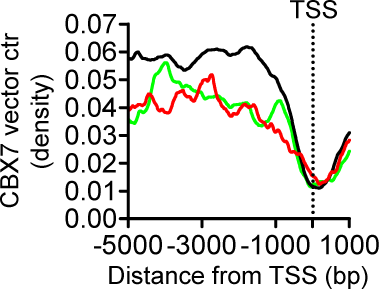

Supplement: Figure S7 — CBX7 binding in promoters of ANRIL trans-regulated genes in vector control cell line. Up-(green), down-(red), and not (black) regulated transcripts. TSS- transcription start site. (TIF) [file pgen.1003588.s007.tif]

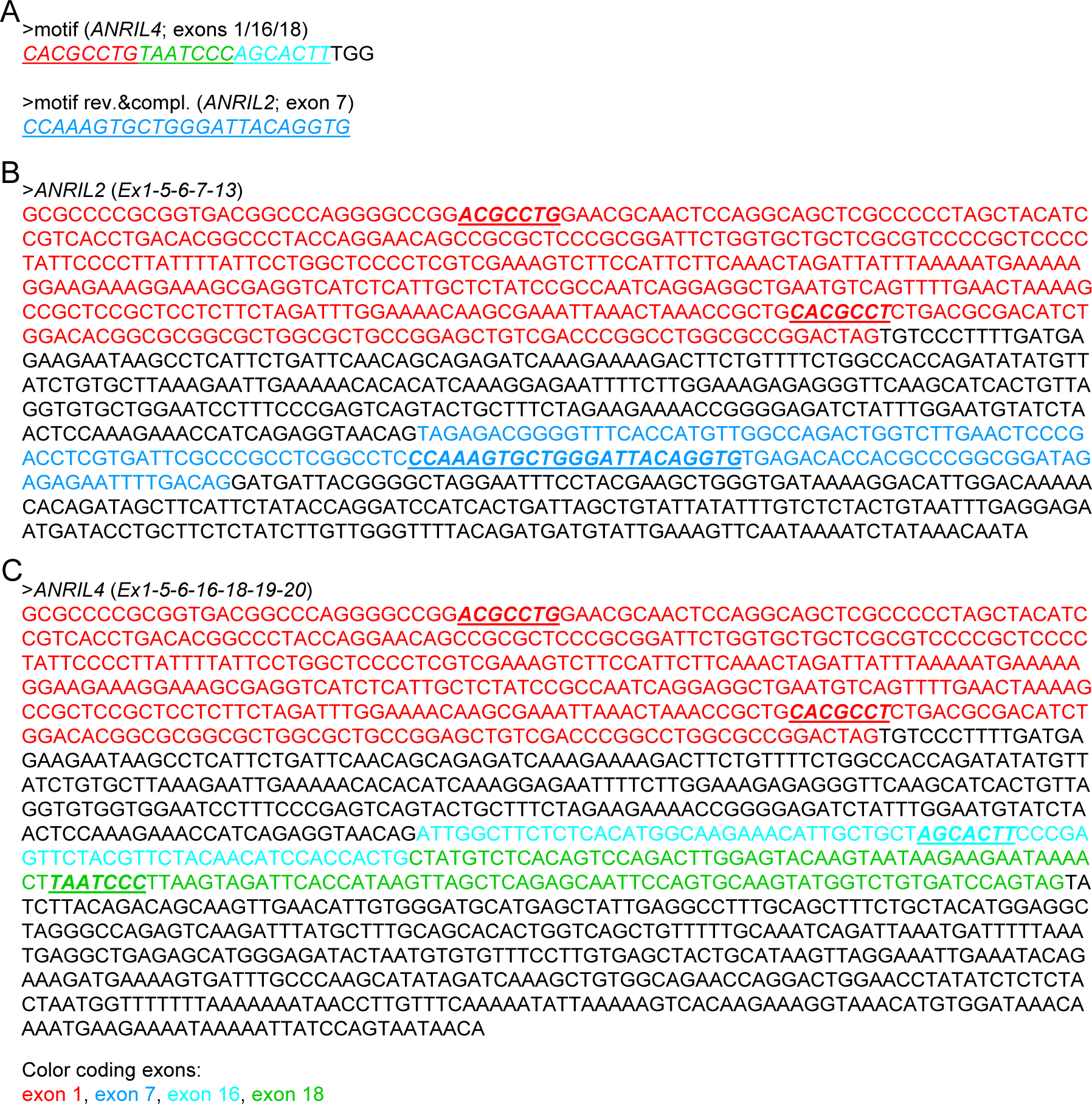

Supplement: Figure S8 — Alu-DEIN core motif in ANRIL2 and ANRIL4 RNA transcripts. (A) Sequence of core motif and reverse-complementary motif sequence found in ANRIL4 and ANRIL2 transcripts, respectively. One core motif per ANRIL isoform was identified. (B) ANRIL2 isoform with highlighted motif sequence in exon 7. (C) ANRIL4 isoform with highlighted motif sequence in exons 1/16/18. (TIF) [file pgen.1003588.s008.tif]

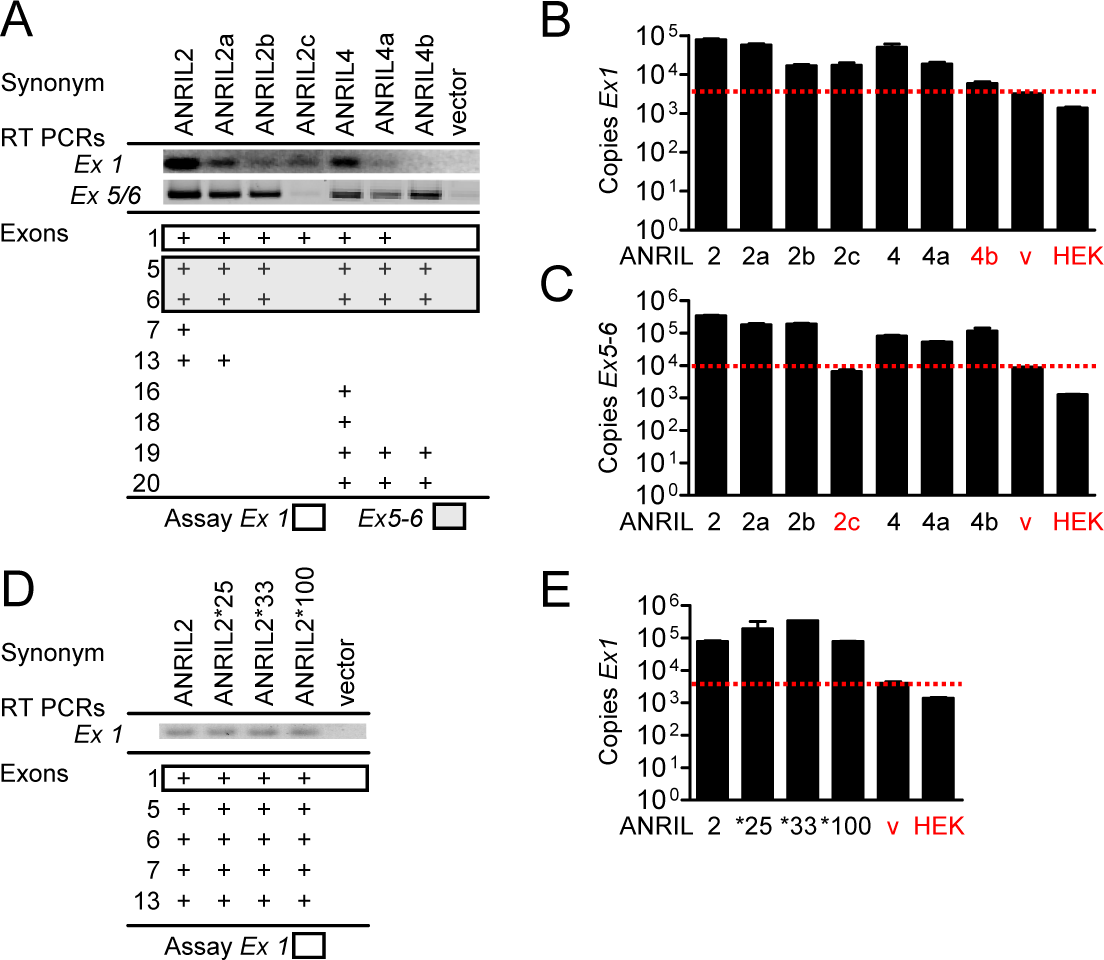

Supplement: Figure S9 — Absolute quantification of ANRIL expression in stably over-expressing cell lines ANRIL2, 2a–c, ANRIL4, 4a, 4b, ANRIL2*25/*33/*100, vector control, and HEK cells. (A) Graphical summary of exons included in ANRIL2, 2a–c, ANRIL4, 4a, 4b isoforms. ANRIL expression levels were determined by qRT-PCR assays (B) Ex1 and (C) Ex5-6. (D) Graphical summary of exons included in ANRIL2 and ANRIL 2 cell lines with 25% (*25), 33% (*33), and 100% (*100) nucleotide exchanges in the 48 base-pair Alu motif (Figure 5G). (E) ANRIL expression levels were determined by qRT-PCR assay Ex1. Cell lines lacking over-expression of respective isoform are highlighted in red. 2–4 cell lines/isoform, quadruplicate measurements/cell line. Error bars indicate s.e.m. (TIF) [file pgen.1003588.s009.tif]
