# Supplementary material for: Alu Elements in ANRIL Non-Coding RNA at Chromosome 9p21 Modulate Atherogenic Cell Functions through Trans-Regulation of Gene Networks
Source: PLoS Genet. 2013 Jul 4;9(7):e1003588. doi: 10.1371/journal.pgen.1003588 (PMC3701717; doi:10.1371/journal.pgen.1003588)
Supplement: Table S1 — ANRIL target-genes with average down-regulation <0.5 fold compared to vector control (n = 219). (DOC) [file pgen.1003588.s010.doc]

**Table S1. *ANRIL* target-genes with average down-regulation < 0.5 fold compared to vector control (n = 219).**

|  |  | Fold change of expression compared to vector control  sorted by average expression change | | | | |
| --- | --- | --- | --- | --- | --- | --- |
| Probe-ID | Symbol | ANRIL 1 | ANRIL 2 | ANRIL 3 | ANRIL 4 | ANRIL avg |
| ILMN_1688335 | MAGEB2 | 0.31 | 0.02 | 0.02 | 0.02 | 0.09 |
| ILMN_1792978 | HAS2 | 0.06 | 0.02 | 0.49 | 0.02 | 0.15 |
| ILMN_1801616 | EMP1 | 0.33 | 0.03 | 0.39 | 0.05 | 0.20 |
| ILMN_1773079 | COL3A1 | 0.32 | 0.06 | 0.32 | 0.11 | 0.20 |
| ILMN_2384857 | DHRS2 | 0.47 | 0.06 | 0.30 | 0.10 | 0.23 |
| ILMN_1670008 | FLCN | 0.22 | 0.20 | 0.29 | 0.20 | 0.23 |
| ILMN_1780911 | MAGEB6B | 0.37 | 0.18 | 0.18 | 0.18 | 0.23 |
| ILMN_1697499 | HLA-DRB5 | 0.13 | 0.13 | 0.13 | 0.57 | 0.24 |
| ILMN_2120210 | RCAN2 | 0.26 | 0.25 | 0.31 | 0.14 | 0.24 |
| ILMN_1725726 | DHRS2 | 0.46 | 0.08 | 0.28 | 0.17 | 0.25 |
| ILMN_1740426 | RASD1 | 0.23 | 0.27 | 0.30 | 0.23 | 0.26 |
| ILMN_1678842 | THBS2 | 0.26 | 0.26 | 0.30 | 0.26 | 0.27 |
| ILMN_1788793 | KLF8 | 0.12 | 0.12 | 0.71 | 0.12 | 0.27 |
| ILMN_1730645 | TMEFF2 | 0.46 | 0.11 | 0.42 | 0.11 | 0.27 |
| ILMN_3248910 | MIR155HG | 0.28 | 0.28 | 0.28 | 0.28 | 0.28 |
| ILMN_1753525 | TCEAL7 | 0.28 | 0.24 | 0.37 | 0.23 | 0.28 |
| ILMN_1801454 | GJB7 | 0.32 | 0.29 | 0.29 | 0.29 | 0.30 |
| ILMN_3238173 | FAM90A7 | 0.37 | 0.27 | 0.27 | 0.27 | 0.30 |
| ILMN_1693397 | PSG4 | 0.36 | 0.17 | 0.47 | 0.24 | 0.31 |
| ILMN_2124241 | MUM1L1 | 0.58 | 0.16 | 0.28 | 0.22 | 0.31 |
| ILMN_1797903 | ZNF544 | 0.23 | 0.06 | 0.91 | 0.04 | 0.31 |
| ILMN_1685641 | BCHE | 0.39 | 0.07 | 0.72 | 0.08 | 0.31 |
| ILMN_1705224 | TMEM110 | 0.25 | 0.25 | 0.25 | 0.50 | 0.31 |
| ILMN_3251572 | FAM134A | 0.48 | 0.27 | 0.26 | 0.31 | 0.33 |
| ILMN_1680344 | MYOM1 | 0.34 | 0.37 | 0.34 | 0.34 | 0.34 |
| ILMN_1858762 | | 0.46 | 0.31 | 0.31 | 0.31 | 0.34 |
| ILMN_1721559 | FABP6 | 0.39 | 0.33 | 0.33 | 0.33 | 0.35 |
| ILMN_1736929 | PLB1 | 0.51 | 0.31 | 0.29 | 0.29 | 0.35 |
| ILMN_1810289 | FER1L3 | 0.35 | 0.35 | 0.35 | 0.35 | 0.35 |
| ILMN_1718520 | C10orf59 | 0.38 | 0.21 | 0.34 | 0.48 | 0.35 |
| ILMN_1771393 | C3orf23 | 0.40 | 0.33 | 0.35 | 0.33 | 0.35 |
| ILMN_1699365 | SLC30A6 | 0.47 | 0.41 | 0.18 | 0.34 | 0.35 |
| ILMN_2166716 | VCX-C | 0.62 | 0.21 | 0.36 | 0.21 | 0.35 |
| ILMN_2384745 | PSG4 | 0.33 | 0.20 | 0.43 | 0.46 | 0.36 |
| ILMN_3229749 | TRIM53 | 0.64 | 0.25 | 0.29 | 0.25 | 0.36 |
| ILMN_1668442 | C21orf42 | 0.41 | 0.34 | 0.34 | 0.36 | 0.36 |
| ILMN_1859036 | | 0.39 | 0.31 | 0.45 | 0.31 | 0.36 |
| ILMN_1684886 | VCX | 0.39 | 0.35 | 0.35 | 0.35 | 0.36 |
| ILMN_1793012 | FLJ10803 | 0.54 | 0.29 | 0.29 | 0.34 | 0.37 |
| ILMN_2176592 | BCHE | 0.50 | 0.02 | 0.80 | 0.15 | 0.37 |
| ILMN_3296181 | LOC100131894 | 0.35 | 0.38 | 0.39 | 0.35 | 0.37 |
| ILMN_1657039 | FILIP1 | 0.53 | 0.12 | 0.56 | 0.26 | 0.37 |
| ILMN_1681332 | LOC646496 | 0.45 | 0.38 | 0.32 | 0.32 | 0.37 |
| ILMN_1863401 | | 0.31 | 0.31 | 0.43 | 0.43 | 0.37 |
| ILMN_3238757 | CCDC152 | 0.53 | 0.44 | 0.26 | 0.26 | 0.37 |
| ILMN_1663916 | ARHGAP9 | 0.35 | 0.42 | 0.37 | 0.35 | 0.37 |
| ILMN_3307724 | ZNF134 | 0.32 | 0.56 | 0.35 | 0.26 | 0.37 |
| ILMN_1707551 | AFMID | 0.58 | 0.35 | 0.30 | 0.26 | 0.37 |
| ILMN_1807882 | LOC644646 | 0.44 | 0.25 | 0.59 | 0.25 | 0.38 |
| ILMN_3285621 | LOC441481 | 0.43 | 0.25 | 0.59 | 0.25 | 0.38 |
| ILMN_2367883 | GEM | 0.49 | 0.49 | 0.27 | 0.27 | 0.38 |
| ILMN_1791721 | KRTAP21-2 | 0.38 | 0.38 | 0.38 | 0.38 | 0.38 |
| ILMN_1658356 | PAMR1 | 0.58 | 0.60 | 0.22 | 0.13 | 0.38 |
| ILMN_1682599 | GPRC5A | 0.57 | 0.32 | 0.32 | 0.32 | 0.38 |
| ILMN_1684485 | LOC650363 | 0.46 | 0.32 | 0.47 | 0.29 | 0.39 |
| ILMN_2222786 | ZNF192 | 0.40 | 0.45 | 0.35 | 0.35 | 0.39 |
| ILMN_1749368 | HIST1H3H | 0.26 | 0.30 | 0.67 | 0.32 | 0.39 |
| ILMN_2290338 | RCAN1 | 0.42 | 0.38 | 0.38 | 0.38 | 0.39 |
| ILMN_1802094 | ZNF280A | 0.56 | 0.21 | 0.58 | 0.21 | 0.39 |
| ILMN_2141482 | SERPINF1 | 0.40 | 0.37 | 0.62 | 0.17 | 0.39 |
| ILMN_2215989 | NEFM | 0.72 | 0.00 | 0.73 | 0.12 | 0.39 |
| ILMN_3194480 | DUB4 | 0.71 | 0.33 | 0.27 | 0.26 | 0.39 |
| ILMN_1706969 | BEND6 | 0.65 | 0.31 | 0.31 | 0.31 | 0.39 |
| ILMN_1679984 | ZCCHC12 | 0.35 | 0.31 | 0.37 | 0.55 | 0.40 |
| ILMN_2070477 | TAF8 | 0.67 | 0.54 | 0.18 | 0.20 | 0.40 |
| ILMN_3200430 | LOC644667 | 0.45 | 0.37 | 0.37 | 0.39 | 0.40 |
| ILMN_1740650 | KCNQ4 | 0.37 | 0.43 | 0.37 | 0.43 | 0.40 |
| ILMN_1749070 | HLA-DPB1 | 0.34 | 0.57 | 0.34 | 0.34 | 0.40 |
| ILMN_2381257 | DSC2 | 0.40 | 0.53 | 0.45 | 0.22 | 0.40 |
| ILMN_1812795 | RUNX1T1 | 0.21 | 0.58 | 0.59 | 0.21 | 0.40 |
| ILMN_3244903 | C7orf65 | 0.40 | 0.40 | 0.40 | 0.40 | 0.40 |
| ILMN_1719298 | OR51E2 | 0.34 | 0.40 | 0.53 | 0.33 | 0.40 |
| ILMN_1809817 | FLJ23834 | 0.40 | 0.40 | 0.40 | 0.40 | 0.40 |
| ILMN_1702363 | SULF1 | 0.22 | 0.39 | 0.76 | 0.25 | 0.40 |
| ILMN_1745583 | EXOC1 | 0.41 | 0.41 | 0.41 | 0.41 | 0.41 |
| ILMN_1845347 | | 0.40 | 0.40 | 0.40 | 0.43 | 0.41 |
| ILMN_1852022 | KIAA1881 | 0.35 | 0.32 | 0.26 | 0.71 | 0.41 |
| ILMN_3299356 | LOC729004 | 0.71 | 0.37 | 0.35 | 0.20 | 0.41 |
| ILMN_3237089 | LOC728393 | 0.76 | 0.31 | 0.33 | 0.23 | 0.41 |
| ILMN_3245716 | LOC647334 | 0.84 | 0.08 | 0.64 | 0.08 | 0.41 |
| ILMN_1787186 | NOV | 0.59 | 0.01 | 0.94 | 0.10 | 0.41 |
| ILMN_1679780 | LOC145814 | 0.55 | 0.36 | 0.36 | 0.36 | 0.41 |
| ILMN_1772218 | HLA-DPA1 | 0.41 | 0.26 | 0.46 | 0.51 | 0.41 |
| ILMN_1723912 | IFI44L | 0.45 | 0.20 | 0.81 | 0.20 | 0.41 |
| ILMN_2072622 | FLJ12684 | 0.39 | 0.39 | 0.39 | 0.46 | 0.41 |
| ILMN_1787266 | SPINK1 | 0.84 | 0.27 | 0.27 | 0.27 | 0.41 |
| ILMN_1749044 | PVRL4 | 0.84 | 0.17 | 0.47 | 0.17 | 0.41 |
| ILMN_1781626 | C1S | 0.48 | 0.35 | 0.45 | 0.38 | 0.41 |
| ILMN_3215886 | LOC100132972 | 0.59 | 0.36 | 0.36 | 0.36 | 0.42 |
| ILMN_1706845 | OPHN1 | 0.48 | 0.25 | 0.56 | 0.38 | 0.42 |
| ILMN_1683067 | ELSPBP1 | 0.51 | 0.39 | 0.39 | 0.39 | 0.42 |
| ILMN_1813379 | TNFRSF9 | 0.41 | 0.44 | 0.41 | 0.41 | 0.42 |
| ILMN_1814200 | BMP2K | 0.60 | 0.31 | 0.39 | 0.38 | 0.42 |
| ILMN_1721770 | PAPPA | 0.29 | 0.16 | 0.85 | 0.37 | 0.42 |
| ILMN_2184373 | IL8 | 0.58 | 0.46 | 0.29 | 0.34 | 0.42 |
| ILMN_1671557 | PHLDA2 | 0.60 | 0.11 | 0.88 | 0.11 | 0.42 |
| ILMN_1790843 | NLRP11 | 0.71 | 0.33 | 0.33 | 0.33 | 0.42 |
| ILMN_1654721 | LOC645836 | 0.78 | 0.35 | 0.32 | 0.24 | 0.42 |
| ILMN_3282786 | LOC649346 | 0.71 | 0.15 | 0.66 | 0.18 | 0.42 |
| ILMN_2142752 | MANSC1 | 0.29 | 0.87 | 0.15 | 0.39 | 0.42 |
| ILMN_3237246 | GGT8P | 0.39 | 0.50 | 0.40 | 0.41 | 0.42 |
| ILMN_2251784 | NSF | 0.33 | 0.47 | 0.24 | 0.66 | 0.42 |
| ILMN_1699961 | LOC645402 | 0.63 | 0.31 | 0.44 | 0.31 | 0.43 |
| ILMN_2335304 | WDR35 | 0.40 | 0.51 | 0.40 | 0.40 | 0.43 |
| ILMN_1689212 | RSHL3 | 0.56 | 0.20 | 0.45 | 0.49 | 0.43 |
| ILMN_1674967 | TSGA10 | 0.56 | 0.30 | 0.55 | 0.29 | 0.43 |
| ILMN_3238233 | HIST2H4B | 0.44 | 0.44 | 0.34 | 0.49 | 0.43 |
| ILMN_3247297 | LOC100132541 | 0.69 | 0.49 | 0.34 | 0.20 | 0.43 |
| ILMN_3245143 | CCDC19 | 0.28 | 0.52 | 0.24 | 0.67 | 0.43 |
| ILMN_3255679 | LOC100128193 | 0.33 | 0.59 | 0.46 | 0.34 | 0.43 |
| ILMN_2349061 | IRF7 | 0.97 | 0.20 | 0.37 | 0.20 | 0.43 |
| ILMN_1748092 | F8A3 | 0.56 | 0.57 | 0.30 | 0.30 | 0.43 |
| ILMN_3240058 | LOC100134102 | 0.68 | 0.42 | 0.31 | 0.31 | 0.43 |
| ILMN_1839781 | | 0.57 | 0.52 | 0.32 | 0.32 | 0.43 |
| ILMN_1713818 | LOC647415 | 0.52 | 0.51 | 0.35 | 0.35 | 0.43 |
| ILMN_2408851 | ARHGAP30 | 0.31 | 0.31 | 0.79 | 0.31 | 0.43 |
| ILMN_2326793 | NUP98 | 0.77 | 0.41 | 0.22 | 0.35 | 0.44 |
| ILMN_1719759 | TNC | 0.61 | 0.08 | 0.98 | 0.08 | 0.44 |
| ILMN_1682120 | THEM4 | 0.87 | 0.31 | 0.32 | 0.24 | 0.44 |
| ILMN_2268593 | C11orf63 | 0.47 | 0.52 | 0.35 | 0.41 | 0.44 |
| ILMN_1714228 | SPTBN1 | 0.38 | 0.38 | 0.61 | 0.38 | 0.44 |
| ILMN_1838830 | | 0.41 | 0.47 | 0.41 | 0.47 | 0.44 |
| ILMN_3238339 | LOC100132770 | 0.55 | 0.65 | 0.30 | 0.25 | 0.44 |
| ILMN_2366967 | WT1 | 0.51 | 0.46 | 0.24 | 0.54 | 0.44 |
| ILMN_1853093 | | 0.48 | 0.32 | 0.37 | 0.59 | 0.44 |
| ILMN_1769615 | FLRT2 | 0.71 | 0.06 | 0.86 | 0.13 | 0.44 |
| ILMN_1812070 | ABCB1 | 0.57 | 0.09 | 0.65 | 0.46 | 0.44 |
| ILMN_1752455 | DOCK5 | 0.42 | 0.42 | 0.51 | 0.42 | 0.44 |
| ILMN_1758067 | RGS4 | 0.40 | 0.29 | 0.36 | 0.73 | 0.44 |
| ILMN_1769453 | PIH1D2 | 0.30 | 0.30 | 0.89 | 0.30 | 0.44 |
| ILMN_1747412 | DPP3 | 0.81 | 0.49 | 0.28 | 0.21 | 0.44 |
| ILMN_1771286 | LOC653513 | 0.35 | 0.26 | 0.91 | 0.26 | 0.45 |
| ILMN_3188357 | LOC100129559 | 0.61 | 0.39 | 0.39 | 0.39 | 0.45 |
| ILMN_1744212 | INPP5D | 0.51 | 0.12 | 0.69 | 0.47 | 0.45 |
| ILMN_2083559 | KIAA0415 | 0.82 | 0.32 | 0.32 | 0.32 | 0.45 |
| ILMN_3249667 | LOC100133678 | 0.40 | 0.60 | 0.40 | 0.40 | 0.45 |
| ILMN_2347145 | DCN | 0.42 | 0.42 | 0.54 | 0.42 | 0.45 |
| ILMN_1662370 | LOC646906 | 0.78 | 0.37 | 0.32 | 0.32 | 0.45 |
| ILMN_1915432 | | 0.37 | 0.43 | 0.44 | 0.56 | 0.45 |
| ILMN_1832047 | | 0.58 | 0.35 | 0.52 | 0.35 | 0.45 |
| ILMN_1675844 | WDR1 | 0.73 | 0.45 | 0.36 | 0.26 | 0.45 |
| ILMN_1871124 | | 0.38 | 0.38 | 0.50 | 0.55 | 0.45 |
| ILMN_3251283 | HDAC2 | 0.65 | 0.39 | 0.46 | 0.31 | 0.45 |
| ILMN_3246839 | LOC100132769 | 0.52 | 0.49 | 0.40 | 0.40 | 0.45 |
| ILMN_1728768 | LOC643420 | 0.76 | 0.35 | 0.35 | 0.35 | 0.45 |
| ILMN_1791270 | CDH10 | 0.38 | 0.27 | 0.87 | 0.29 | 0.45 |
| ILMN_1748970 | PRR15L | 0.60 | 0.53 | 0.34 | 0.34 | 0.45 |
| ILMN_1724054 | ZNF519 | 0.64 | 0.28 | 0.64 | 0.27 | 0.46 |
| ILMN_1659255 | RP2 | 0.61 | 0.54 | 0.20 | 0.47 | 0.46 |
| ILMN_1664772 | ATP2B4 | 0.44 | 0.33 | 0.70 | 0.36 | 0.46 |
| ILMN_2156786 | PGGT1B | 0.37 | 0.49 | 0.40 | 0.57 | 0.46 |
| ILMN_1698259 | TMEM100 | 0.31 | 0.45 | 0.47 | 0.61 | 0.46 |
| ILMN_1679267 | TGM2 | 0.79 | 0.48 | 0.28 | 0.28 | 0.46 |
| ILMN_1799151 | PPIL4 | 0.72 | 0.30 | 0.38 | 0.43 | 0.46 |
| ILMN_1792733 | FOXA3 | 0.80 | 0.44 | 0.36 | 0.24 | 0.46 |
| ILMN_1775224 | NOS3 | 0.84 | 0.33 | 0.33 | 0.33 | 0.46 |
| ILMN_1727065 | FLJ42289 | 0.35 | 0.50 | 0.46 | 0.54 | 0.46 |
| ILMN_2101410 | ZNF625 | 0.62 | 0.37 | 0.48 | 0.37 | 0.46 |
| ILMN_3240168 | C3orf74 | 0.46 | 0.53 | 0.30 | 0.56 | 0.46 |
| ILMN_1837082 | | 0.36 | 0.59 | 0.39 | 0.50 | 0.46 |
| ILMN_3251638 | TRIM49 | 0.58 | 0.31 | 0.64 | 0.31 | 0.46 |
| ILMN_3212962 | CLDN24 | 0.59 | 0.42 | 0.42 | 0.42 | 0.46 |
| ILMN_1810942 | CYP3A5 | 0.60 | 0.07 | 1.04 | 0.14 | 0.46 |
| ILMN_1682761 | C17orf87 | 0.37 | 0.49 | 0.48 | 0.51 | 0.46 |
| ILMN_1662880 | FIS | 0.36 | 0.35 | 0.44 | 0.71 | 0.46 |
| ILMN_2220729 | CLEC3A | 0.72 | 0.11 | 0.83 | 0.21 | 0.47 |
| ILMN_2349138 | CDC42SE1 | 0.56 | 0.49 | 0.30 | 0.51 | 0.47 |
| ILMN_1700428 | HLA-DOB | 0.54 | 0.19 | 0.60 | 0.54 | 0.47 |
| ILMN_1709153 | PRR16 | 0.97 | 0.13 | 0.64 | 0.13 | 0.47 |
| ILMN_1753122 | MCPH1 | 0.87 | 0.31 | 0.42 | 0.29 | 0.47 |
| ILMN_2094875 | ABCB1 | 0.69 | 0.05 | 0.73 | 0.42 | 0.47 |
| ILMN_1704236 | MAX | 0.46 | 0.62 | 0.47 | 0.34 | 0.47 |
| ILMN_1814305 | SAMD9 | 0.68 | 0.09 | 0.70 | 0.41 | 0.47 |
| ILMN_1792283 | RPP30 | 0.77 | 0.37 | 0.38 | 0.37 | 0.47 |
| ILMN_2184184 | ANXA1 | 0.60 | 0.14 | 0.87 | 0.28 | 0.47 |
| ILMN_1809511 | GRINL1A | 0.84 | 0.44 | 0.31 | 0.31 | 0.47 |
| ILMN_3263451 | LOC100130344 | 0.69 | 0.40 | 0.40 | 0.40 | 0.47 |
| ILMN_1708126 | LOC653643 | 0.36 | 0.65 | 0.43 | 0.45 | 0.47 |
| ILMN_1678906 | LOC644243 | 0.64 | 0.42 | 0.42 | 0.42 | 0.48 |
| ILMN_1759984 | KCTD1 | 0.62 | 0.46 | 0.41 | 0.41 | 0.48 |
| ILMN_1802457 | MAX | 0.68 | 0.41 | 0.68 | 0.14 | 0.48 |
| ILMN_1899601 | | 0.50 | 0.38 | 0.64 | 0.38 | 0.48 |
| ILMN_2274586 | PKD1L2 | 0.39 | 0.58 | 0.56 | 0.39 | 0.48 |
| ILMN_1892511 | | 0.37 | 0.64 | 0.53 | 0.37 | 0.48 |
| ILMN_1829030 | | 0.51 | 0.59 | 0.52 | 0.30 | 0.48 |
| ILMN_1733415 | MFAP5 | 0.36 | 0.54 | 0.51 | 0.50 | 0.48 |
| ILMN_1883404 | | 0.69 | 0.54 | 0.39 | 0.30 | 0.48 |
| ILMN_1769032 | GLYATL1 | 0.40 | 0.55 | 0.40 | 0.58 | 0.48 |
| ILMN_1716790 | APBB2 | 0.62 | 0.56 | 0.49 | 0.26 | 0.48 |
| ILMN_1660176 | ZNF582 | 0.74 | 0.31 | 0.56 | 0.31 | 0.48 |
| ILMN_3280459 | LOC100131686 | 0.44 | 0.69 | 0.35 | 0.45 | 0.48 |
| ILMN_1700981 | ST7OT2 | 0.40 | 0.67 | 0.45 | 0.40 | 0.48 |
| ILMN_1804629 | TPK1 | 0.53 | 0.44 | 0.20 | 0.75 | 0.48 |
| ILMN_3189715 | HDAC7 | 0.78 | 0.60 | 0.28 | 0.28 | 0.48 |
| ILMN_2408877 | MLL3 | 0.49 | 0.42 | 0.55 | 0.48 | 0.48 |
| ILMN_1769876 | TBC1D2 | 0.79 | 0.42 | 0.43 | 0.30 | 0.48 |
| ILMN_2190414 | ZNF83 | 0.76 | 0.03 | 1.12 | 0.03 | 0.48 |
| ILMN_2319544 | CAMK2D | 0.61 | 0.77 | 0.25 | 0.30 | 0.48 |
| ILMN_1704238 | C14orf126 | 0.75 | 0.39 | 0.47 | 0.32 | 0.48 |
| ILMN_2362232 | ZNF331 | 0.57 | 0.30 | 0.94 | 0.13 | 0.49 |
| ILMN_1762284 | ASPRV1 | 0.77 | 0.32 | 0.54 | 0.30 | 0.49 |
| ILMN_1701482 | POFUT2 | 0.62 | 0.36 | 0.36 | 0.60 | 0.49 |
| ILMN_1655809 | SF3A1 | 0.57 | 0.60 | 0.39 | 0.38 | 0.49 |
| ILMN_1666893 | TRIML2 | 0.84 | 0.13 | 0.85 | 0.13 | 0.49 |
| ILMN_1652490 | MANSC1 | 0.43 | 0.95 | 0.12 | 0.45 | 0.49 |
| ILMN_3268938 | LOC100128096 | 0.59 | 0.66 | 0.25 | 0.46 | 0.49 |
| ILMN_2412571 | TUBGCP6 | 0.62 | 0.69 | 0.42 | 0.21 | 0.49 |
| ILMN_2150661 | NUB1 | 0.45 | 0.42 | 0.66 | 0.42 | 0.49 |
| ILMN_1813658 | LOC643009 | 0.39 | 0.60 | 0.58 | 0.39 | 0.49 |
| ILMN_2206126 | RAET1L | 0.88 | 0.36 | 0.37 | 0.35 | 0.49 |
| ILMN_2276431 | DPH2 | 0.49 | 0.37 | 0.52 | 0.58 | 0.49 |
| ILMN_1687526 | LOC652773 | 0.53 | 0.37 | 0.31 | 0.74 | 0.49 |
| ILMN_1660624 | LIMK2 | 0.55 | 0.26 | 0.66 | 0.49 | 0.49 |
| ILMN_1693319 | EYA4 | 0.41 | 0.59 | 0.61 | 0.35 | 0.49 |
| ILMN_1651984 | LOC653648 | 0.42 | 0.70 | 0.42 | 0.42 | 0.49 |
| ILMN_1735438 | GPM6B | 0.56 | 0.32 | 0.59 | 0.49 | 0.49 |
| ILMN_1673805 | GIP | 0.78 | 0.40 | 0.40 | 0.40 | 0.49 |
| ILMN_1673804 | ZNF426 | 0.85 | 0.33 | 0.46 | 0.34 | 0.49 |
| ILMN_2220735 | CLEC3A | 0.90 | 0.15 | 0.78 | 0.15 | 0.49 |
