# Supplementary material for: Alu Elements in ANRIL Non-Coding RNA at Chromosome 9p21 Modulate Atherogenic Cell Functions through Trans-Regulation of Gene Networks
Source: PLoS Genet. 2013 Jul 4;9(7):e1003588. doi: 10.1371/journal.pgen.1003588 (PMC3701717; doi:10.1371/journal.pgen.1003588)
Supplement: Table S2 — ANRIL target-genes with average up-regulation >2 fold compared to vector control (n = 708). (DOC) [file pgen.1003588.s011.doc]

**Table S2. *ANRIL* target-genes with average up-regulation > 2 fold compared to vector control (n = 708).**

|  |  | Fold change of expression compared to vector control  sorted by average expression change | | | | |
| --- | --- | --- | --- | --- | --- | --- |
| Probe-ID | Symbol | ANRIL 1 | ANRIL2 | ANRIL3 | ANRIL4 | ANRIL avg |
| ILMN_2376403 | TSC22D3 | 1.00 | 514.48 | 1.00 | 253.08 | 192.39 |
| ILMN_2225537 | PTGR1 | 2.29 | 263.16 | 6.28 | 160.45 | 108.04 |
| ILMN_1751326 | FAM162B | 1.00 | 153.43 | 1.00 | 99.16 | 63.65 |
| ILMN_1704531 | PTGR1 | 1.00 | 155.25 | 2.73 | 88.82 | 61.95 |
| ILMN_1748124 | TSC22D3 | 1.03 | 137.84 | 1.11 | 84.86 | 56.21 |
| ILMN_1667476 | LTBR | 1.00 | 112.40 | 1.00 | 62.63 | 44.26 |
| ILMN_2234187 | CDO1 | 16.82 | 51.68 | 5.64 | 90.43 | 41.14 |
| ILMN_1772286 | OCIAD2 | 1.00 | 61.03 | 1.00 | 63.05 | 31.52 |
| ILMN_1675797 | EPDR1 | 6.95 | 53.16 | 6.81 | 53.13 | 30.01 |
| ILMN_2080080 | MAP7D2 | 1.00 | 76.13 | 1.00 | 38.65 | 29.19 |
| ILMN_1758164 | STC1 | 1.87 | 98.95 | 1.44 | 9.56 | 27.95 |
| ILMN_1713058 | PSTPIP2 | 4.82 | 50.41 | 31.00 | 25.46 | 27.92 |
| ILMN_1715401 | MT1G | 39.20 | 0.46 | 66.65 | 1.69 | 27.00 |
| ILMN_1701918 | KLHDC9 | 3.27 | 69.76 | 4.32 | 28.92 | 26.57 |
| ILMN_1700306 | OCIAD2 | 0.48 | 60.12 | 1.15 | 44.00 | 26.44 |
| ILMN_1785170 | ARMCX2 | 2.89 | 83.84 | 1.65 | 16.89 | 26.32 |
| ILMN_1844593 | | 1.00 | 38.10 | 1.00 | 23.75 | 15.96 |
| ILMN_2120247 | SLC2A10 | 1.72 | 33.52 | 0.79 | 27.49 | 15.88 |
| ILMN_2410523 | DDR2 | 1.00 | 34.66 | 1.00 | 23.20 | 14.97 |
| ILMN_1751328 | FAM83H | 1.00 | 34.40 | 1.00 | 20.99 | 14.35 |
| ILMN_1716733 | MYOM2 | 0.76 | 33.85 | 1.51 | 20.54 | 14.17 |
| ILMN_1720838 | DECR1 | 0.79 | 28.60 | 0.89 | 25.54 | 13.95 |
| ILMN_1754538 | C10orf58 | 1.60 | 29.94 | 1.20 | 22.67 | 13.85 |
| ILMN_1673704 | INA | 1.32 | 28.80 | 4.08 | 19.30 | 13.37 |
| ILMN_2214473 | ARHGEF5L | 1.00 | 34.11 | 1.00 | 15.50 | 12.90 |
| ILMN_2138765 | PLIN2 | 4.23 | 14.14 | 1.13 | 31.84 | 12.84 |
| ILMN_2234697 | BEX1 | 1.36 | 17.75 | 0.04 | 31.20 | 12.59 |
| ILMN_2405470 | KLHDC9 | 1.00 | 29.38 | 2.89 | 13.01 | 11.57 |
| ILMN_1740234 | GSTO2 | 1.00 | 14.50 | 1.00 | 29.13 | 11.41 |
| ILMN_1708093 | ARHGEF5 | 1.00 | 23.67 | 1.00 | 18.07 | 10.94 |
| ILMN_3249032 | EPCAM | 0.56 | 25.58 | 0.56 | 15.52 | 10.55 |
| ILMN_1682658 | EPM2AIP1 | 1.00 | 18.38 | 1.00 | 21.45 | 10.46 |
| ILMN_1658677 | DTX3 | 1.00 | 25.73 | 1.00 | 12.42 | 10.04 |
| ILMN_1653055 | SOX3 | 2.08 | 32.12 | 1.00 | 4.58 | 9.95 |
| ILMN_1709257 | DSCR6 | 1.00 | 32.66 | 1.00 | 4.03 | 9.67 |
| ILMN_2276952 | TSC22D3 | 1.00 | 28.05 | 1.00 | 8.34 | 9.60 |
| ILMN_1811364 | SGPP2 | 0.60 | 23.52 | 0.82 | 13.27 | 9.55 |
| ILMN_1677261 | LZTS1 | 1.00 | 25.55 | 1.00 | 8.14 | 8.92 |
| ILMN_2160210 | TACSTD1 | 0.29 | 22.03 | 0.29 | 12.71 | 8.83 |
| ILMN_1801045 | SPIN3 | 5.30 | 13.25 | 1.18 | 15.22 | 8.74 |
| ILMN_1682774 | C13orf27 | 1.57 | 13.70 | 2.31 | 16.63 | 8.55 |
| ILMN_1710644 | MARVELD3 | 1.00 | 20.16 | 2.63 | 9.22 | 8.25 |
| ILMN_2363658 | PXDN | 1.13 | 13.94 | 3.31 | 13.70 | 8.02 |
| ILMN_1737252 | NRG1 | 1.00 | 20.85 | 1.00 | 9.20 | 8.01 |
| ILMN_1786720 | PROM1 | 1.00 | 25.00 | 1.00 | 4.20 | 7.80 |
| ILMN_1809566 | ZSCAN16 | 4.64 | 12.36 | 0.92 | 13.17 | 7.77 |
| ILMN_1671568 | ECHDC2 | 1.72 | 15.85 | 1.55 | 10.80 | 7.48 |
| ILMN_2134974 | RAB38 | 0.92 | 17.45 | 1.78 | 9.64 | 7.45 |
| ILMN_2160209 | TACSTD1 | 1.46 | 16.72 | 0.90 | 10.47 | 7.39 |
| ILMN_1701455 | FBXO6 | 3.40 | 15.72 | 2.63 | 7.69 | 7.36 |
| ILMN_1716465 | RBP7 | 1.48 | 18.27 | 1.40 | 7.42 | 7.14 |
| ILMN_3237946 | LOC100134134 | 0.73 | 12.22 | 2.97 | 12.49 | 7.10 |
| ILMN_1714700 | TRIB2 | 0.63 | 16.10 | 1.15 | 10.39 | 7.07 |
| ILMN_2058795 | PGCP | 1.00 | 17.72 | 1.00 | 8.06 | 6.94 |
| ILMN_1703926 | PTGER2 | 1.00 | 12.46 | 1.00 | 12.63 | 6.77 |
| ILMN_1722524 | GALNT14 | 1.00 | 11.53 | 1.00 | 13.40 | 6.73 |
| ILMN_1774330 | WSCD1 | 1.04 | 16.22 | 1.00 | 8.63 | 6.72 |
| ILMN_2315789 | PTPRD | 1.13 | 11.59 | 1.00 | 13.15 | 6.72 |
| ILMN_1718766 | MT1F | 3.61 | 6.59 | 4.20 | 12.39 | 6.70 |
| ILMN_1739222 | ETV5 | 0.85 | 12.69 | 0.85 | 11.49 | 6.47 |
| ILMN_1708516 | PRTFDC1 | 1.53 | 10.59 | 1.38 | 11.74 | 6.31 |
| ILMN_1804007 | NANOS3 | 1.00 | 15.68 | 2.12 | 6.44 | 6.31 |
| ILMN_1713384 | LOC654096 | 1.00 | 11.57 | 1.14 | 11.48 | 6.30 |
| ILMN_2301722 | PDE8B | 1.23 | 14.35 | 1.34 | 8.20 | 6.28 |
| ILMN_1755638 | TAF7L | 1.00 | 8.62 | 1.00 | 14.18 | 6.20 |
| ILMN_1806754 | GLDC | 3.31 | 10.37 | 3.15 | 7.73 | 6.14 |
| ILMN_2413833 | TOX3 | 1.00 | 20.58 | 1.00 | 1.97 | 6.14 |
| ILMN_1696767 | SERP2 | 0.62 | 9.70 | 6.18 | 7.33 | 5.96 |
| ILMN_2135709 | C8orf47 | 1.00 | 11.74 | 2.14 | 8.47 | 5.84 |
| ILMN_1749327 | MAPK13 | 1.26 | 12.31 | 1.13 | 8.58 | 5.82 |
| ILMN_1753931 | CDO1 | 2.79 | 7.67 | 1.60 | 11.21 | 5.82 |
| ILMN_1707286 | FLJ22662 | 1.00 | 3.48 | 2.26 | 16.53 | 5.82 |
| ILMN_1702009 | SV2A | 2.60 | 8.73 | 1.12 | 10.78 | 5.81 |
| ILMN_3299520 | PRKCB | 1.20 | 9.43 | 2.11 | 10.28 | 5.75 |
| ILMN_1712774 | IRS4 | 1.06 | 11.99 | 0.63 | 8.87 | 5.64 |
| ILMN_1713141 | LOC389641 | 1.00 | 10.61 | 2.27 | 8.57 | 5.61 |
| ILMN_1655611 | TSHZ2 | 1.44 | 9.58 | 1.12 | 10.23 | 5.59 |
| ILMN_1676563 | HTRA1 | 1.23 | 12.24 | 0.95 | 7.93 | 5.59 |
| ILMN_1746265 | SARM1 | 1.03 | 10.10 | 1.00 | 10.10 | 5.56 |
| ILMN_1715482 | ULK2 | 1.85 | 11.51 | 2.31 | 6.44 | 5.53 |
| ILMN_1722718 | BMP2 | 0.78 | 7.70 | 1.15 | 12.26 | 5.47 |
| ILMN_1666222 | PHACTR3 | 1.00 | 8.16 | 1.00 | 11.56 | 5.43 |
| ILMN_1746359 | RERG | 1.00 | 13.40 | 1.00 | 6.28 | 5.42 |
| ILMN_1668039 | GYPC | 1.00 | 18.49 | 1.00 | 1.00 | 5.37 |
| ILMN_1770653 | MAL2 | 3.43 | 9.86 | 5.18 | 2.88 | 5.34 |
| ILMN_1759792 | CLIP4 | 1.24 | 11.34 | 2.30 | 6.36 | 5.31 |
| ILMN_3251415 | RBM43 | 1.00 | 10.74 | 1.00 | 8.42 | 5.29 |
| ILMN_2149164 | SFRP1 | 1.51 | 2.04 | 2.52 | 14.92 | 5.25 |
| ILMN_1677439 | GLS2 | 0.94 | 11.66 | 0.94 | 6.94 | 5.12 |
| ILMN_2388547 | EPSTI1 | 4.43 | 6.09 | 4.93 | 4.92 | 5.09 |
| ILMN_2124802 | MT1H | 5.60 | 1.00 | 11.21 | 2.42 | 5.06 |
| ILMN_2065690 | GRAMD3 | 1.00 | 8.63 | 1.00 | 9.52 | 5.04 |
| ILMN_1736539 | ALDH1L2 | 0.89 | 13.60 | 0.41 | 5.23 | 5.03 |
| ILMN_2100458 | RFESD | 4.12 | 7.01 | 2.06 | 6.93 | 5.03 |
| ILMN_1771832 | PARP6 | 1.37 | 6.59 | 5.41 | 6.68 | 5.01 |
| ILMN_1673232 | LASS1 | 3.02 | 10.11 | 0.85 | 5.89 | 4.97 |
| ILMN_1770940 | CDH1 | 1.00 | 11.47 | 1.00 | 6.22 | 4.92 |
| ILMN_2197128 | OSR1 | 1.00 | 7.84 | 1.00 | 9.82 | 4.91 |
| ILMN_1661994 | ESRRG | 0.69 | 6.94 | 0.69 | 11.21 | 4.88 |
| ILMN_2051972 | GPC3 | 0.86 | 14.88 | 0.25 | 3.12 | 4.78 |
| ILMN_1788813 | RASL10B | 1.00 | 8.39 | 1.00 | 8.21 | 4.65 |
| ILMN_1673521 | KISS1R | 2.29 | 12.53 | 2.35 | 1.41 | 4.65 |
| ILMN_1663351 | SLC2A10 | 1.00 | 9.05 | 1.00 | 7.44 | 4.62 |
| ILMN_1679158 | FAAH2 | 1.36 | 8.98 | 0.81 | 7.21 | 4.59 |
| ILMN_1874323 | | 4.47 | 6.62 | 4.23 | 2.86 | 4.55 |
| ILMN_1688075 | OTX2 | 1.00 | 7.36 | 1.00 | 8.66 | 4.51 |
| ILMN_2325185 | TYSND1 | 3.53 | 7.92 | 1.38 | 4.97 | 4.45 |
| ILMN_1691731 | PARP14 | 1.00 | 7.30 | 1.00 | 8.47 | 4.44 |
| ILMN_1677723 | ANGPT1 | 1.73 | 2.59 | 1.00 | 12.14 | 4.37 |
| ILMN_1749478 | TCEAL3 | 0.65 | 8.84 | 0.42 | 7.30 | 4.30 |
| ILMN_3243593 | LOC100008588 | 11.22 | 1.92 | 2.99 | 1.03 | 4.29 |
| ILMN_3250850 | RFESD | 2.44 | 5.90 | 2.65 | 6.14 | 4.28 |
| ILMN_1739496 | PRRX1 | 1.00 | 7.87 | 1.97 | 6.23 | 4.27 |
| ILMN_2389528 | MTL5 | 1.61 | 8.83 | 1.90 | 4.68 | 4.26 |
| ILMN_3307668 | MKRN3 | 1.00 | 8.57 | 1.00 | 6.44 | 4.25 |
| ILMN_1765801 | GAA | 1.58 | 9.85 | 0.66 | 4.77 | 4.21 |
| ILMN_2122103 | ETS1 | 1.00 | 9.08 | 1.00 | 5.75 | 4.21 |
| ILMN_1680757 | LRRC26 | 1.62 | 10.11 | 2.03 | 2.98 | 4.18 |
| ILMN_1668863 | LYPD1 | 0.36 | 8.40 | 0.20 | 7.74 | 4.18 |
| ILMN_1812096 | CADM4 | 2.85 | 7.93 | 1.00 | 4.88 | 4.16 |
| ILMN_1741406 | HOOK1 | 2.18 | 7.27 | 1.23 | 5.89 | 4.14 |
| ILMN_1811363 | NOVA1 | 2.75 | 4.59 | 0.86 | 8.28 | 4.12 |
| ILMN_2249018 | LOC389816 | 1.74 | 9.90 | 2.10 | 2.63 | 4.09 |
| ILMN_1737631 | PAQR6 | 1.14 | 8.59 | 1.34 | 5.16 | 4.06 |
| ILMN_1784783 | NME5 | 1.00 | 7.54 | 1.00 | 6.66 | 4.05 |
| ILMN_3241441 | MEGF6 | 4.36 | 1.00 | 7.81 | 2.98 | 4.04 |
| ILMN_1664154 | LOC440030 | 1.00 | 7.14 | 1.00 | 6.97 | 4.03 |
| ILMN_1873278 | LOC731895 | 1.31 | 6.35 | 0.90 | 7.53 | 4.02 |
| ILMN_1795298 | GPER | 2.03 | 4.32 | 1.03 | 8.38 | 3.94 |
| ILMN_2363450 | NOXO1 | 8.43 | 1.00 | 4.76 | 1.53 | 3.93 |
| ILMN_1690646 | SATB1 | 1.76 | 7.61 | 1.86 | 4.48 | 3.93 |
| ILMN_3310326 | MIR221 | 1.00 | 7.95 | 1.00 | 5.77 | 3.93 |
| ILMN_1747627 | ABCA2 | 5.19 | 6.20 | 3.15 | 1.16 | 3.92 |
| ILMN_1659688 | LGALS3BP | 1.00 | 8.34 | 0.68 | 5.66 | 3.92 |
| ILMN_1671686 | EPB49 | 1.11 | 8.49 | 1.75 | 4.29 | 3.91 |
| ILMN_1787981 | MFAP2 | 0.50 | 5.72 | 3.12 | 6.17 | 3.88 |
| ILMN_2139761 | LIMCH1 | 1.59 | 6.84 | 1.19 | 5.90 | 3.88 |
| ILMN_2410783 | GAA | 2.14 | 8.60 | 0.78 | 3.98 | 3.88 |
| ILMN_1734190 | TCEAL3 | 0.68 | 7.35 | 0.70 | 6.49 | 3.80 |
| ILMN_1807689 | PKNOX2 | 1.43 | 8.65 | 0.61 | 4.53 | 3.80 |
| ILMN_3308961 | MIR1974 | 5.69 | 2.00 | 4.83 | 2.66 | 3.80 |
| ILMN_1712707 | ABHD8 | 1.43 | 5.82 | 1.53 | 6.25 | 3.76 |
| ILMN_1717381 | HOXD1 | 4.40 | 2.69 | 2.13 | 5.57 | 3.70 |
| ILMN_2406035 | LAMA3 | 0.84 | 7.80 | 0.45 | 5.70 | 3.70 |
| ILMN_3237448 | BEND5 | 1.08 | 6.54 | 1.00 | 6.17 | 3.70 |
| ILMN_2387471 | FLJ22184 | 0.94 | 7.08 | 1.19 | 5.55 | 3.69 |
| ILMN_1733288 | C1RL | 1.38 | 5.71 | 1.00 | 6.66 | 3.69 |
| ILMN_1787658 | MTMR7 | 1.00 | 6.39 | 1.00 | 6.26 | 3.66 |
| ILMN_1694810 | PANX2 | 2.79 | 5.27 | 1.29 | 5.27 | 3.66 |
| ILMN_1713031 | PGCP | 1.14 | 7.37 | 1.00 | 5.09 | 3.65 |
| ILMN_1742003 | RHPN1 | 1.26 | 5.71 | 3.86 | 3.55 | 3.59 |
| ILMN_1732808 | TNRC9 | 1.00 | 10.60 | 1.00 | 1.60 | 3.55 |
| ILMN_1689046 | FLJ20273 | 1.00 | 5.93 | 1.68 | 5.58 | 3.55 |
| ILMN_1742330 | PLXNB1 | 1.00 | 6.40 | 1.04 | 5.75 | 3.55 |
| ILMN_1776121 | MGC42367 | 1.03 | 7.73 | 0.78 | 4.66 | 3.55 |
| ILMN_1709795 | RAC2 | 1.01 | 5.04 | 0.58 | 7.54 | 3.54 |
| ILMN_1683133 | KLF15 | 1.10 | 5.75 | 0.53 | 6.79 | 3.54 |
| ILMN_2384056 | GPER | 1.77 | 3.76 | 0.82 | 7.80 | 3.54 |
| ILMN_1667791 | PPFIA4 | 2.19 | 7.38 | 1.81 | 2.71 | 3.52 |
| ILMN_1745397 | OAS3 | 1.13 | 5.99 | 1.60 | 5.25 | 3.49 |
| ILMN_1690170 | CRABP2 | 1.29 | 9.31 | 1.08 | 2.26 | 3.48 |
| ILMN_1667825 | MLKL | 1.10 | 5.19 | 0.85 | 6.80 | 3.48 |
| ILMN_1906437 | | 1.02 | 8.87 | 0.74 | 3.31 | 3.48 |
| ILMN_1768595 | DLG4 | 1.79 | 4.38 | 4.45 | 3.24 | 3.47 |
| ILMN_3242105 | LOC100134073 | 0.25 | 6.71 | 0.43 | 6.46 | 3.46 |
| ILMN_1810725 | FAM129A | 0.81 | 8.01 | 0.75 | 4.18 | 3.44 |
| ILMN_1766573 | TERC | 4.82 | 2.59 | 3.42 | 2.90 | 3.43 |
| ILMN_2343097 | NCALD | 1.00 | 1.00 | 1.54 | 10.19 | 3.43 |
| ILMN_1727805 | SYNGR1 | 2.00 | 5.64 | 1.08 | 5.00 | 3.43 |
| ILMN_1716616 | MARVELD3 | 1.00 | 7.62 | 1.00 | 4.08 | 3.42 |
| ILMN_1748538 | ALDH1A2 | 0.14 | 6.56 | 0.16 | 6.83 | 3.42 |
| ILMN_1858599 | | 1.14 | 7.17 | 0.81 | 4.53 | 3.41 |
| ILMN_1655915 | MMP11 | 1.73 | 6.53 | 1.92 | 3.47 | 3.41 |
| ILMN_1659792 | HOXD9 | 1.00 | 4.01 | 1.61 | 6.96 | 3.40 |
| ILMN_1746148 | LRRC33 | 1.13 | 5.61 | 1.99 | 4.83 | 3.39 |
| ILMN_2054121 | C6orf126 | 1.18 | 8.23 | 0.89 | 3.08 | 3.35 |
| ILMN_1799589 | NOXA1 | 2.18 | 7.34 | 1.46 | 2.40 | 3.34 |
| ILMN_2407879 | SORBS2 | 1.00 | 5.84 | 1.00 | 5.53 | 3.34 |
| ILMN_1728496 | SYT9 | 0.93 | 6.12 | 0.67 | 5.64 | 3.34 |
| ILMN_1673566 | ADAMTS1 | 4.10 | 2.15 | 5.29 | 1.75 | 3.32 |
| ILMN_1684982 | PDK4 | 1.00 | 5.35 | 1.00 | 5.91 | 3.31 |
| ILMN_1765990 | KCNK2 | 3.88 | 6.70 | 1.00 | 1.60 | 3.30 |
| ILMN_1699585 | BHLHE22 | 1.00 | 2.65 | 1.00 | 8.45 | 3.28 |
| ILMN_2344650 | N4BP2L1 | 1.00 | 5.26 | 1.00 | 5.77 | 3.26 |
| ILMN_1653836 | C11orf41 | 2.09 | 5.74 | 1.99 | 3.10 | 3.23 |
| ILMN_1685496 | RGS7 | 0.45 | 6.44 | 0.77 | 5.25 | 3.23 |
| ILMN_2112128 | MAPK4 | 1.00 | 8.05 | 1.00 | 2.79 | 3.21 |
| ILMN_1683313 | ST3GAL1 | 1.75 | 5.99 | 1.80 | 3.24 | 3.19 |
| ILMN_1716407 | SORBS2 | 1.00 | 5.01 | 1.00 | 5.74 | 3.19 |
| ILMN_2255256 | MARVELD3 | 1.00 | 9.41 | 1.00 | 1.30 | 3.18 |
| ILMN_2330382 | PAQR6 | 1.00 | 5.84 | 1.00 | 4.85 | 3.17 |
| ILMN_1780591 | FAT3 | 0.67 | 6.70 | 0.84 | 4.44 | 3.16 |
| ILMN_1806603 | MESP1 | 0.87 | 5.63 | 0.45 | 5.56 | 3.13 |
| ILMN_1674498 | C7orf46 | 1.00 | 7.34 | 1.00 | 3.17 | 3.13 |
| ILMN_1797594 | NFAT5 | 2.40 | 3.99 | 2.17 | 3.94 | 3.13 |
| ILMN_3307916 | BMPER | 1.00 | 1.41 | 1.00 | 9.08 | 3.12 |
| ILMN_1808789 | MYO5C | 0.62 | 6.48 | 1.08 | 4.26 | 3.11 |
| ILMN_1658586 | LOC730012 | 1.74 | 4.12 | 2.80 | 3.78 | 3.11 |
| ILMN_3255144 | LOC100129104 | 1.81 | 5.51 | 2.34 | 2.76 | 3.11 |
| ILMN_1744887 | RLN2 | 2.59 | 3.30 | 2.94 | 3.56 | 3.10 |
| ILMN_1693853 | HHAT | 2.37 | 6.93 | 1.00 | 2.09 | 3.10 |
| ILMN_2298860 | HAGHL | 4.79 | 1.48 | 2.80 | 3.29 | 3.09 |
| ILMN_1792356 | DPYSL4 | 1.66 | 7.03 | 0.86 | 2.78 | 3.08 |
| ILMN_1788538 | NCALD | 1.00 | 1.00 | 1.00 | 9.27 | 3.07 |
| ILMN_3180409 | LOC100130093 | 1.97 | 5.44 | 2.16 | 2.65 | 3.06 |
| ILMN_1799280 | BDH1 | 1.00 | 5.16 | 1.00 | 5.05 | 3.05 |
| ILMN_1761322 | FHOD3 | 1.37 | 5.92 | 0.66 | 4.26 | 3.05 |
| ILMN_1665331 | AMT | 1.35 | 5.45 | 1.67 | 3.64 | 3.03 |
| ILMN_1775444 | FLJ12078 | 1.00 | 2.10 | 4.93 | 4.02 | 3.02 |
| ILMN_1814787 | ICA1 | 1.41 | 6.59 | 0.49 | 3.52 | 3.00 |
| ILMN_1718046 | ARNT2 | 0.73 | 5.66 | 0.93 | 4.69 | 3.00 |
| ILMN_2383707 | ALDH1A2 | 0.08 | 5.60 | 0.09 | 6.24 | 3.00 |
| ILMN_2221808 | DGCR5 | 3.13 | 4.51 | 1.29 | 3.07 | 3.00 |
| ILMN_2240597 | TCEA2 | 1.87 | 6.13 | 1.57 | 2.40 | 2.99 |
| ILMN_1860638 | | 1.23 | 6.71 | 1.49 | 2.49 | 2.98 |
| ILMN_1743345 | ARMCX4 | 1.04 | 5.16 | 1.00 | 4.69 | 2.97 |
| ILMN_1823231 | | 0.90 | 5.38 | 0.90 | 4.72 | 2.97 |
| ILMN_1764795 | FMN2 | 0.37 | 6.49 | 0.82 | 4.14 | 2.95 |
| ILMN_2199313 | NPDC1 | 2.86 | 4.79 | 2.79 | 1.36 | 2.95 |
| ILMN_1727790 | KHDRBS3 | 1.24 | 5.99 | 1.28 | 3.23 | 2.93 |
| ILMN_2375360 | COL4A5 | 0.97 | 5.13 | 0.82 | 4.79 | 2.93 |
| ILMN_1754584 | POLR2J4 | 2.23 | 3.11 | 3.38 | 2.98 | 2.93 |
| ILMN_2139970 | ALDH1A3 | 1.21 | 5.33 | 1.91 | 3.25 | 2.92 |
| ILMN_1715255 | MAGI2 | 1.00 | 6.46 | 1.05 | 3.17 | 2.92 |
| ILMN_1660125 | SFMBT2 | 2.20 | 2.60 | 2.33 | 4.50 | 2.90 |
| ILMN_1732577 | TMEM216 | 1.74 | 4.44 | 1.99 | 3.36 | 2.88 |
| ILMN_1815700 | WNT3A | 3.96 | 3.34 | 3.14 | 1.08 | 2.88 |
| ILMN_1862217 | | 0.83 | 4.19 | 0.99 | 5.49 | 2.88 |
| ILMN_1753933 | FAM150A | 1.00 | 7.24 | 1.00 | 2.20 | 2.86 |
| ILMN_1674694 | LOC643918 | 2.52 | 4.19 | 1.24 | 3.44 | 2.85 |
| ILMN_1745214 | LOC642393 | 1.74 | 2.08 | 4.89 | 2.68 | 2.85 |
| ILMN_1824898 | LOC728653 | 2.27 | 3.32 | 2.69 | 3.11 | 2.85 |
| ILMN_1746376 | SCARA3 | 0.80 | 5.87 | 1.34 | 3.37 | 2.85 |
| ILMN_1770643 | PEX26 | 2.88 | 3.17 | 3.46 | 1.86 | 2.84 |
| ILMN_1679728 | WDR87 | 3.34 | 2.44 | 2.32 | 3.27 | 2.84 |
| ILMN_1665526 | TCEA2 | 1.61 | 5.10 | 1.36 | 3.25 | 2.83 |
| ILMN_1743021 | CAMKK2 | 3.46 | 3.47 | 2.78 | 1.61 | 2.83 |
| ILMN_1696657 | LRRN2 | 1.56 | 4.06 | 1.55 | 4.09 | 2.81 |
| ILMN_1700831 | SLC27A2 | 1.22 | 5.09 | 1.02 | 3.91 | 2.81 |
| ILMN_1781198 | PPP1R3D | 1.31 | 4.50 | 1.57 | 3.81 | 2.80 |
| ILMN_2374115 | TFAP2A | 1.02 | 6.10 | 0.74 | 3.33 | 2.80 |
| ILMN_1704675 | IGSF1 | 2.88 | 6.30 | 1.00 | 1.00 | 2.79 |
| ILMN_2376859 | PDGFD | 1.00 | 5.53 | 1.00 | 3.65 | 2.79 |
| ILMN_1837167 | | 1.00 | 1.00 | 8.16 | 1.00 | 2.79 |
| ILMN_3290627 | LOC344328 | 1.17 | 1.73 | 2.88 | 5.36 | 2.79 |
| ILMN_1740728 | SMTNL2 | 2.73 | 3.18 | 1.94 | 3.24 | 2.77 |
| ILMN_1803094 | PDGFD | 0.70 | 5.45 | 0.43 | 4.45 | 2.76 |
| ILMN_2159453 | STXBP2 | 1.52 | 4.88 | 1.40 | 3.22 | 2.75 |
| ILMN_1843388 | | 2.56 | 3.44 | 2.13 | 2.86 | 2.75 |
| ILMN_3288018 | LOC645323 | 1.84 | 4.40 | 1.85 | 2.89 | 2.75 |
| ILMN_2406656 | GATA3 | 1.00 | 4.74 | 1.00 | 4.20 | 2.74 |
| ILMN_1795845 | CHST8 | 1.43 | 3.70 | 2.30 | 3.49 | 2.73 |
| ILMN_1688892 | LAMA3 | 1.04 | 4.78 | 1.43 | 3.67 | 2.73 |
| ILMN_1724832 | OVOL2 | 0.82 | 6.55 | 0.31 | 3.22 | 2.73 |
| ILMN_1672660 | MBP | 1.06 | 5.86 | 1.17 | 2.78 | 2.72 |
| ILMN_1912619 | | 1.01 | 2.54 | 2.48 | 4.84 | 2.72 |
| ILMN_3275388 | LOC340274 | 3.15 | 4.75 | 2.00 | 0.95 | 2.71 |
| ILMN_1753515 | SRR | 1.25 | 5.89 | 0.78 | 2.93 | 2.71 |
| ILMN_1699491 | DMRTA2 | 1.00 | 5.31 | 1.00 | 3.52 | 2.71 |
| ILMN_1744035 | SAPS2 | 2.47 | 1.54 | 2.61 | 4.18 | 2.70 |
| ILMN_2313946 | CPNE7 | 4.77 | 0.49 | 5.05 | 0.49 | 2.70 |
| ILMN_1653527 | LOC648358 | 0.98 | 2.79 | 5.05 | 1.96 | 2.70 |
| ILMN_1682332 | GYPC | 1.00 | 7.78 | 1.00 | 1.00 | 2.70 |
| ILMN_1746013 | SPOCK1 | 0.79 | 3.42 | 1.25 | 5.32 | 2.69 |
| ILMN_1752526 | RNF144B | 1.00 | 5.51 | 1.00 | 3.25 | 2.69 |
| ILMN_1735502 | FAM181B | 1.00 | 4.97 | 1.00 | 3.77 | 2.69 |
| ILMN_1713952 | C1orf106 | 1.22 | 4.82 | 1.35 | 3.35 | 2.68 |
| ILMN_2365569 | ICA1 | 1.68 | 5.18 | 0.92 | 2.95 | 2.68 |
| ILMN_2353431 | ANKRD16 | 2.12 | 3.22 | 0.77 | 4.60 | 2.68 |
| ILMN_1804444 | PDIA2 | 5.61 | 0.87 | 3.54 | 0.68 | 2.68 |
| ILMN_1770616 | C12orf56 | 3.31 | 0.74 | 5.92 | 0.74 | 2.68 |
| ILMN_1707925 | ABHD12B | 1.88 | 1.57 | 4.92 | 2.30 | 2.67 |
| ILMN_1765796 | ENO2 | 2.21 | 5.43 | 1.34 | 1.69 | 2.67 |
| ILMN_1804663 | THBS3 | 1.69 | 4.61 | 1.35 | 3.02 | 2.67 |
| ILMN_1695423 | CD9 | 1.84 | 3.41 | 1.63 | 3.77 | 2.66 |
| ILMN_1683891 | TFAP2C | 2.16 | 3.00 | 2.46 | 3.02 | 2.66 |
| ILMN_1811313 | SLIT3 | 1.00 | 4.57 | 1.00 | 4.06 | 2.66 |
| ILMN_2133187 | POL3S | 1.65 | 3.93 | 1.14 | 3.90 | 2.65 |
| ILMN_2319000 | MATK | 2.45 | 3.27 | 2.83 | 2.05 | 2.65 |
| ILMN_1885273 | | 0.62 | 1.65 | 3.72 | 4.60 | 2.65 |
| ILMN_1809931 | NDRG1 | 1.98 | 5.60 | 1.46 | 1.55 | 2.65 |
| ILMN_2203544 | OR2A9P | 1.00 | 6.93 | 1.00 | 1.63 | 2.64 |
| ILMN_1735877 | EFEMP1 | 1.19 | 4.80 | 1.23 | 3.31 | 2.63 |
| ILMN_1738523 | MYD88 | 0.61 | 5.42 | 0.61 | 3.86 | 2.63 |
| ILMN_1664369 | DHTKD1 | 1.78 | 4.10 | 1.30 | 3.31 | 2.62 |
| ILMN_1716224 | STARD4 | 2.31 | 3.33 | 2.08 | 2.76 | 2.62 |
| ILMN_2364768 | MYLK | 2.76 | 2.31 | 2.43 | 2.98 | 2.62 |
| ILMN_2387214 | PALM | 2.07 | 5.07 | 1.77 | 1.56 | 2.62 |
| ILMN_1712913 | UNC5A | 2.38 | 5.09 | 1.72 | 1.25 | 2.61 |
| ILMN_1709067 | SAMD11 | 1.69 | 3.14 | 1.98 | 3.62 | 2.61 |
| ILMN_1796663 | B4GALNT4 | 1.44 | 5.01 | 2.39 | 1.60 | 2.61 |
| ILMN_3301813 | LOC728470 | 2.04 | 3.08 | 2.63 | 2.68 | 2.61 |
| ILMN_1769704 | FLJ39632 | 3.20 | 3.22 | 2.12 | 1.85 | 2.60 |
| ILMN_1753584 | KRT8 | 0.75 | 5.17 | 1.12 | 3.35 | 2.60 |
| ILMN_1703374 | NAV1 | 1.83 | 5.52 | 1.00 | 2.02 | 2.59 |
| ILMN_2411781 | RYR1 | 1.00 | 5.22 | 1.61 | 2.54 | 2.59 |
| ILMN_1742534 | COL4A5 | 1.24 | 3.98 | 1.20 | 3.94 | 2.59 |
| ILMN_1709237 | EPHX2 | 0.28 | 6.55 | 0.28 | 3.25 | 2.59 |
| ILMN_1669898 | EGFL7 | 5.79 | 2.55 | 1.00 | 1.00 | 2.59 |
| ILMN_1785405 | SLC17A9 | 5.34 | 0.63 | 3.73 | 0.63 | 2.58 |
| ILMN_1740181 | RPL13L | 1.67 | 1.73 | 4.58 | 2.35 | 2.58 |
| ILMN_1857335 | | 2.09 | 4.78 | 1.98 | 1.45 | 2.57 |
| ILMN_2173611 | MT1E | 3.15 | 0.91 | 5.32 | 0.91 | 2.57 |
| ILMN_1766499 | HSPA2 | 2.68 | 3.28 | 0.55 | 3.79 | 2.57 |
| ILMN_1758523 | ABCA3 | 1.39 | 4.77 | 1.61 | 2.52 | 2.57 |
| ILMN_1806434 | PAQR5 | 1.49 | 4.64 | 0.95 | 3.19 | 2.57 |
| ILMN_1798855 | RASSF9 | 1.00 | 3.79 | 1.00 | 4.49 | 2.57 |
| ILMN_2094313 | ZDHHC1 | 0.98 | 4.84 | 1.08 | 3.38 | 2.57 |
| ILMN_1808117 | C1QL4 | 1.79 | 4.81 | 0.93 | 2.74 | 2.57 |
| ILMN_3301740 | LOC729887 | 1.22 | 3.72 | 0.92 | 4.40 | 2.56 |
| ILMN_1712523 | MAP6 | 1.00 | 4.19 | 3.53 | 1.52 | 2.56 |
| ILMN_1719543 | MAF | 0.70 | 3.71 | 0.57 | 5.21 | 2.55 |
| ILMN_1795309 | LOC391703 | 2.14 | 2.99 | 2.03 | 3.03 | 2.55 |
| ILMN_2400648 | SRGAP3 | 2.23 | 1.94 | 2.35 | 3.67 | 2.55 |
| ILMN_1747839 | ANKRD20A1 | 1.40 | 2.83 | 1.26 | 4.69 | 2.54 |
| ILMN_1722985 | ZNF658B | 1.64 | 2.83 | 2.27 | 3.43 | 2.54 |
| ILMN_2064917 | AGGF1 | 1.26 | 3.37 | 1.86 | 3.67 | 2.54 |
| ILMN_1716019 | RHBDL3 | 0.95 | 5.06 | 1.29 | 2.86 | 2.54 |
| ILMN_2091217 | UNQ9433 | 1.60 | 6.24 | 0.99 | 1.32 | 2.54 |
| ILMN_1713807 | MAN1C1 | 1.80 | 5.23 | 0.93 | 2.18 | 2.53 |
| ILMN_1801090 | KRT222 | 1.00 | 3.58 | 1.00 | 4.55 | 2.53 |
| ILMN_1728885 | KIAA1644 | 2.43 | 3.85 | 2.29 | 1.55 | 2.53 |
| ILMN_1708337 | NOXO1 | 3.83 | 1.16 | 3.04 | 2.08 | 2.53 |
| ILMN_2069322 | LOC100132288 | 1.94 | 1.96 | 2.67 | 3.53 | 2.52 |
| ILMN_1832656 | | 3.32 | 2.68 | 1.00 | 3.09 | 2.52 |
| ILMN_1734600 | WWOX | 1.98 | 1.65 | 1.22 | 5.22 | 2.52 |
| ILMN_2367638 | CAMKK2 | 2.33 | 3.06 | 1.74 | 2.93 | 2.52 |
| ILMN_1767801 | SAMD10 | 1.08 | 2.58 | 3.64 | 2.72 | 2.51 |
| ILMN_1745949 | CEACAM21 | 1.83 | 2.18 | 3.70 | 2.30 | 2.50 |
| ILMN_1795285 | PHF15 | 1.46 | 3.39 | 1.38 | 3.79 | 2.50 |
| ILMN_1737935 | MACF1 | 1.41 | 3.53 | 1.06 | 3.99 | 2.50 |
| ILMN_1764754 | RAMP1 | 1.00 | 1.93 | 1.00 | 6.05 | 2.50 |
| ILMN_1667966 | C1orf24 | 0.62 | 5.82 | 0.79 | 2.71 | 2.49 |
| ILMN_1773427 | KANK1 | 0.67 | 4.80 | 0.23 | 4.16 | 2.47 |
| ILMN_1687247 | SPATA20 | 1.45 | 3.82 | 1.63 | 2.95 | 2.46 |
| ILMN_1778668 | TAGLN | 1.56 | 4.60 | 1.00 | 2.68 | 2.46 |
| ILMN_1780283 | C20orf201 | 1.00 | 4.98 | 1.00 | 2.85 | 2.46 |
| ILMN_1675617 | NT5M | 1.96 | 6.10 | 0.88 | 0.88 | 2.45 |
| ILMN_1725294 | LOC654127 | 0.65 | 4.21 | 1.21 | 3.74 | 2.45 |
| ILMN_1679299 | IGSF1 | 1.43 | 6.38 | 1.00 | 1.00 | 2.45 |
| ILMN_1662651 | LOC646943 | 2.09 | 1.58 | 2.84 | 3.28 | 2.45 |
| ILMN_1791447 | CXCL12 | 0.71 | 5.49 | 0.60 | 2.99 | 2.45 |
| ILMN_1714738 | SCMH1 | 2.22 | 4.27 | 1.10 | 2.19 | 2.45 |
| ILMN_1676631 | CCNO | 1.16 | 4.22 | 1.11 | 3.29 | 2.44 |
| ILMN_1728117 | NKX2-1 | 1.45 | 5.31 | 1.00 | 2.01 | 2.44 |
| ILMN_1698015 | TBX1 | 2.07 | 3.09 | 2.29 | 2.33 | 2.44 |
| ILMN_2261076 | NEDD9 | 1.00 | 4.41 | 1.00 | 3.35 | 2.44 |
| ILMN_1697189 | PNCK | 1.33 | 6.35 | 1.13 | 0.93 | 2.43 |
| ILMN_1883024 | LOC732424 | 3.18 | 2.20 | 1.95 | 2.40 | 2.43 |
| ILMN_1724941 | CDCP1 | 1.80 | 3.26 | 2.37 | 2.32 | 2.43 |
| ILMN_1781386 | WIPI1 | 0.72 | 4.70 | 0.72 | 3.61 | 2.43 |
| ILMN_1751228 | C20orf46 | 1.17 | 4.68 | 1.04 | 2.84 | 2.43 |
| ILMN_1759652 | C1orf61 | 1.00 | 3.38 | 1.00 | 4.34 | 2.43 |
| ILMN_1654013 | C17orf28 | 1.90 | 4.71 | 0.87 | 2.24 | 2.43 |
| ILMN_1769018 | PCSK4 | 2.59 | 2.80 | 2.29 | 2.02 | 2.43 |
| ILMN_1676288 | ACBD4 | 2.22 | 3.72 | 2.06 | 1.69 | 2.42 |
| ILMN_1751753 | IDH2 | 1.35 | 4.42 | 0.94 | 2.99 | 2.42 |
| ILMN_1663772 | SIX2 | 2.09 | 2.88 | 2.75 | 1.97 | 2.42 |
| ILMN_3236709 | C17orf93 | 1.00 | 3.89 | 1.00 | 3.79 | 2.42 |
| ILMN_1671971 | LOC644743 | 1.05 | 5.16 | 0.90 | 2.57 | 2.42 |
| ILMN_1671437 | MAP3K15 | 1.88 | 3.94 | 1.55 | 2.32 | 2.42 |
| ILMN_1798288 | MOBKL2C | 1.00 | 5.23 | 1.00 | 2.44 | 2.42 |
| ILMN_1758705 | IRX6 | 0.35 | 4.71 | 0.73 | 3.88 | 2.42 |
| ILMN_2350634 | EFEMP1 | 0.68 | 5.77 | 0.61 | 2.61 | 2.42 |
| ILMN_1813544 | OXCT1 | 0.41 | 4.47 | 1.35 | 3.43 | 2.41 |
| ILMN_1831106 | | 2.01 | 3.24 | 1.78 | 2.62 | 2.41 |
| ILMN_1752355 | MPEG1 | 2.13 | 1.08 | 4.95 | 1.49 | 2.41 |
| ILMN_1723358 | SCARA3 | 0.83 | 5.23 | 1.05 | 2.52 | 2.41 |
| ILMN_1664303 | HTATIP2 | 0.84 | 4.55 | 1.17 | 3.06 | 2.40 |
| ILMN_1770038 | LAMA1 | 1.00 | 4.26 | 1.00 | 3.35 | 2.40 |
| ILMN_3268199 | LOC100129117 | 2.29 | 2.50 | 2.74 | 2.08 | 2.40 |
| ILMN_1670504 | TMEM103 | 1.72 | 2.13 | 3.26 | 2.49 | 2.40 |
| ILMN_3193142 | LOC100127983 | 2.42 | 1.73 | 1.98 | 3.47 | 2.40 |
| ILMN_1765574 | TFAP2A | 0.98 | 4.40 | 1.02 | 3.19 | 2.40 |
| ILMN_1807439 | ALDH1A3 | 0.84 | 4.21 | 1.58 | 2.94 | 2.40 |
| ILMN_1673522 | MOCOS | 1.29 | 4.32 | 0.59 | 3.38 | 2.40 |
| ILMN_2404407 | ABAT | 1.00 | 2.94 | 1.00 | 4.64 | 2.39 |
| ILMN_1868047 | | 2.50 | 3.02 | 1.00 | 3.04 | 2.39 |
| ILMN_1765772 | MYO3A | 1.14 | 3.86 | 1.45 | 3.11 | 2.39 |
| ILMN_1654966 | SCARA3 | 0.73 | 4.99 | 1.27 | 2.56 | 2.39 |
| ILMN_3305942 | LOC729970 | 2.41 | 3.60 | 1.26 | 2.26 | 2.38 |
| ILMN_2263466 | ACADVL | 1.23 | 3.67 | 3.52 | 1.11 | 2.38 |
| ILMN_2193325 | MMP23B | 3.64 | 0.82 | 4.38 | 0.68 | 2.38 |
| ILMN_1691747 | KHDRBS3 | 0.99 | 4.43 | 1.19 | 2.89 | 2.38 |
| ILMN_1738742 | PLAT | 0.71 | 5.50 | 0.53 | 2.76 | 2.38 |
| ILMN_1766650 | FOXA1 | 1.11 | 4.47 | 0.64 | 3.29 | 2.37 |
| ILMN_1712272 | LOC653270 | 2.13 | 1.73 | 2.03 | 3.60 | 2.37 |
| ILMN_1678423 | SPA17 | 2.51 | 2.57 | 1.69 | 2.72 | 2.37 |
| ILMN_1689102 | C1QTNF6 | 2.12 | 2.10 | 2.82 | 2.44 | 2.37 |
| ILMN_1765109 | TNFRSF25 | 2.82 | 2.94 | 1.73 | 1.98 | 2.37 |
| ILMN_1652719 | TMCO5A | 2.73 | 3.28 | 2.65 | 0.82 | 2.37 |
| ILMN_2359710 | PTP4A3 | 1.84 | 3.51 | 2.29 | 1.84 | 2.37 |
| ILMN_1814917 | TLE2 | 1.00 | 6.43 | 1.00 | 1.00 | 2.36 |
| ILMN_1748803 | ZDHHC9 | 2.60 | 2.04 | 3.33 | 1.46 | 2.36 |
| ILMN_1666642 | LOC339047 | 2.75 | 2.11 | 2.20 | 2.37 | 2.36 |
| ILMN_1747078 | HYLS1 | 1.78 | 2.90 | 1.93 | 2.82 | 2.36 |
| ILMN_1774784 | UBOX5 | 2.90 | 1.72 | 2.81 | 2.00 | 2.36 |
| ILMN_1787813 | SLC5A3 | 2.04 | 2.34 | 3.67 | 1.38 | 2.36 |
| ILMN_2342695 | PDGFA | 2.91 | 1.17 | 4.46 | 0.87 | 2.35 |
| ILMN_1845076 | | 3.09 | 1.91 | 2.69 | 1.71 | 2.35 |
| ILMN_1660669 | LOC653073 | 1.57 | 4.05 | 1.76 | 2.01 | 2.35 |
| ILMN_1854383 | | 2.17 | 2.39 | 2.54 | 2.29 | 2.35 |
| ILMN_1859392 | | 2.49 | 1.95 | 1.78 | 3.15 | 2.34 |
| ILMN_1725946 | IRF6 | 1.34 | 3.02 | 1.33 | 3.67 | 2.34 |
| ILMN_1773312 | INADL | 1.09 | 3.72 | 1.91 | 2.65 | 2.34 |
| ILMN_1670975 | C8orf47 | 1.00 | 4.00 | 1.07 | 3.28 | 2.34 |
| ILMN_2325347 | B3GALNT1 | 4.71 | 1.00 | 1.00 | 2.63 | 2.34 |
| ILMN_2350801 | SLC25A29 | 1.22 | 4.60 | 1.62 | 1.89 | 2.33 |
| ILMN_1898453 | | 1.48 | 2.17 | 3.23 | 2.43 | 2.33 |
| ILMN_1847363 | LOC731835 | 1.19 | 4.36 | 0.70 | 3.07 | 2.33 |
| ILMN_1788439 | LOC441193 | 4.10 | 1.22 | 2.72 | 1.28 | 2.33 |
| ILMN_1705302 | FCGRT | 1.10 | 4.10 | 1.18 | 2.93 | 2.33 |
| ILMN_1721316 | TNFRSF10A | 1.00 | 4.42 | 1.00 | 2.86 | 2.32 |
| ILMN_1812031 | PALM | 1.77 | 3.49 | 1.71 | 2.31 | 2.32 |
| ILMN_1726981 | VEGFB | 1.93 | 3.18 | 1.80 | 2.37 | 2.32 |
| ILMN_1813350 | HSD11B2 | 1.87 | 3.53 | 2.88 | 1.00 | 2.32 |
| ILMN_1711124 | MARVELD2 | 0.94 | 3.68 | 0.77 | 3.87 | 2.32 |
| ILMN_2405156 | PPAP2C | 1.28 | 4.46 | 1.00 | 2.53 | 2.32 |
| ILMN_3247424 | ADAP1 | 2.46 | 3.41 | 1.30 | 2.07 | 2.31 |
| ILMN_1769150 | THAP5 | 1.89 | 1.58 | 3.09 | 2.70 | 2.31 |
| ILMN_1805200 | DNM1 | 1.31 | 2.99 | 1.82 | 3.13 | 2.31 |
| ILMN_1719660 | SMPDL3B | 1.00 | 3.10 | 1.00 | 4.13 | 2.31 |
| ILMN_1828967 | | 0.78 | 2.11 | 2.55 | 3.79 | 2.31 |
| ILMN_1661299 | TLE6 | 1.00 | 6.23 | 1.00 | 1.00 | 2.31 |
| ILMN_1814074 | PHKA2 | 1.76 | 2.60 | 2.30 | 2.57 | 2.31 |
| ILMN_1653292 | PFKFB4 | 1.75 | 4.15 | 1.43 | 1.89 | 2.31 |
| ILMN_1899387 | | 2.04 | 2.09 | 2.92 | 2.16 | 2.31 |
| ILMN_1720235 | ADSSL1 | 1.68 | 3.95 | 0.45 | 3.14 | 2.30 |
| ILMN_1660955 | LOC644419 | 1.19 | 2.13 | 2.81 | 3.08 | 2.30 |
| ILMN_2089752 | LOC285016 | 1.00 | 6.20 | 1.00 | 1.00 | 2.30 |
| ILMN_1790317 | RAB26 | 2.02 | 4.33 | 0.76 | 2.09 | 2.30 |
| ILMN_1787461 | RUNX3 | 1.62 | 3.14 | 2.13 | 2.31 | 2.30 |
| ILMN_1677590 | RAP2B | 1.42 | 2.43 | 3.64 | 1.71 | 2.30 |
| ILMN_1656171 | ADAMTS19 | 1.00 | 4.70 | 1.00 | 2.49 | 2.30 |
| ILMN_1765061 | OXER1 | 1.00 | 3.00 | 1.81 | 3.38 | 2.30 |
| ILMN_1784110 | PCTK3 | 1.27 | 5.09 | 0.73 | 2.09 | 2.30 |
| ILMN_1806752 | PLEKHH2 | 1.87 | 3.49 | 1.00 | 2.82 | 2.30 |
| ILMN_1667298 | LOC201229 | 1.01 | 3.62 | 1.00 | 3.53 | 2.29 |
| ILMN_1675435 | ANKRD16 | 1.56 | 3.28 | 1.36 | 2.96 | 2.29 |
| ILMN_3237755 | SSPO | 1.50 | 3.39 | 1.81 | 2.44 | 2.29 |
| ILMN_1695972 | CCDC89 | 0.58 | 2.56 | 0.87 | 5.12 | 2.29 |
| ILMN_1665319 | NRTN | 2.26 | 4.37 | 0.96 | 1.55 | 2.28 |
| ILMN_1800634 | NME4 | 1.70 | 3.10 | 1.39 | 2.95 | 2.28 |
| ILMN_1684346 | TNFAIP8L1 | 2.75 | 3.10 | 1.00 | 2.28 | 2.28 |
| ILMN_2077680 | CLDND2 | 0.99 | 3.07 | 1.72 | 3.35 | 2.28 |
| ILMN_1709091 | OXGR1 | 0.91 | 1.86 | 0.91 | 5.45 | 2.28 |
| ILMN_1802691 | WDR52 | 2.14 | 1.40 | 3.54 | 2.05 | 2.28 |
| ILMN_1702479 | HOXA9 | 0.79 | 2.42 | 0.79 | 5.12 | 2.28 |
| ILMN_1662675 | ADAM2 | 1.94 | 1.89 | 2.73 | 2.55 | 2.28 |
| ILMN_1902630 | | 2.70 | 1.40 | 1.55 | 3.45 | 2.27 |
| ILMN_2312606 | IRF5 | 0.91 | 3.56 | 1.19 | 3.43 | 2.27 |
| ILMN_1777740 | C8orf55 | 2.02 | 2.78 | 1.64 | 2.63 | 2.27 |
| ILMN_1728236 | SLFN12 | 1.00 | 2.01 | 1.35 | 4.72 | 2.27 |
| ILMN_1900154 | LOC731186 | 2.09 | 1.45 | 3.00 | 2.54 | 2.27 |
| ILMN_1867457 | | 1.62 | 2.63 | 2.50 | 2.32 | 2.27 |
| ILMN_1767362 | ADAMTS6 | 1.60 | 1.00 | 3.99 | 2.47 | 2.27 |
| ILMN_1664466 | KLHL9 | 0.97 | 3.32 | 1.23 | 3.53 | 2.26 |
| ILMN_3280402 | LOC100132510 | 1.00 | 2.89 | 2.22 | 2.92 | 2.26 |
| ILMN_1707181 | LOC644284 | 2.88 | 1.29 | 2.89 | 1.98 | 2.26 |
| ILMN_1737462 | OXR1 | 1.65 | 2.49 | 2.30 | 2.57 | 2.25 |
| ILMN_2092118 | FPR1 | 1.71 | 2.08 | 2.64 | 2.58 | 2.25 |
| ILMN_1788363 | MLH1 | 1.40 | 2.99 | 1.78 | 2.84 | 2.25 |
| ILMN_1723260 | ETV5 | 1.00 | 2.89 | 1.00 | 4.12 | 2.25 |
| ILMN_1711078 | CDC2L2 | 2.00 | 2.30 | 2.60 | 2.10 | 2.25 |
| ILMN_2388484 | MAP2 | 1.00 | 3.70 | 1.00 | 3.28 | 2.25 |
| ILMN_1674308 | LOC728308 | 2.14 | 2.69 | 1.69 | 2.46 | 2.25 |
| ILMN_1773781 | CD276 | 1.00 | 1.00 | 3.04 | 3.94 | 2.24 |
| ILMN_1717485 | LOC285556 | 2.17 | 2.43 | 2.28 | 2.08 | 2.24 |
| ILMN_1816646 | | 1.87 | 1.86 | 2.49 | 2.74 | 2.24 |
| ILMN_1777040 | FLJ35785 | 2.42 | 2.72 | 2.10 | 1.72 | 2.24 |
| ILMN_1761000 | ACER2 | 1.69 | 1.59 | 2.77 | 2.91 | 2.24 |
| ILMN_1665250 | EFEMP1 | 1.87 | 1.63 | 3.37 | 2.08 | 2.24 |
| ILMN_1719170 | WBSCR27 | 1.73 | 2.67 | 2.43 | 2.11 | 2.24 |
| ILMN_1879480 | | 1.38 | 3.42 | 1.81 | 2.33 | 2.23 |
| ILMN_1751020 | PACSIN1 | 1.07 | 3.95 | 0.82 | 3.09 | 2.23 |
| ILMN_1799150 | PPM1J | 3.40 | 1.95 | 2.63 | 0.94 | 2.23 |
| ILMN_1749304 | LOC643389 | 1.70 | 2.95 | 1.78 | 2.50 | 2.23 |
| ILMN_3243366 | C2orf55 | 0.70 | 5.36 | 0.62 | 2.25 | 2.23 |
| ILMN_2349071 | GPR64 | 1.74 | 4.17 | 1.34 | 1.68 | 2.23 |
| ILMN_3242152 | LAMB2L | 1.86 | 5.05 | 1.00 | 1.01 | 2.23 |
| ILMN_3275615 | LOC645249 | 1.33 | 3.59 | 1.06 | 2.94 | 2.23 |
| ILMN_1803691 | ARTN | 0.94 | 5.49 | 0.94 | 1.54 | 2.23 |
| ILMN_1673898 | ATG12 | 2.32 | 2.20 | 2.06 | 2.33 | 2.23 |
| ILMN_1695880 | LOX | 1.64 | 4.48 | 1.12 | 1.68 | 2.23 |
| ILMN_1673352 | IFITM2 | 1.18 | 4.18 | 1.59 | 1.96 | 2.23 |
| ILMN_1698772 | KRT6B | 1.29 | 1.26 | 4.29 | 2.07 | 2.23 |
| ILMN_3265237 | FLJ44342 | 0.57 | 2.23 | 1.72 | 4.40 | 2.23 |
| ILMN_2179717 | C9orf61 | 2.64 | 2.71 | 1.78 | 1.77 | 2.23 |
| ILMN_1898518 | | 0.68 | 5.14 | 0.75 | 2.34 | 2.23 |
| ILMN_1880834 | FBXO41 | 2.13 | 2.38 | 1.61 | 2.76 | 2.22 |
| ILMN_2205032 | MAGEE1 | 1.36 | 2.90 | 1.00 | 3.62 | 2.22 |
| ILMN_1791306 | C9orf103 | 1.10 | 3.93 | 1.02 | 2.83 | 2.22 |
| ILMN_2354930 | ANKRD7 | 1.85 | 2.88 | 2.50 | 1.64 | 2.22 |
| ILMN_3211641 | LOC100131688 | 2.13 | 2.61 | 2.66 | 1.48 | 2.22 |
| ILMN_1745318 | CREM | 2.42 | 2.13 | 2.56 | 1.76 | 2.22 |
| ILMN_1859672 | | 1.96 | 1.00 | 2.32 | 3.59 | 2.22 |
| ILMN_1719194 | IBSP | 1.63 | 3.24 | 2.05 | 1.94 | 2.22 |
| ILMN_1701483 | SYP | 0.54 | 2.87 | 0.54 | 4.89 | 2.21 |
| ILMN_1681260 | LOC643272 | 0.40 | 0.10 | 8.22 | 0.12 | 2.21 |
| ILMN_1832151 | | 2.48 | 2.33 | 2.32 | 1.70 | 2.21 |
| ILMN_1715654 | LOC653471 | 1.75 | 1.83 | 2.01 | 3.23 | 2.21 |
| ILMN_1758657 | LOC653876 | 1.70 | 2.05 | 2.61 | 2.47 | 2.21 |
| ILMN_1781536 | FAH | 1.29 | 3.76 | 1.04 | 2.73 | 2.21 |
| ILMN_1787815 | TRIB3 | 1.98 | 4.22 | 1.24 | 1.38 | 2.21 |
| ILMN_1690592 | DDX12 | 1.80 | 2.21 | 2.98 | 1.82 | 2.20 |
| ILMN_1756989 | EPHA8 | 1.37 | 2.51 | 1.82 | 3.10 | 2.20 |
| ILMN_1663716 | PCDH21 | 1.88 | 3.36 | 1.00 | 2.55 | 2.20 |
| ILMN_1759732 | RFNG | 2.06 | 2.93 | 1.59 | 2.21 | 2.20 |
| ILMN_1729417 | GNE | 1.55 | 2.60 | 1.90 | 2.74 | 2.20 |
| ILMN_1730945 | C19orf4 | 2.08 | 3.01 | 1.00 | 2.70 | 2.20 |
| ILMN_3246294 | LOC728819 | 0.26 | 3.73 | 0.26 | 4.52 | 2.19 |
| ILMN_2225135 | GCNT1 | 3.31 | 0.97 | 3.52 | 0.97 | 2.19 |
| ILMN_1714397 | CRYL1 | 1.76 | 4.82 | 0.37 | 1.82 | 2.19 |
| ILMN_1703412 | LATS2 | 3.09 | 2.10 | 2.14 | 1.44 | 2.19 |
| ILMN_1675130 | NFIC | 2.27 | 2.18 | 2.08 | 2.22 | 2.19 |
| ILMN_1659297 | FZD6 | 1.55 | 2.83 | 1.38 | 2.99 | 2.19 |
| ILMN_1840334 | | 1.41 | 1.43 | 2.94 | 2.97 | 2.19 |
| ILMN_1736527 | TFCP2L1 | 1.55 | 3.14 | 0.95 | 3.10 | 2.19 |
| ILMN_1665775 | MOSC2 | 1.07 | 3.58 | 1.29 | 2.80 | 2.18 |
| ILMN_1655950 | ZHX1 | 2.16 | 2.55 | 3.20 | 0.81 | 2.18 |
| ILMN_2110919 | EBF2 | 1.00 | 4.31 | 1.00 | 2.41 | 2.18 |
| ILMN_1699217 | C1orf96 | 1.93 | 2.43 | 1.82 | 2.53 | 2.18 |
| ILMN_1794707 | ATHL1 | 1.99 | 2.61 | 2.10 | 2.01 | 2.18 |
| ILMN_2403911 | ARFIP1 | 2.48 | 1.63 | 1.94 | 2.66 | 2.18 |
| ILMN_1700719 | ST7OT1 | 1.00 | 3.80 | 2.07 | 1.84 | 2.18 |
| ILMN_1655849 | GSC | 2.16 | 2.37 | 1.00 | 3.17 | 2.18 |
| ILMN_1750062 | PPARGC1A | 1.00 | 4.91 | 1.00 | 1.79 | 2.17 |
| ILMN_1673455 | RASAL2 | 2.42 | 2.97 | 1.08 | 2.23 | 2.17 |
| ILMN_3236428 | TMEM170B | 1.25 | 3.10 | 1.19 | 3.15 | 2.17 |
| ILMN_1741027 | GEFT | 1.43 | 2.49 | 1.87 | 2.89 | 2.17 |
| ILMN_1741729 | LOC653580 | 1.00 | 1.00 | 4.23 | 2.45 | 2.17 |
| ILMN_1683494 | TMEM154 | 2.49 | 2.37 | 1.31 | 2.51 | 2.17 |
| ILMN_1724194 | NPEPL1 | 1.32 | 3.21 | 1.46 | 2.67 | 2.17 |
| ILMN_1854557 | | 2.33 | 1.60 | 1.59 | 3.14 | 2.17 |
| ILMN_1787179 | MEGF11 | 2.29 | 2.48 | 2.15 | 1.72 | 2.16 |
| ILMN_2153916 | HSPA2 | 2.07 | 3.03 | 0.56 | 2.97 | 2.16 |
| ILMN_3242416 | WASH3P | 2.74 | 2.28 | 2.53 | 1.08 | 2.16 |
| ILMN_1844299 | | 1.00 | 1.00 | 5.38 | 1.25 | 2.16 |
| ILMN_1847461 | | 1.97 | 1.35 | 2.84 | 2.48 | 2.16 |
| ILMN_2322498 | RORA | 1.67 | 3.77 | 0.83 | 2.36 | 2.16 |
| ILMN_3305447 | LOC729898 | 1.78 | 1.84 | 2.14 | 2.87 | 2.16 |
| ILMN_3239700 | HOTAIR | 1.02 | 4.43 | 1.00 | 2.18 | 2.16 |
| ILMN_1750368 | LOC642946 | 2.23 | 1.87 | 2.32 | 2.20 | 2.16 |
| ILMN_1755643 | MGAT4A | 0.58 | 3.61 | 1.24 | 3.19 | 2.16 |
| ILMN_1721167 | MYT1 | 0.68 | 4.88 | 1.14 | 1.92 | 2.16 |
| ILMN_2059996 | MEGF8 | 1.63 | 2.40 | 2.51 | 2.09 | 2.16 |
| ILMN_1665024 | FRYL | 2.00 | 2.23 | 1.31 | 3.09 | 2.16 |
| ILMN_1825773 | | 1.78 | 2.84 | 1.85 | 2.15 | 2.16 |
| ILMN_1661155 | DDX12 | 1.26 | 1.77 | 2.08 | 3.50 | 2.15 |
| ILMN_1741180 | HEXDC | 1.33 | 2.81 | 1.54 | 2.94 | 2.15 |
| ILMN_1675124 | DDX17 | 2.03 | 2.15 | 2.58 | 1.85 | 2.15 |
| ILMN_1671114 | LOC388565 | 1.00 | 2.09 | 2.64 | 2.88 | 2.15 |
| ILMN_1727135 | FIBCD1 | 1.58 | 2.59 | 2.43 | 2.01 | 2.15 |
| ILMN_1687867 | LOC647954 | 0.60 | 3.88 | 1.32 | 2.81 | 2.15 |
| ILMN_3230160 | PCLO | 1.00 | 2.16 | 1.57 | 3.86 | 2.15 |
| ILMN_1724197 | LOC645563 | 0.80 | 1.83 | 2.57 | 3.38 | 2.14 |
| ILMN_2232368 | PCIF1 | 2.83 | 3.52 | 1.23 | 1.00 | 2.14 |
| ILMN_1682588 | LOC643461 | 3.35 | 2.75 | 1.47 | 1.00 | 2.14 |
| ILMN_1741674 | PPP1R9A | 1.18 | 3.79 | 1.00 | 2.58 | 2.14 |
| ILMN_3241665 | C1orf133 | 1.57 | 2.94 | 1.75 | 2.28 | 2.14 |
| ILMN_1735539 | AKAP11 | 1.00 | 1.95 | 2.33 | 3.26 | 2.14 |
| ILMN_2381168 | CTNNBIP1 | 3.00 | 2.42 | 1.58 | 1.54 | 2.13 |
| ILMN_1765781 | LOC653394 | 1.62 | 1.00 | 3.12 | 2.78 | 2.13 |
| ILMN_1767816 | APH1B | 0.95 | 2.35 | 0.46 | 4.77 | 2.13 |
| ILMN_2234956 | LEPR | 2.00 | 2.32 | 2.73 | 1.48 | 2.13 |
| ILMN_3285742 | LOC100131786 | 1.25 | 2.67 | 2.86 | 1.74 | 2.13 |
| ILMN_1742135 | SPAG16 | 3.00 | 1.00 | 2.17 | 2.34 | 2.13 |
| ILMN_1666665 | COL23A1 | 2.45 | 1.18 | 3.56 | 1.32 | 2.13 |
| ILMN_1887011 | | 1.98 | 1.23 | 2.12 | 3.17 | 2.13 |
| ILMN_1654313 | LOC730995 | 1.75 | 2.73 | 2.08 | 1.95 | 2.13 |
| ILMN_3300313 | P4HTM | 0.74 | 4.46 | 1.16 | 2.15 | 2.13 |
| ILMN_3241237 | LOC100132503 | 1.58 | 3.71 | 1.78 | 1.43 | 2.13 |
| ILMN_3245773 | PION | 1.00 | 4.57 | 1.00 | 1.93 | 2.13 |
| ILMN_3242498 | LOC727880 | 2.59 | 1.00 | 2.38 | 2.52 | 2.12 |
| ILMN_1712886 | LOC642325 | 2.29 | 2.54 | 1.85 | 1.81 | 2.12 |
| ILMN_2105643 | GPD2 | 1.35 | 2.72 | 2.09 | 2.33 | 2.12 |
| ILMN_1815673 | DKK3 | 1.10 | 2.74 | 1.00 | 3.65 | 2.12 |
| ILMN_1755290 | CEP170 | 1.00 | 2.00 | 2.75 | 2.72 | 2.12 |
| ILMN_1807372 | ADORA2A | 2.07 | 2.21 | 1.68 | 2.52 | 2.12 |
| ILMN_3308370 | MIR30B | 1.63 | 1.27 | 1.51 | 4.07 | 2.12 |
| ILMN_2376953 | KCNK2 | 1.35 | 5.11 | 1.00 | 1.00 | 2.12 |
| ILMN_1732151 | COL6A1 | 1.26 | 3.66 | 1.92 | 1.62 | 2.11 |
| ILMN_1769388 | GJB2 | 0.98 | 3.20 | 2.08 | 2.19 | 2.11 |
| ILMN_1786278 | FAM149A | 1.75 | 3.53 | 0.74 | 2.43 | 2.11 |
| ILMN_1863656 | | 1.77 | 1.45 | 2.69 | 2.54 | 2.11 |
| ILMN_2203588 | MYL5 | 1.37 | 3.89 | 1.32 | 1.86 | 2.11 |
| ILMN_1696434 | LAMA1 | 0.81 | 3.83 | 0.74 | 3.05 | 2.11 |
| ILMN_1708647 | LOC648979 | 1.72 | 1.89 | 2.18 | 2.65 | 2.11 |
| ILMN_2056606 | PPP1R1A | 1.06 | 4.05 | 1.45 | 1.87 | 2.11 |
| ILMN_2388445 | DLX1 | 1.43 | 2.28 | 1.37 | 3.34 | 2.11 |
| ILMN_1800031 | C19orf15 | 0.98 | 1.15 | 3.46 | 2.84 | 2.11 |
| ILMN_3309584 | TAAR3 | 1.00 | 2.17 | 2.42 | 2.83 | 2.10 |
| ILMN_1807212 | USP44 | 1.00 | 4.39 | 1.11 | 1.92 | 2.10 |
| ILMN_1652155 | DHX9 | 2.86 | 2.03 | 1.48 | 2.04 | 2.10 |
| ILMN_1779648 | HIST3H2A | 1.39 | 3.45 | 1.42 | 2.15 | 2.10 |
| ILMN_1866524 | | 2.01 | 1.84 | 3.41 | 1.15 | 2.10 |
| ILMN_1652445 | RAC1 | 0.62 | 4.74 | 0.54 | 2.49 | 2.10 |
| ILMN_1678781 | SNX26 | 1.21 | 2.19 | 1.85 | 3.13 | 2.10 |
| ILMN_2411864 | PHACTR3 | 1.00 | 2.82 | 1.00 | 3.57 | 2.10 |
| ILMN_1721758 | ID4 | 0.84 | 3.50 | 1.64 | 2.41 | 2.10 |
| ILMN_1738675 | PTPN6 | 1.00 | 3.17 | 1.00 | 3.21 | 2.10 |
| ILMN_1776153 | AGGF1 | 1.07 | 2.56 | 1.77 | 2.98 | 2.10 |
| ILMN_1813131 | LOC643431 | 1.00 | 2.81 | 1.52 | 3.04 | 2.09 |
| ILMN_3289011 | LOC343515 | 2.50 | 1.23 | 2.92 | 1.73 | 2.09 |
| ILMN_1688666 | HIST1H2BH | 1.36 | 2.22 | 1.18 | 3.61 | 2.09 |
| ILMN_1770537 | NGFRAP1 | 1.17 | 2.55 | 1.68 | 2.97 | 2.09 |
| ILMN_2367010 | GPR126 | 1.08 | 3.39 | 1.58 | 2.30 | 2.09 |
| ILMN_1661599 | DDIT4 | 1.62 | 4.13 | 1.05 | 1.56 | 2.09 |
| ILMN_2311537 | HMGA1 | 1.71 | 3.02 | 1.46 | 2.16 | 2.09 |
| ILMN_1679595 | GRIK5 | 3.18 | 1.43 | 1.50 | 2.24 | 2.09 |
| ILMN_1753773 | ANAPC11 | 2.79 | 2.15 | 1.00 | 2.41 | 2.09 |
| ILMN_3282829 | LOC727913 | 1.68 | 1.75 | 3.00 | 1.91 | 2.09 |
| ILMN_1716693 | LOC731002 | 1.68 | 1.76 | 3.27 | 1.64 | 2.09 |
| ILMN_3248764 | KIAA1908 | 1.84 | 3.17 | 1.00 | 2.33 | 2.08 |
| ILMN_1728478 | CXCL16 | 1.25 | 4.17 | 0.25 | 2.67 | 2.08 |
| ILMN_1751161 | COL7A1 | 0.92 | 1.76 | 1.65 | 4.00 | 2.08 |
| ILMN_1729144 | EBAG9 | 1.06 | 3.88 | 1.00 | 2.39 | 2.08 |
| ILMN_1868532 | | 1.65 | 1.53 | 2.34 | 2.80 | 2.08 |
| ILMN_1715669 | TP53I11 | 1.53 | 2.42 | 2.16 | 2.22 | 2.08 |
| ILMN_1904054 | | 1.00 | 4.94 | 1.00 | 1.38 | 2.08 |
| ILMN_1715647 | VANGL2 | 0.71 | 2.88 | 0.73 | 4.01 | 2.08 |
| ILMN_1725750 | LOC644695 | 1.95 | 2.23 | 2.28 | 1.86 | 2.08 |
| ILMN_1772064 | LOC54103 | 1.00 | 3.62 | 1.00 | 2.69 | 2.08 |
| ILMN_2400947 | CUGBP2 | 1.00 | 4.58 | 1.00 | 1.73 | 2.08 |
| ILMN_1786444 | LPL | 1.07 | 2.20 | 1.83 | 3.21 | 2.08 |
| ILMN_1707464 | MST1 | 1.03 | 3.43 | 1.81 | 2.03 | 2.08 |
| ILMN_1718182 | THRA | 1.72 | 2.08 | 2.15 | 2.35 | 2.08 |
| ILMN_1754076 | CACNA2D3 | 1.51 | 2.52 | 1.75 | 2.50 | 2.07 |
| ILMN_1694887 | LOC440345 | 2.43 | 2.02 | 2.35 | 1.48 | 2.07 |
| ILMN_2391976 | SLC45A4 | 1.00 | 2.47 | 1.00 | 3.81 | 2.07 |
| ILMN_1826285 | | 1.29 | 2.88 | 1.50 | 2.60 | 2.07 |
| ILMN_3235716 | LOC100133719 | 1.88 | 1.59 | 1.61 | 3.19 | 2.07 |
| ILMN_3279757 | LOC100132532 | 2.77 | 1.00 | 3.50 | 1.00 | 2.07 |
| ILMN_1748437 | LOC646568 | 1.92 | 2.07 | 2.85 | 1.42 | 2.07 |
| ILMN_3259148 | LOC100129095 | 1.43 | 2.12 | 3.80 | 0.91 | 2.07 |
| ILMN_3263375 | LOC100130604 | 2.92 | 2.11 | 1.98 | 1.25 | 2.07 |
| ILMN_1685854 | C5orf53 | 1.54 | 2.52 | 1.79 | 2.42 | 2.07 |
| ILMN_1737992 | CRAT | 1.90 | 3.05 | 1.59 | 1.72 | 2.07 |
| ILMN_1730549 | LOC652734 | 2.24 | 1.47 | 2.43 | 2.12 | 2.06 |
| ILMN_1712075 | SYNM | 1.07 | 2.66 | 1.27 | 3.25 | 2.06 |
| ILMN_1673755 | LOC648898 | 1.85 | 2.39 | 1.93 | 2.08 | 2.06 |
| ILMN_1720926 | PSMD5 | 1.00 | 1.15 | 1.00 | 5.10 | 2.06 |
| ILMN_1653607 | TXNDC2 | 1.91 | 1.33 | 2.76 | 2.23 | 2.06 |
| ILMN_2113728 | FLJ31568 | 1.00 | 3.05 | 1.59 | 2.59 | 2.06 |
| ILMN_1849148 | | 1.66 | 1.95 | 1.92 | 2.70 | 2.06 |
| ILMN_3282121 | LOC202781 | 1.01 | 2.85 | 1.26 | 3.11 | 2.06 |
| ILMN_1725338 | CLDN23 | 3.92 | 1.00 | 1.00 | 2.31 | 2.06 |
| ILMN_1679471 | LOC649990 | 1.22 | 2.07 | 2.90 | 2.03 | 2.06 |
| ILMN_2376263 | SMARCA1 | 0.49 | 2.63 | 1.20 | 3.89 | 2.05 |
| ILMN_3203801 | LOC442041 | 1.07 | 3.72 | 1.68 | 1.74 | 2.05 |
| ILMN_1665428 | GSDMD | 1.00 | 3.91 | 1.00 | 2.30 | 2.05 |
| ILMN_1771261 | SYNC1 | 2.24 | 2.44 | 2.78 | 0.75 | 2.05 |
| ILMN_1749338 | ANKRD29 | 0.69 | 4.81 | 0.69 | 2.02 | 2.05 |
| ILMN_1660306 | ITGA8 | 1.00 | 4.41 | 1.00 | 1.79 | 2.05 |
| ILMN_1773459 | SOX11 | 1.00 | 4.10 | 1.00 | 2.10 | 2.05 |
| ILMN_1898130 | | 1.22 | 1.82 | 3.20 | 1.96 | 2.05 |
| ILMN_1686679 | ZNF462 | 0.76 | 3.90 | 0.28 | 3.27 | 2.05 |
| ILMN_2280568 | ANKRD36 | 1.45 | 2.78 | 1.70 | 2.25 | 2.05 |
| ILMN_1826591 | | 1.58 | 1.38 | 2.17 | 3.06 | 2.05 |
| ILMN_1797191 | KIAA0040 | 1.16 | 3.32 | 0.93 | 2.78 | 2.05 |
| ILMN_2372136 | P4HTM | 0.62 | 4.16 | 1.35 | 2.06 | 2.05 |
| ILMN_2317751 | REC8 | 2.12 | 1.00 | 2.63 | 2.44 | 2.05 |
| ILMN_1669543 | LOC642950 | 0.99 | 3.93 | 2.52 | 0.75 | 2.05 |
| ILMN_1749834 | LOC388588 | 1.04 | 3.25 | 1.17 | 2.72 | 2.05 |
| ILMN_1888690 | | 2.00 | 2.11 | 2.46 | 1.61 | 2.05 |
| ILMN_1872967 | | 1.26 | 1.74 | 3.06 | 2.10 | 2.04 |
| ILMN_1718060 | OTUD7A | 1.80 | 1.31 | 2.15 | 2.91 | 2.04 |
| ILMN_1812968 | SOX18 | 1.53 | 2.47 | 2.10 | 2.07 | 2.04 |
| ILMN_1714741 | LOC346887 | 1.81 | 2.37 | 1.56 | 2.42 | 2.04 |
| ILMN_1773743 | LOC643435 | 1.65 | 1.25 | 2.19 | 3.07 | 2.04 |
| ILMN_2086890 | ANGPT1 | 0.60 | 2.08 | 0.60 | 4.88 | 2.04 |
| ILMN_1690884 | APOA1 | 1.03 | 3.87 | 1.00 | 2.25 | 2.04 |
| ILMN_1788232 | FGF20 | 1.00 | 4.39 | 1.00 | 1.76 | 2.04 |
| ILMN_3251756 | WRN | 2.56 | 1.72 | 1.74 | 2.12 | 2.04 |
| ILMN_2046611 | MCOLN3 | 1.09 | 3.03 | 1.46 | 2.55 | 2.04 |
| ILMN_1794818 | LHX8 | 1.74 | 4.25 | 0.61 | 1.54 | 2.03 |
| ILMN_1806010 | CUL9 | 1.51 | 3.52 | 0.72 | 2.38 | 2.03 |
| ILMN_1803825 | CXCL12 | 1.00 | 4.41 | 1.00 | 1.73 | 2.03 |
| ILMN_1708916 | PDZD4 | 1.13 | 2.27 | 3.62 | 1.10 | 2.03 |
| ILMN_1792409 | AMOT | 0.63 | 3.71 | 1.63 | 2.16 | 2.03 |
| ILMN_1782429 | TMEM56 | 1.37 | 3.37 | 1.07 | 2.31 | 2.03 |
| ILMN_1671970 | LOC649493 | 1.28 | 1.99 | 2.50 | 2.36 | 2.03 |
| ILMN_1685057 | SLC22A4 | 1.59 | 1.43 | 2.83 | 2.27 | 2.03 |
| ILMN_1660864 | RHBDL1 | 0.85 | 2.47 | 1.70 | 3.10 | 2.03 |
| ILMN_1782751 | GLI4 | 1.00 | 3.18 | 1.00 | 2.93 | 2.03 |
| ILMN_1810115 | KCTD2 | 1.65 | 1.32 | 2.13 | 3.01 | 2.03 |
| ILMN_1717727 | LOC284293 | 1.00 | 2.68 | 1.00 | 3.43 | 2.03 |
| ILMN_1660995 | AMH | 0.88 | 2.97 | 1.67 | 2.59 | 2.03 |
| ILMN_1803984 | MAK | 1.92 | 2.50 | 1.36 | 2.32 | 2.03 |
| ILMN_1744046 | DIAPH2 | 1.72 | 3.47 | 1.80 | 1.11 | 2.03 |
| ILMN_1746343 | LOC643985 | 1.12 | 2.98 | 1.62 | 2.38 | 2.02 |
| ILMN_1685052 | DNAH1 | 1.16 | 2.49 | 1.52 | 2.92 | 2.02 |
| ILMN_2047511 | CENTA1 | 1.91 | 2.38 | 1.82 | 1.97 | 2.02 |
| ILMN_1768953 | SDC1 | 1.66 | 1.64 | 2.31 | 2.47 | 2.02 |
| ILMN_1798779 | LSP1 | 1.61 | 1.76 | 2.61 | 2.11 | 2.02 |
| ILMN_1893523 | | 1.60 | 2.02 | 2.25 | 2.21 | 2.02 |
| ILMN_1791329 | FCRL2 | 1.33 | 0.96 | 3.11 | 2.68 | 2.02 |
| ILMN_2331231 | TNFRSF6B | 1.59 | 1.62 | 2.19 | 2.67 | 2.02 |
| ILMN_3177532 | CECR4 | 0.87 | 4.96 | 0.53 | 1.71 | 2.02 |
| ILMN_1725471 | GK | 1.61 | 2.37 | 1.65 | 2.44 | 2.02 |
| ILMN_1783909 | COL6A2 | 1.97 | 4.06 | 0.93 | 1.11 | 2.02 |
| ILMN_1719069 | FOXN4 | 1.34 | 4.52 | 0.72 | 1.48 | 2.01 |
| ILMN_1800033 | LOC649214 | 1.74 | 1.06 | 3.13 | 2.13 | 2.01 |
| ILMN_1850238 | | 1.46 | 2.70 | 1.37 | 2.51 | 2.01 |
| ILMN_1773940 | GPR161 | 1.50 | 3.44 | 0.81 | 2.29 | 2.01 |
| ILMN_1912827 | | 1.66 | 2.15 | 1.89 | 2.32 | 2.01 |
| ILMN_1785709 | LOC653887 | 1.57 | 1.61 | 2.37 | 2.47 | 2.01 |
| ILMN_1683824 | ANO7 | 1.75 | 2.82 | 1.89 | 1.57 | 2.01 |
