# Supplementary material for: Alu Elements in ANRIL Non-Coding RNA at Chromosome 9p21 Modulate Atherogenic Cell Functions through Trans-Regulation of Gene Networks
Source: PLoS Genet. 2013 Jul 4;9(7):e1003588. doi: 10.1371/journal.pgen.1003588 (PMC3701717; doi:10.1371/journal.pgen.1003588)
Supplement: Table S3 — Experimental setup and P-values of experiments shown in Figure 2. (DOC) [file pgen.1003588.s012.doc]

**Table S3.** Experimental setup and *P*-values of experiments shown in Figure 2.
