# Supplementary material for: Alu Elements in ANRIL Non-Coding RNA at Chromosome 9p21 Modulate Atherogenic Cell Functions through Trans-Regulation of Gene Networks
Source: PLoS Genet. 2013 Jul 4;9(7):e1003588. doi: 10.1371/journal.pgen.1003588 (PMC3701717; doi:10.1371/journal.pgen.1003588)
Supplement: Table S4 — ANRIL RACE primers. (DOC) [file pgen.1003588.s013.doc]

**Table S4.** *ANRIL* RACE primers.

| Primer Name | RACE | Primer | *ANRIL* exon | Sequence |
| --- | --- | --- | --- | --- |
| RCR Primer 1* | 5’ | 5’-primer |  | 5' –TCATACACATACGATTTAGGTGACACTAT AGAGCGGCCGCCTGCAGGAAA -3' |
| 5’ RACE_1 | 5’ | 3’-primer | 5 | 5’-CTTTGATCTCTGCTGTTGAATCAGAATG-3’ |
| 5’ RACE_2 | 5’ | 3’-primer | 6 | 5’-CTGACTCGGGAAAGGATTCCAsCACACC-3’ |
| RCR Primer 2* | 3’ | 3’-primer |  | 5’-TAGACTTAGAAATTAATACGACTCACTATAG  GCGCGCCACCG-3’ |
| 3’ RACE_1 | 3’ | 5’-primer | 5 | 5’-CATTCTGATTCAACAGCAGAGATCAAAG-3’ |
| 3’ RACE_2 | 3’ | 5’-primer | 6 | 5’-GGTGTGsTGGAATCCTTTCCCGAGTCAG-3’ |
| 3’ RACE_3 | 3’ | 5’-primer | 7 | 5’-GAGACACCACACCCGGCGGATAGAGAG-3’ |

s – C/G nucleotide since primer spans SNP rs10738605 (C/G)
* primers provided with the ExactSTART Eukaryotic mRNA 5´- & 3´-RACE Kit (Epicentre Biotechnologies)
