# Supplementary material for: Alu Elements in ANRIL Non-Coding RNA at Chromosome 9p21 Modulate Atherogenic Cell Functions through Trans-Regulation of Gene Networks
Source: PLoS Genet. 2013 Jul 4;9(7):e1003588. doi: 10.1371/journal.pgen.1003588 (PMC3701717; doi:10.1371/journal.pgen.1003588)
Supplement: Table S5 — Primers used for PCR amplification of ANRIL transcripts. (DOC) [file pgen.1003588.s014.doc]

**Table S5. Primers used for PCR amplification of *ANRIL* transcripts.**

| Primers | Primer | Sequence |
| --- | --- | --- |
| ANRIL Ex1_FWD | 5’-primer | 5’-GCCTCTGACGCGACATCTGG-3’ |
| ANRIL Ex7b_REV | 3’-primer | 5’-GGGTCGGGTAAAGGTAAACTCAG-3’ |
| ANRIL Ex13_REV | 3’-primer | 5’-AAACCCAACAAGATAGAGAAGCAGG-3’ |
| ANRIL Ex13b_REV | 3’-primer | 5’-TTCTTGCTATACTGTCCTCTGCCAC-3’ |
| ANRIL Ex20_REV | 3’-primer | 5’-GGTTGCAGTCCTGGTTCTGCC-3’ |
