# Supplementary material for: Alu Elements in ANRIL Non-Coding RNA at Chromosome 9p21 Modulate Atherogenic Cell Functions through Trans-Regulation of Gene Networks
Source: PLoS Genet. 2013 Jul 4;9(7):e1003588. doi: 10.1371/journal.pgen.1003588 (PMC3701717; doi:10.1371/journal.pgen.1003588)
Supplement: Table S6 — Quantitative RT-PCR primers and probes. (DOC) [file pgen.1003588.s015.doc]

**Table S6. Quantitative RT-PCR primers and probes.**

| Transcript/  NM number | Primer/ probe | Sequence/reference |
| --- | --- | --- |
| *ANRIL Ex7b* | 5’-primer | 5’-agaattcttgattctttgctttcc-3’ |
|  | 3’-primer | 5’-tccctagttttgaggactaagctact-3’ |
|  | probe | 5’F-CTCCCTCCTGGAAATCCTGCTCTTACCAGTC-3‘T |
| *ANRIL Ex7-13* | 5’-primer | 5’-GAACTCCCGACCTCGTGATTCGC-3’ |
|  | 3’-primer | 5’-CTTCGTAGGAAATTCCTAGCTCCGTAATC-3’ |
|  | probe | 5’F-AGTGCTGGGATTACAGGTGTGAGACACCACG-3’T |
| *ANRIL Ex10-13b* | 5’-primer | 5’-ctgtggccaccttggaga-3’ |
|  | 3’-primer | 5’-TGGCTTCCATAGCACCAACT-3’ |
|  | probe | 5’F-AGACACTGGAGGTACACAGGATTTGGGTGAAG-3’T |
| *ANRIL Ex18-19* |  | synonym *NR_003529* in |
| *ANRIL Ex1-5* |  | synonym *EU741058* in |
| *TSC22D3* | 5’-primer | 5‘-tggtggccatagacaacaag-3‘ |
| NM_198057.2 | 3’-primer | 5‘-CTCCACCTCCTCTCTCACAGCATAC-3‘ |
|  | probe | 5’F-CGAACAGGCCATGGATCTGGTGAAGAATC-3‘T |
| *COL3A1* | 5’-primer | 5‘-tcaaggctgaaggaaatagca-3‘ |
| NM_000090.3 | 3’-primer | 5‘-cattccccagtgtgtttcg-3‘ |
|  | probe | 5’F-ATTCACCTACACAGTTCTGGAGGATGGTTGCA-3‘T |
| *Beta actin* (*BA*) |  | published in |
| *GAPDH* | 5’-primer | 5’-CCGTCAAGGCTGAGAACGG-3’ |
| NM_002046 | 3’-primer | 5‘-ctcagcgccagcatcgc-3‘ |
|  | probe | 5’F- CATCTTCCAGGAGCGAGATCCCTCC-3‘T |
| *U1* | 5’-primer | 5‘-ATACTTACCTGGCAGGGGAGATACCA-3‘ |
|  | 3’-primer | 5’CAGGGGAAAGCGCGAACG-3‘ |
|  | probe | 5’F-CCAGGGCGAGGCTTATCCATTGCACT-3‘T |

probes: 5‘F-= 5‘FAMRA-; -3‘T = -3‘TAMRA

**Reference Supporting Information**1. Holdt LM, Beutner F, Scholz M, Gielen S, Gabel G, et al. (2010) ANRIL expression is associated with atherosclerosis risk at chromosome 9p21. Arterioscler Thromb Vasc Biol 30: 620-627.
