# Supplementary material for: Alu Elements in ANRIL Non-Coding RNA at Chromosome 9p21 Modulate Atherogenic Cell Functions through Trans-Regulation of Gene Networks
Source: PLoS Genet. 2013 Jul 4;9(7):e1003588. doi: 10.1371/journal.pgen.1003588 (PMC3701717; doi:10.1371/journal.pgen.1003588)
Supplement: Table S7 — Mapping of ChIP-seq reads. (DOC) [file pgen.1003588.s016.doc]

**Table S7. Mapping of ChIP-seq reads.**

| Protein | Condition (cell line) | Number of reads | Number of uniquely mapping reads |
| --- | --- | --- | --- |
| CBX7 | vector | 53,404,817 | 42,393,747 (79.4 %) |
| CBX7 | ANRIL | 50,886,470 | 40,790,641 (80.2 %) |
| SUZ12 | vector | 62,663,464 | 49,177,355 (78.5 %) |
| SUZ12 | ANRIL | 53,699,864 | 43,832,410 (81.6 %) |
| INPUT DNA | vector | 31,509,467 | 27,857,857 (88.4 %) |
| INPUT DNA | ANRIL | 30,348,091 | 26,864,560 (88.5 %) |
